# Emerging regulatory mechanisms and functions of biomolecular condensates: implications for therapeutic targets

**DOI:** 10.1038/s41392-024-02070-1

**Published:** 2025-01-06

**Authors:** Soyoung Jeon, Yeram Jeon, Ji-Youn Lim, Yujeong Kim, Boksik Cha, Wantae Kim

**Affiliations:** 1 Department of Life Science, University of Seoul, Seoul, South Korea; 2 New Drug Development Center, Daegu-Gyeongbuk Medical Innovation Foundation, Daegu, South Korea

**Keywords:** Cell biology, Molecular biology

## Abstract

Cells orchestrate their processes through complex interactions, precisely organizing biomolecules in space and time. Recent discoveries have highlighted the crucial role of biomolecular condensates—membrane-less assemblies formed through the condensation of proteins, nucleic acids, and other molecules—in driving efficient and dynamic cellular processes. These condensates are integral to various physiological functions, such as gene expression and intracellular signal transduction, enabling rapid and finely tuned cellular responses. Their ability to regulate cellular signaling pathways is particularly significant, as it requires a careful balance between flexibility and precision. Disruption of this balance can lead to pathological conditions, including neurodegenerative diseases, cancer, and viral infections. Consequently, biomolecular condensates have emerged as promising therapeutic targets, with the potential to offer novel approaches to disease treatment. In this review, we present the recent insights into the regulatory mechanisms by which biomolecular condensates influence intracellular signaling pathways, their roles in health and disease, and potential strategies for modulating condensate dynamics as a therapeutic approach. Understanding these emerging principles may provide valuable directions for developing effective treatments targeting the aberrant behavior of biomolecular condensates in various diseases.

## Introduction

In eukaryotic cells, organelle structures serve specific functions in cellular maintenance and homeostasis, providing spatiotemporal control in response to chemical changes and signaling pathways. Organelles are categorized into membrane-enclosed organelles (such as the nucleus and endoplasmic reticulum) and membrane-less organelles (such as PML and Cajal bodies). Both types compartmentalize and concentrate specific proteins, nucleic acids, and other molecules, enabling them to perform specialized functions. Membrane-less organelles are diverse in their physical properties, subcellular localization, and molecular composition.^1–4^ They have been initially referred to as cellular bodies, granules, speckles, aggregates, droplets, and puncta.^5–10^ The term “biomolecular condensates” has recently been introduced to describe these membrane-less compartments. One proposed mechanism for the formation of these condensates is liquid-liquid phase separation (LLPS), which enables dynamic and regulated compartmentalization.^5,11^ However, LLPS is not the only mechanism; other processes, such as direct polymerization, can also contribute to the formation of these condensates, as will be discussed later in this review. This broader understanding of condensate formation builds on earlier concepts, such as the idea proposed by Edmund Beecher Wilson in 1898, who suggested that the cytoplasm contains densely packed droplets with distinct chemical properties, behaving like a liquid emulsion. Despite this early observation, the detailed mechanisms underlying LLPS and related processes remained largely unexplored for many years, due to uncertainties about their physiological relevance and the limited experimental evidence available to elucidate the physicochemical properties of these assemblies.^12^

In 2009, Brangwynne et al. conducted groundbreaking research that provided pivotal physiological evidence supporting the concept of LLPS. Their study focused on RNA- and protein-rich P granules, also known as germ granules, within *Caenorhabditis elegans (C. elegans)* embryos. Through meticulous experimentation, they revealed that these P granules exhibit liquid-like properties, dynamically forming and deforming through phase separation. Remarkably, they observed behaviors such as dripping, fusion, and fission, indicative of the dynamic nature of these condensates.^13^ The significance of LLPS in cellular physiology was further highlighted by the discovery of similar phenomena occurring in the nucleoli of amphibian oocytes.^14^ These findings collectively emphasized the importance of LLPS as a fundamental biophysical mechanism contributing to the formation and dynamics of various non-membranous organelles within cells. The milestone research by Hyman group provided key insights into the behavior of biomolecular condensates, paving the way for further exploration of their roles in cellular function and disease pathology. Moreover, a significant milestone in LLPS research was the establishment of cell-free in vitro systems. These innovative experimental setups have allowed researchers to conduct comprehensive investigations into the physical properties of biomolecular condensates, facilitating a deeper understanding of their underlying mechanisms and their implications in various cellular processes and diseases.^4,13^

**Fig. 1:**
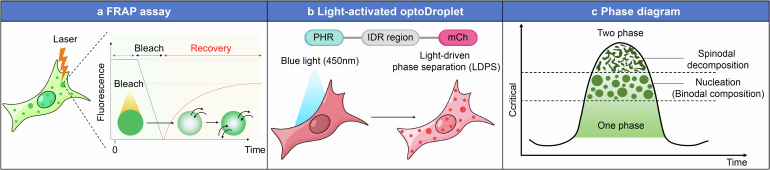
Techniques for investigating phase separation dynamics and phase transitions in biomolecular condensates. **a** FRAP is utilized to investigate the dynamics of phase-separated condensates within cells. In this process, a laser is used to bleach a specific region, leading to a reduction in fluorescence. The recovery of fluorescence in the bleached area is then tracked over time, providing insights into the mobility and molecular exchange within and between the condensates. The left panel depicts a cell with condensates, with a laser being targeted at one of them for bleaching. The right panel presents a graph of fluorescence intensity versus time, showing a rapid decline during the bleaching event, followed by a gradual recovery. **b** The optoDroplet consists of a photoreceptor domain (PHR), a candidate region for LLPS, and a fluorescent protein (mCherry). Upon exposure to blue light at 450 nm, the PHR domain is activated, initiating phase separation and the formation of condensates within the cell. **c** The phase diagram demonstrates the conditions under which a system transitions into two distinct phases. The vertical axis represents the critical concentration necessary for phase separation into two phases, while the horizontal axis depicts time. The diagram features key regions, including the critical point, the one-phase region, and the two-phase region, which are associated with mechanisms like nucleation (binodal composition) and spinodal decomposition

Phase separation in biological systems, such as biomolecular condensates, can be studied using a variety of experimental techniques. These methods enable researchers to investigate the physical properties, dynamics, and functional roles of phase-separated compartments within cells. Recent advancements in cell-free experiments, particularly through protein purification, have enabled the testing of phase separation under diverse in vitro conditions.^15^ One key technique for studying phase separation is Fluorescence Recovery After Photobleaching (FRAP). FRAP is a technique used to study the dynamics of fluorescently labeled proteins or biomolecules within cells, providing insights into their mobility and interactions. This method can also be applied to purified proteins in vitro to assess their dynamic properties and interactions. In a typical FRAP assay, intracellular components are tagged with a fluorescent marker such as Green Fluorescence Protein (GFP). A small region containing the condensate is then subjected to intense laser-induced photobleaching, which irreversibly reduces the fluorescence in that area. Following photobleaching, fluorescence recovery is monitored using lower laser intensity. This recovery occurs as unbleached molecules diffuse into the bleached region or as proteins exchange rapidly between the condensate and the surrounding cytoplasmic pool.^16^ If recovery occurs primarily through diffusion, the time required for fluorescence recovery is proportional to the size of the bleached area, and the ratio of mobile to immobile fractions indicates the extent of molecular movement (Fig. 1a). For example, FRAP assays have revealed that hnRNPA1, an RNA-binding protein that readily forms phase-separated structures, has a recovery time of ~4.2 s, whereas an RNA helicase shows a recovery time of about 2.5 s, with an 80% recovery rate.^17,18^ Similarly, cytoplasmic GFP-fused TAZ, a transcriptional co-activator of Hippo pathway, exhibits rapid diffusion and recovery. Notably, a small GFP-TAZ condensate in the nucleus has a recovery time of ~2.8 s and a mobility fraction greater than 70%.^19^ Moreover, FRAP has proven crucial in distinguishing between different types of phase separation. For instance, when LLPS is intensified, gel-like condensates can form, revealing distinct physical properties. Recent studies have utilized FRAP to explore how FUS protein affects the properties of TAZ condensates. It was found that TAZ condensates with FUS protein exhibit characteristics of LLPS and recover fluorescence faster compared to TAZ condensates without FUS protein, which show more gel-like properties. This was confirmed by FRAP, which demonstrated that TAZ condensates with FUS protein had faster recovery times and a significantly reduced proportion of immobile fractions.^20^

OptoDroplet technology, developed by Shin et al. represents a significant advancement in studying the complex dynamics and functions of biomolecular condensates.^21^ This innovative tool was specifically designed to determine whether a particular region of a protein can trigger the formation of biomolecular condensates within a cell. The core of the OptoDroplet assay lies in the CRY2 protein from *Arabidopsis thaliana*, which oligomerizes in response to blue light (wavelengths between 380 and 500 nm). In this system, the PHR domain of the CRY2 protein is fused to a protein of interest. When exposed to a wavelength band similar to blue light, this fusion induces reversible self-oligomerization, facilitating phase separation. This allows researchers to compare variants that either lack or enhance interaction with the candidate protein, or with a reference protein known for its phase separation properties. Microscopic images of the process can be quantified to assess the propensity of a particular protein construct to form phase-separated biomolecular condensates^22,23^ (Fig. 1b). For example, when a DNA construct tags fluorescent protein to CRY2 PHR and incorporates the intrinsically disordered region (IDR; segments of proteins that lack a fixed or stable three-dimensional structure) of FUS—a region known for its role in biomolecular condensate formation—distinct outcomes are observed. In cells expressing this construct, the CRY2 PHR protein without the FUS IDR does not form condensates, whereas the construct containing the FUS IDR rapidly induces condensate formation upon exposure to blue light. The kinetics of this process are directly influenced by blue light intensity and the level of protein expression: higher light intensity and expression levels accelerate condensate formation. Moreover, a point mutant version of CRY2 (E490G), referred to as the Cry2oligo construct, exhibits greater sensitivity to blue light, resulting in faster condensate formation. By using IDR fusions with various other self-associating optogenetic proteins, researchers can precisely tune the dynamics of light-induced intracellular phase separation.^21,24–26^

Phase separation is often assessed using phase diagrams, which can be constructed experimentally to map the conditions that result in a single phase versus those that promote phase separation. These diagrams consider various physical factors such as pH, temperature, and concentration. Typically, three phases are identified: a one-phase state, where the system remains homogeneous; a nucleation-phase (or binodal composition), where the system is thermodynamically metastable and separates into two phases when certain thresholds are exceeded; and spinodal decomposition, where the condensate grows in size through coarsening, leading to the formation of specific structures like entanglement in a thermodynamically unstable state. These phase diagrams, whether derived from in vitro experiments or in vivo studies, provide valuable insights into the critical parameters driving phase separation and the conditions under which different phases coexist^27–29^ (Fig. 1c).

Collectively, these methodologies contribute significantly to our understanding of LLPS, providing valuable insights into the foundational principles that govern the assembly, dynamics, and functional significance of phase-separated compartments. This methodological advancement is pivotal for elucidating the implications of phase separation in cellular organization, function, and disease pathology. Ultimately, these insights guide the development of strategies for therapeutic intervention in conditions where abnormal phase separation processes are involved.

Biomolecular condensates are crucial for numerous physiological processes within cells. They regulate gene expression by concentrating key factors, such as MED1 (Mediator Complex Subunit 1) and OCT4, which are necessary for promoter and super-enhancer (SE) looping. This is achieved by recruiting RNA polymerase II and other transcriptional machinery via phase separation, or by sequestering transcriptional regulators to modulate their activity. Additionally, LLPS plays a role in the structural organization of chromosomes under normal conditions; for instance, phase separation of histone proteins contributes to chromatin structure, while excessive acetylation can disrupt these condensates, influencing transcriptional activity.^30–32^ Furthermore, during DNA damage responses, biomolecular condensates facilitate the recruitment or sequestration of key factors. FUS proteins, for example, use LLPS to accumulate splicing factors at damage sites,^33^ while Rad52 collaborates with DNA damage-induced intranuclear microtubule filaments to aggregate repair proteins in droplets, thereby preserving genomic stability.^34,35^ LLPS also enhances enzymatic activation by concentrating substrates and cofactors, enabling rapid responses to intracellular stress, and plays a critical role in intracellular signal transduction and amplification, processes fundamental to cell growth, differentiation, and response to external stimuli as will be discussed later in this review. However, dysregulation of biomolecular condensates and associated signaling pathways is implicated in the pathogenesis of various human diseases, such as cancer,^36^ immunological response^37^ and neurological disorders.^38^ For instance, aberrant condensate formation and signaling pathway activation are observed in cancer, where dysregulated signaling cascades drive uncontrolled cell proliferation and metastasis.^39–43^ Similarly, disruptions in biomolecular condensates and signaling pathways contribute to neurodegenerative diseases like Alzheimer’s,^44^ and Parkinson’s,^45^ where impaired cellular communication and protein aggregation lead to neuronal dysfunction and degeneration.^46^ Understanding the cross-talk between biomolecular condensates, signaling pathways, and cellular physiology is essential for unraveling disease mechanisms and developing targeted therapeutic interventions. By exploring the molecular mechanisms underlying condensate formation and signaling pathway regulation, we can identify novel drug targets and therapeutic strategies across a range of diseases characterized by dysregulated cellular signaling and biomolecular condensates.

## Regulatory mechanisms of biomolecular condensates

### Role of multivalent interactions in condensate assembly

**Fig. 2:**
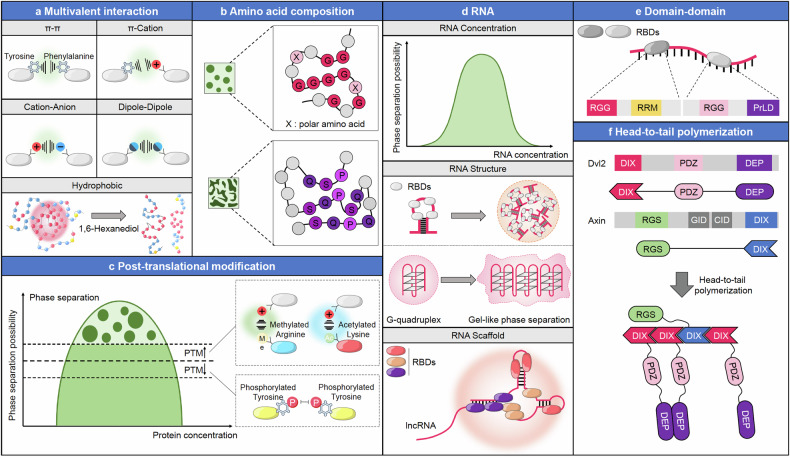
Mechanisms and factors influencing biomolecular condensates. Diverse molecular mechanisms and factors regulate the formation and modulation of biomolecular condensates. **a** Multivalent interaction: Various non-covalent interactions contribute to phase separation in cells. These include π–π interactions (e.g., tyrosine-phenylalanine interactions), π–cation interactions, cation-anion interactions, dipole-dipole interactions, and hydrophobic interactions. The lower panel illustrates the role of 1,6-hexanediol in disrupting hydrophobic interactions, leading to the dissolution of phase-separated condensates. **b** Amino acid composition: The propensity for phase separation can be influenced by the composition of specific amino acids. Sequences rich in glycine (G) and glutamine (Q) promote phase separation due to their unique physical properties, while other residues may act as spacers or modifiers within these domains. **c** Post-translational modification (PTM): PTMs, such as methylation and phosphorylation, alter the phase separation potential of proteins. The left graph illustrates the relationship between protein concentration and phase separation, with PTMs either promoting or inhibiting this process. The inset diagrams show examples of modifications: methylated arginine and acetylated lysine enhance multivalent interactions, thereby promoting phase separation, while phosphorylated residues repel each other, reducing the likelihood of phase separation. **d** RNA: RNA concentration and structure are critical in driving phase separation. The upper panel illustrates that there is an optimal RNA concentration for inducing phase separation, as depicted by the bell-shaped curve. The middle panel emphasizes how specific RNA structures, such as G-quadruplexes, can promote gel-like phase separation, serving as scaffolds that facilitate the formation and stabilization of biomolecular condensates. The bottom panel shows how RNA serves as a central scaffold in organizing and assembling biomolecular condensates. RNA-binding proteins bind to RNA molecules, forming a multivalent interaction network that enhances phase separation. **e** Domain-Domain Interactions: Phase separation can be driven by interactions between specific protein domains. For example, RNA-binding proteins with domains such as RGG, RRM, and PrLD can interact with each other to facilitate phase separation. **f** Head-to-Tail Polymerization: Certain proteins undergo head-to-tail polymerization, a process that promotes the formation of phase-separated structures. The diagram illustrates examples involving Dvl and Axin, where specific domain interactions lead to the assembly of large multivalent complexes, enhancing phase separation within the cell

Multivalent interactions, which drive phase separation and create concentrated microenvironments within cells, are driven by various forces such as π-π interactions, cation-anion interactions, dipole-dipole interactions, hydrophobic interactions, and electrostatic interactions.^47–51^ These biomolecular condensates, formed through such interactions, can be disrupted and degraded using agents like 1,6-Hexanediol^29^ (Fig. 2a). A key contributor to these multivalent interactions is the presence of IDRs within biomolecules, as exemplified by the FUS-IDR described in the OptoDroplet assay. Unlike structured domains, IDRs are highly flexible and dynamic, enabling them to engage in multiple, often transient and weak, interactions with various binding partners. This inherent multivalency allows IDRs in nucleic acids, proteins, and molecules to drive phase separation by clustering molecules into dense, concentrated phases, ultimately leading to the formation of biomolecular condensates.^52^

#### Amino acid composition-mediated condensate assembly

Studies have demonstrated that the composition of amino acids significantly is involved in the regulation of phase separation. The “stickers and spacers” model provides insight into how different amino acid residues contribute to the formation of biomolecular condensates. According to this model, “stickers,” such as aromatic amino acids like phenylalanine (Phe) and tyrosine, are crucial for driving multivalent interactions These interactions facilitate the clustering of molecules into condensed phases. In contrast, “spacers,” including glycine (Gly) and polar amino acids, enhance the liquid-like and fluid properties of phase-separated states by providing flexibility and spacing between the “stickers”.^53–55^ Additionally, proteins that are rich in glutamic acid (Glu), serine (Ser), and proline (Pro) tend to form more gel-like or solid-like condensates.^56,57^ Thus, changes in the amino acid composition can disrupt phase separation or alter its physical properties^58,59^ (Fig. 2b). While these interactions are not the only drivers of phase separation, multivalent and sequence-specific interactions are essential for local phase separation events.^60^ Overall, the amino acid composition of proteins is crucial for their interactions with other proteins, nucleic acids, and biopolymers, thereby modulating both the formation and properties of phase-separated structures within cells.

#### PTM-mediated condensate assembly

Post-translational modifications (PTMs) are covalent alterations to the backbone or side chains of proteins that greatly enhance their structural and functional diversity. These modifications affect a protein’s physicochemical properties, spatial organization, stability, cellular localization, and interactions with other biomolecules, such as proteins and nucleic acids.^61–63^ PTMs, such as phosphorylation, acetylation, and ubiquitination, regulate the formation and dissolution of biomolecular condensates through LLPS. By enabling the compartmentalization of cellular processes, PTMs allow cells to efficiently respond to external stimuli while minimizing energy expenditure. LLPS, a form of cellular compartmentalization, is closely linked to PTMs. These modifications alter the biochemical properties of amino acid residues, affecting biomolecule size and modulating intermolecular multivalent interactions.^64^ IDRs, which are central to LLPS, are particularly sensitive to PTMs. Aberrant PTMs can disrupt normal phase separation, resulting in pathological condensates associated with various diseases. A prominent example is the tau protein, which, when excessively phosphorylated or ubiquitinated, forms abnormal aggregates linked to neurodegenerative disorders. Phosphorylation, a common PTM, has bidirectional effects on phase separation.^64^ The addition of a phosphate group introduces a negative charge, which can enhance both electrostatic attraction and repulsion, thereby either promoting or inhibiting phase separation.^65–67^ Similarly, methylation can have dual effects on LLPS: it can either destabilize and reduce phase separation^18,68–73^ or promote it.^74^ Conversely, LLPS can also influence PTMs. For instance, phase separation can activate ubiquitin ligase substrates, which are crucial for ubiquitin-dependent protein homeostasis, leading to ubiquitination and subsequent proteasomal degradation^75–77^ (Fig. 2c). Thus, PTMs not only regulate LLPS but also fine-tune signal transduction pathways by modulating protein interactions and activities within condensates. This mutual interplay ensures that cellular processes are compartmentalized and controlled efficiently. A comprehensive understanding of how PTMs and LLPS dynamically regulate each other is crucial for maintaining cellular homeostasis and function. Insights into this relationship not only deepen our understanding of cellular processes but also highlight potential therapeutic targets for diseases associated with dysregulated phase separation and PTMs.^78^

#### RNA-mediated condensate assembly

RNA has recently emerged as a promising regulator of phase separation due to its flexible and variable nature. Single-stranded RNA, with its exposed bases and negatively charged phosphate groups, binds through hydrogen bonds and electrostatic interactions with positively charged biomolecules, driving the formation of biomolecular condensates via multivalent interactions. In addition to these properties, RNA concentration critically influences condensate behavior. Moderate RNA levels enhance condensate formation by promoting these multivalent interactions. Conversely, at higher concentrations, the increased repulsion between negatively charged RNA molecules can disrupt these interactions, leading to the disassembly of the condensates^50,79,80^ (Fig. 2d, upper panel). For instance, high RNA concentrations can inhibit the phase separation of FUS, while lower concentrations of RNA actually facilitate it.^81^ Coding RNA can adopt complex structures such as hairpin loops, helix motifs, and G-quadruplexes. These secondary and tertiary structures serve as building blocks that facilitate the formation of biomolecular condensates by providing flexible binding sites for various molecules.^82–84^ Notably, these structures enable a single RNA molecule to interact with multiple RNA molecules, offering protection against ribonucleases^85^ (Fig. 2d, middle panel). Longer RNA molecules, such as long non-coding RNAs (lncRNAs), also play a significant role as scaffolds.^86^ Unlike coding RNAs, lncRNAs, which are often over 200 nucleotides in length, do not participate in transcription and translation but instead provide multiple binding sites for various molecules, thereby stabilizing biomolecular condensates^84,87^ (Fig. 2d, bottom panel). For example, lncRNA NEAT1 recruits intracellular proteins NONO and SFPQ to form a paraspeckle, a membrane-less organelle.^88^ Similarly, lncRNA Xist, with its repeated sequences, acts as a scaffold to recruit X-chromosome inactivation proteins, forming a condensate involved in X-chromosome-associated gene silencing.^89^

#### Domain-domain interaction-mediated condensate assembly

RNA-binding proteins (RBPs) are crucial in various aspects of RNA metabolism, including transcription, RNA processing, transport, and stabilization.^90,91^ Many RBPs are important participants in the formation and maintenance of liquid-like, membrane-less organelles. These proteins often contain RNA-binding domains (RBDs) and disordered, prion-like domains (PrLDs), which are involved in forming biomolecular condensates via weak multivalent interactions.^92–95^ Proteins containing arginine-glycine-rich motifs (RG/RGG) within their RBDs are known to interact with RNA and contribute to condensate formation.^96,97^ The RNA recognition motifs (RRMs), which are a common type of folded domain within RBDs, are characterized by their charged residues that enable RNA binding. These RRMs not only interact with RNA but can also mediate phase separation by engaging in intermolecular interactions via their charged surfaces.^84,98^ These interactions are crucial for the formation and stabilization of membrane-less organelles, such as stress granules and processing bodies, which play key roles in cellular responses to stress and in the regulation of gene expression^17,99,100^ (Fig. 2e).

#### Head-to-tail polymerization in higher-order assemblies and condensate formations

Head-to-tail polymerization is a critical process by which specific protein domains form linear filaments that can further organize into higher-order assemblies. This process involves the sequential end-to-end (head-to-tail) interaction of protein domains, leading to the formation of polymers that can be cross-linked to create more complex structures. These higher-order assemblies are often integral to the formation and stabilization of biomolecular condensates. Several protein domains are known to drive head-to-tail polymerization, including the DIX domain, PB1 domain, and SAM domain. These domains polymerize through both homo- and hetero-oligomeric interactions, contributing to the structural organization within cells. For example, the DIX domain, found in proteins such as Disheveled and,^101–103^ plays a crucial role in the Wnt/β-catenin signaling pathway. Here, DIX domain-mediated polymerization facilitates the phase separation necessary for the formation of the Wnt signalosome, a biomolecular condensate that is essential for effective Wnt signaling during cellular development and homeostasis (Fig. 2f). Structurally similar to the DIX domain, the PB1 domain is composed of four beta-strands and one alpha-helix, forming a ubiquitin-like fold. This domain is present in proteins such as Par-6, MEK kinase, p62, and NBR1.^104,105^ The PB1 domain of p62 undergoes head-to-tail polymerization through homotypic interactions, resulting in the formation of structurally stable and dynamic condensates. These condensates are essential for various cellular functions, including selective autophagy and signal transduction, as they provide a platform for the clustering and concentration of signaling molecules. In contrast, the SAM domain, which is structurally distinct from the PB1 and DIX domains, is composed of a helical bundle made up of five alpha-helices. Despite its different architecture, the SAM domain also polymerizes through head-to-tail interactions to form oligomeric filaments. Due to the high binding affinity of SAM domains, these filaments can assemble into larger and more stable supercomplexes. Proteins associated with SAM domains include Tankyrase and Polycomb. Tankyrase, which plays a critical role in the Wnt signaling pathway, uses its SAM domain to form head-to-tail filaments that create dynamic puncta. These puncta facilitate the degradation of Axin, a negative regulator of the Wnt pathway, thereby modulating signal transduction.^106^ On the other hand, Polycomb protein self-polymerizes through its SAM domain to form dynamic nuclear puncta. These puncta play a crucial role in forming repressive chromatin networks, mediating transcriptional repression, and regulating gene expression.^107–109^ Overall, the polymerization of DIX, PB1, and SAM domains not only supports the formation of higher-order assemblies but also enables the concentration and clustering of signaling molecules at specific cellular sites. This mechanism amplifies weak signals and orchestrates complex physiological processes, including signal transduction, transcriptional regulation, and RNA processing, ensuring precise cellular responses to various stimuli.^110^

#### Structural diversity and dynamics of higher-order assemblies

Recent research has expanded our understanding of the structural diversity found in higher-order assemblies, which arise through interactions among folded domains, linear motifs, and IDRs. These assemblies, unlike traditional protein complexes, are formed through processes such as polymerization or cross-linking, resulting in structures that vary in size, shape, and dynamic behavior. The specific mechanisms of their formation, as well as the regulatory factors involved, greatly influence the structural and functional properties of these complexes. Examples of such higher-order assemblies include amyloids, prions, signalosomes, nuclear granules, and cytoplasmic granules. Amyloids and prions are fibrous protein complexes primarily assembled through interactions involving IDRs, with a core stabilized by beta-sheet structures. These complexes are typically static and irreversible, stabilized by solvent-excluded, self-complementing steric zipper interactions.^111,112^ An example of this type of assembly is the necrosome, which includes RIP-1 and RIP-3 kinases and plays a pivotal role in necrotic cell death signaling.^113^ Signalosomes are dynamic signal transduction complexes that can form through different mechanisms, such as head-to-tail polymerization and the assembly of Death-fold domains.^114,115^ Death-fold domains, characterized by a six-helix bundle structure, can lead to homo- and hetero-oligomer complexes that, like amyloids, can be static when formed through helical polymerization. Signalosomes often consist of multiple similar domains connected by IDRs, which form helical filaments and lead to the formation of biomolecular condensates. These condensates create transient clusters of signaling molecules, enhancing local concentrations and enabling rapid cellular responses to activation signals. The dynamic nature of signalosomes is further exemplified by structures formed via multivalent crosslinking and interactions among IDRs. These loosely packed assemblies are highly reversible, allowing for rapid disassembly and reformation in response to cellular cues.^115^ A prime example is the formation of liquid droplets through LLPS, as seen in T-cell receptor (TCR) microclusters. These microclusters are maintained by the crosslinking between phosphotyrosine motifs of SH2 domains and PRMs of SH3 domains during TCR activation.^116^ Ribonucleoprotein (RNP) granules and nuclear pore complexes (NPCs) also exemplify higher-order assemblies formed through multivalent actions of IDRs. RNP granules, such as nucleoli involved in ribosome biosynthesis, as well as Cajal bodies, P-bodies, and stress granules involved in RNA processing, are liquid droplets that serve as hubs for RNA-related processes within the cell.^11,84,117,118^ In NPCs, FG-repeat nucleoporins undergo phase separation to form a hydrogel, which functions as a selective permeability barrier for nuclear transport receptors.^119–122^ Both RNP granules and NPCs rely on repetitive short motifs to create reversible, dynamic structures through protein-protein interactions, allowing them to efficiently regulate various cellular processes in response to environmental changes.^115^

### Regulation of biomolecular condensates: insights into physicochemical factors

Biomolecular condensates within cells are dynamic structures whose formation, maintenance, and disassembly are governed by various physicochemical factors, including temperature, pressure, pH, and salt concentration. These factors influence phase separation, a fundamental thermodynamic process that underlies the formation of biomolecular condensates. As a result, the physical properties and behavior of these condensates are highly sensitive to changes in their external environment. Understanding how these physicochemical factors influence biomolecular condensates provides critical insights into their behavior and stability. This knowledge is fundamental for developing targeted therapies and drugs aimed at modulating condensate dynamics in various disease contexts.^19,123,124^

#### Concentration-dependent condensates assembly

The assembly of biomolecular condensates is profoundly influenced by macromolecular concentration and crowding effects. Within the cellular environment, numerous complex molecules are present, and phase separation provides a mechanism for these molecules to concentrate into distinct phases. This concentration-driven process results in the formation of localized aggregates, as molecules become denser within confined spaces. As the density of macromolecules increases, particularly those with IDRs, they come into closer proximity, facilitating binding and phase separation through weak multivalent interactions. An increase in the concentration of phase-separating molecules can push the system beyond a critical threshold necessary for phase separation, making it more likely to occur even under less favorable conditions. For example, in *C. elegans* embryos, nuclei are assembled only when the concentration of specific nuclear components exceeds a certain threshold, highlighting how concentration impacts phase separation.^125,126^ Additionally, research has shown that higher protein concentrations are associated with more rapid formation and larger sizes of condensates, both in vivo and in vitro.^127–129^ In particular, changes in protein concentration, driven by factors such as transcriptional surges or the release of proteins from storage, can profoundly affect phase separation. In some diseases, overexpression of proteins can lead to the recruitment of pathogenic factors through phase separation, shifting the nature of the condensates from a liquid-like state to a more solid-like state. This transition can exacerbate disease progression by forming more rigid aggregates.^130,131^

#### Temperature-dependent condensates assembly

Temperature significantly influences the formation and dissolution of biomolecular condensates, affecting their structure and dynamics. At lower temperatures, reduced molecular motion results in a more rigid and stable condensed state, enhancing structural stability but potentially limiting functional flexibility. Conversely, higher temperatures increase molecular motion, making condensates more fluid and dynamic. However, if temperatures become excessively high, proteins may denature, disrupting condensate formation. Temperature affects phase separation by modulating the interaction energy between proteins. Recent research indicates that hydrophobic interactions, which are crucial for condensate formation, generally strengthen with increasing temperature up to a certain point. Beyond this optimal temperature, further increases can weaken these interactions, thereby inhibiting phase separation. For instance, proteins such as hnRNP A1^132^ and FUS45^133^ exhibit phase separation when the temperature is reduced below a critical threshold. Conversely, proteins like poly(A)-binding protein in stress granules,^124^ the nucleocapsid protein of SARS-CoV-2,^134^ and ELF3^135^ from *Arabidopsis thaliana* undergo phase separation when temperatures are elevated above a critical point.^136,137^ Overall, temperature acts as a key regulator of biomolecular condensates by influencing protein interactions and phase separation dynamics. The precise temperature conditions can thus dictate the formation, stability, and functionality of these condensates in various biological contexts.

#### pH-dependent condensates assembly

pH and ionic strength are critical determinants of biomolecular condensate formation and stability, significantly influencing protein interactions and solubility. Changes in pH modify the charge state of amino acid residues, thereby affecting electrostatic interactions and hydrogen bonding within proteins. These alterations can lead to substantial shifts in the protein’s tertiary structure and its tendency to undergo phase separation.^138^ At different pH levels, the ionization state of amino acid side chains shifts, affecting their ability to form electrostatic interactions and hydrogen bonds. This can lead to changes in the protein’s tertiary structure and its propensity to undergo phase separation. Proteins may aggregate or dissolve depending on their relationship with the pH and their isoelectric point. Near their isoelectric point, proteins exhibit reduced solubility and increased propensity for aggregation. Conversely, deviations from this pH can either enhance solubility or promote phase separation, depending on the specific pH conditions.^139–141^ Ionic strength also plays a crucial role in condensate assembly by affecting electrical interactions between proteins. Higher ionic strength can mitigate electrostatic repulsion through electrical shielding, thereby facilitating stronger protein-protein interactions and promoting condensate formation. In contrast, low ionic strength can increase repulsion between charged residues, which may inhibit condensate formation.^142^ In summary, both pH and ionic strength are essential in regulating the formation, stability, and functionality of biomolecular condensates by modulating protein charge states, interaction dynamics, and solubility.

#### Pressure-mediated condensate assembly

Pressure plays a significant role in modulating the formation and stability of biomolecular condensates by influencing intermolecular distances and densities. High pressure compresses intermolecular spaces, which enhances phase separation and increases the density of condensates. This effect arises because the reduced distance between molecules facilitates their interactions, promoting the formation of a more concentrated and stable condensed phase. Conversely, low pressure increases intermolecular distances, thereby inhibiting phase separation and reducing condensate formation.^143,144^ Osmotic pressure is another critical factor affecting condensate dynamics. High osmotic pressure, typically resulting from the removal of water from the cellular environment, leads to increased molecular concentration. This concentration shift favors condensate formation by enhancing interactions among the molecules. On the other hand, low osmotic pressure, caused by water influx into the cell, decreases molecular concentration and can drive condensates to dissolve as the system becomes less favorable for their maintenance. These pressure-induced changes in molecular interactions—whether through direct effects of mechanical pressure or osmotic variations—highlight the sensitivity of biomolecular condensates to physical stimuli.^145,146^ Understanding these pressure-mediated effects provides valuable insights into how external conditions influence condensate behavior and stability. This knowledge is fundamental for developing therapeutic strategies and drugs aimed at targeting disease-related biomolecular condensates and their associated pathologies.^29,147^

## Signaling pathways involving biomolecular condensates and its role in human health and diseases

### Phase separation of Hippo pathway

#### Brief overview of Hippo pathway

**Fig. 3:**
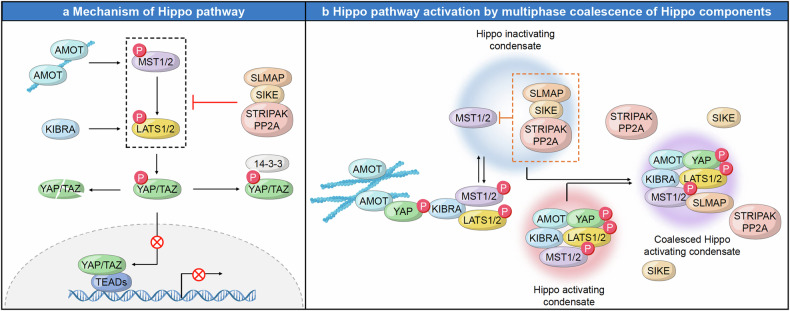
Mechanism and activation of the Hippo pathway through phase separation. Dynamic regulation of the Hippo pathway through phosphorylation events and the assembly of either activating or inhibiting condensates via phase separation, demonstrating the critical balance required for proper pathway function. **a** When the Hippo signaling pathway is active, MST1/2 initiates the activation of LATS1/2 by triggering phosphorylation events. AMOT (Angiomotin) and KIBRA also play roles in inactivating YAP/TAZ via phosphorylating MST1/2 and LATS1/2, respectively. The phosphorylated LATS1/2 kinases inhibit YAP/TAZ by phosphorylating them and controlling their subcellular localization. Phosphorylated YAP/TAZ is sequestered in the cytoplasm by binding to 14-3-3, and eventually undergoes proteasomal degradation. Conversely, when Hippo signaling is deactivated, the STRIPAK complex regulates MST1/2 by phosphorylation, which suppresses the phosphorylation cascade, allowing YAP/TAZ to translocate to the nucleus. Once inside the nucleus, YAP/TAZ form complexes with TEAD transcription factors to activate the expression of their target genes. **b** Under normal conditions, the SLMAP-SIKE-STRIPAK complex assembles into a condensate that inactivates the Hippo pathway by preventing MST1/2 phosphorylation. However, during Hippo pathway activation, AMOT and KIBRA form condensates with MST1/2, LATS1/2, and YAP. When these activating condensates form, they inhibit the SLMAP-SIKE-STRIPAK condensate and lead to the generation of Hippo-activating condensates containing AMOT, KIBRA, and MST1/2. These coalesced condensates facilitate the phosphorylation of YAP/TAZ, thereby promoting Hippo pathway activation

The Hippo pathway is a complex and tightly regulated signaling network that integrates various cellular signals to control fundamental aspects of cell behavior, including growth and apoptosis. The reciprocal communications between different regulators and components within this pathway warrant precise control over cellular outcomes, maintaining tissue homeostasis and preventing unchecked cell proliferation, eventually orchestrating organ size determination and maintaining tissue homeostasis.^148–153^ At its core lie key kinase cascade elements controlling the activity of the transcriptional co-activators YAP (Yes-associated protein) and TAZ (transcriptional co-activator with PDZ-binding motif) via phosphorylation-dependent proteolysis.^154–158^ Comprised of Hippo pathway components such as the Ste-20 family protein kinases MST1/2, the scaffold protein Salvador, the MOB kinase activator 1A/B (Mob1A/B), and the large tumor suppressor kinases LATS1/2, the Hippo pathway is finely attuned to respond to various signals.^159–163^ These cues, ranging from cell crowding to polarity, energy status, osmotic stress, and mechanical inputs, coordinately modulate the activity of Hippo signaling pathway. Upon stimulation by diverse environmental cues, MST1/2 kinases, in conjunction with Salvador, catalyze the activation of LATS1/2 kinases. Subsequently, activated LATS1/2 phosphorylate YAP and TAZ, leading to their cytoplasmic and eventual degradation mediated by the ubiquitin-proteasome system. This sequestration prevents YAP and TAZ from translocating to the nucleus, where they interact with TEA domain family members (TEADs) to activate the transcriptional program that drives cell proliferation and survival.^164–166^ Critical to the precise orchestration of the Hippo pathway is a center of junctional regulators. These include pivotal junctional proteins like angiomotin (AMOT), neurofibromin 2 (NF2), and KIBRA, which facilitate the transmission of signals enhancing MST1/2 and LATS1/2 kinase activities. These activations, particularly in response to cell density, result in the inhibition of cell growth by cell-cell contact inhibition.^167–171^ In contrast, the Hippo pathway can be negatively regulated through the sarcolemmal membrane-associated protein (SLMAP) complex, a multi-subunit assembly encompassing the phosphatase catalytic subunit PP2A, regulatory subunits striatins (STRNs), STRN-interacting proteins (STRIP1/2), striatin-interacting phosphatase and kinase (STRIPAK), and a suppressor of IKKs (SIKE).^172–175^ This complex antagonizes the pathway by dephosphorylating and thus deactivating MST1/2 kinases, thereby dampening Hippo pathway activity. Moreover, intrinsic or extrinsic stimuli, such as cytoskeletal rearrangements, genetic variations, or growth factors, can impede LATS1/2-mediated YAP/TAZ phosphorylation. This impediment prompts the nuclear translocation of YAP and TAZ, where they interact with TEADs, culminating in the activation of a transcriptional program essential for cell proliferation and survival^157,158^ (Fig. 3a). As mentioned above, the Hippo signaling pathway is regulated by a multitude of factors that influence its activity and downstream effects on cellular behavior. A recently emerging mechanism that has gained attention in recent years is LLPS. In the context of Hippo signaling, LLPS plays a role in organizing the spatial distribution and concentration of pathway components, thereby influencing their interactions and activity.

Overall, the physiological connection between Hippo signaling regulation and LLPS highlights the complex nature of cellular signaling networks. Understanding how LLPS influences Hippo pathway dynamics provides new insights into the mechanisms underlying tissue development, homeostasis, and diseases. We discuss how the functions of core components in the Hippo pathway is regulated by LLPS, focusing specifically on their implications in pathophysiological processes.

#### Hippo pathway activation by multiphase coalescence of Hippo components

The activation of the Hippo pathway is regulated by the multiphase coalescence of its components, orchestrated by various upstream regulators. These regulators include AMOTs (KIBRAs, NF2, PTPN14, and the SLMAP complex), which play crucial roles in modulating Hippo signaling in response to cellular cues such as cell contact inhibition, polarity, or osmotic stress.^153,176^ Specifically, AMOT, KIBRA, and NF2 act as positive regulators, while the SLMAP complex exhibits contrasting effects by promoting the dephosphorylation of MST1/2, leading to increased nuclear localization of YAP/TAZ. Recent studies have unveiled that the Hippo pathway’s upstream regulators undergo LLPS. The SLMAP complex, for instance, forms biomolecular condensates that deactivate the Hippo pathway, whereas AMOT and KIBRA form Hippo-activating condensates. These condensates facilitate the phosphorylation of key components in the MST-LATS-YAP core kinase cascade, ultimately activating Hippo signaling. Interestingly, AMOT and KIBRA can coalesce to form bi-scaffold condensates, which further interact with the SLMAP condensate to form a triscaffold condensate. This AMOT/KIBRA/SLMAP triscaffold condensate enhances Hippo signaling by inhibiting the function of Hippo-inactivating SLMAP condensates. The formation of this triscaffold condensate is dependent on AMOT, as it strengthens the interaction between KIBRA and SLMAP. Moreover, the competitive binding of SIKE and KIBRA to SLMAP further modulates the function of SLMAP condensates. By excluding SIKE from the SLMAP condensates, the AMOT/KIBRA/SLMAP triscaffold condensate effectively inhibits SLMAP function, thereby enhancing Hippo signaling. Overall, the coalescence of Hippo pathway components into biomolecular condensates, regulated by various upstream regulators, represents a novel mechanism underlying Hippo pathway regulation^177^ (Fig. 3b).

#### Phase separation of the Hippo pathway in cancer

**Fig. 4:**
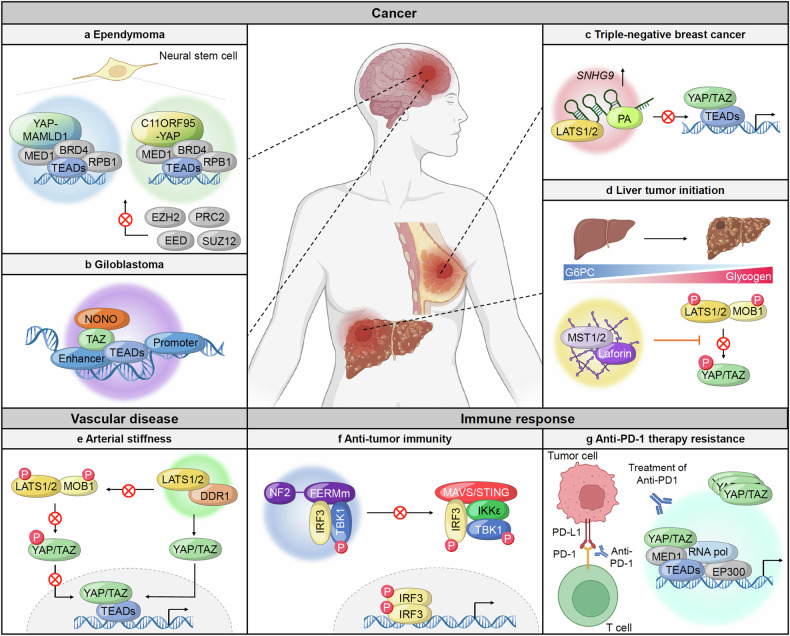
Pathogenesis and therapeutic resistance mediated by the phase separation of the Hippo pathway. **a** YAP-MAMLD1 and C11ORF95-YAP fusion proteins in neural stem cells undergo phase separation with various transcription factors, including chromatin remodeling factors. This sequestration from the PRC2 complex promotes ependymoma development by disrupting normal transcriptional regulation. **b** The paraspeckle protein NONO promotes the phase separation of TAZ, activating TAZ-driven oncogenic transcriptional programming in glioma cells. **c** Elevated expression of the lncRNA SNHG9 in triple-negative breast cancers recruits LATS and PA to form phase-separated condensates. **d** In the early stages of liver cancer, downregulation of the G6PC gene leads to glycogen accumulation. This accumulation forms a phase separation with MST1 and Laforin, activating the Hippo pathway and promoting tumor initiation. **e** In arterial stiffness, LATS1/2 interacts with DDR1 to form condensates. This interaction prevents YAP phosphorylation, allowing unphosphorylated YAP to translocate to the nucleus and associate with TEAD, leading to the activation of target gene expression. **f** Mutations in the FERM domain of NF2 lead to condensate formation with IRF3 and TBK1, inhibiting MAVS/STING signaling. This inhibition results in IRF3 phosphorylation, which induces an antitumor immune response. **g** In cancer cells undergoing anti-PD-1 therapy, elevated YAP expression results in the formation of condensates with transcriptional cofactors TEAD, RNA polymerase, and EP300. These condensates drive the expression of target genes such as Myc and CD155, contributing to resistance against anti-PD-1 therapy

YAP, a pivotal transcription factor within the Hippo signaling cascade, has emerged as a central player in phase separation dynamics, particularly notable under conditions of hyperosmotic stress. Under such conditions, YAP undergoes rapid condensation, giving rise to distinct cytoplasmic and nuclear condensates. These condensates serve as central hubs for the recruitment of diverse signaling effectors, initiating a complex reorganization of genomic landscapes. In the cytoplasm, YAP condensates act as recruitment platforms for kinases such as LATS and NLK, facilitating complicated regulatory processes.^127,178^ Conversely, nuclear YAP condensates serve as docking sites for critical transcriptional partners like TAZ and TEAD1. Notably, under hyperosmotic stress, YAP-driven nuclear condensates exhibit an enrichment of accessible chromatin regions, accompanied by the recruitment of TEAD1 and TAZ. The spatial rearrangement of chromatin orchestrated by YAP phase separation profoundly impacts transcriptional regulation. By creating and compartmentalizing specialized environments within the nucleus, YAP condensates modulate the accessibility of genomic regions, thereby influencing the expression of target genes involved in cellular processes. The dysregulation of YAP phase separation dynamics has been associated with significant implications in various pathological conditions, particularly cancer. Aberrant YAP condensation can disrupt normal signaling cascades, leading to the activation of oncogenic transcriptional programs. This disruption contributes to tumor progression by altering cellular behaviors and promoting malignant.^179,180^ Moreover, a plethora of fusion proteins arises from gene fusion events, a phenomenon linked to the onset and progression of various cancers and disorders. YAP genes frequently engage in interactions with other genes, resulting in the formation of fusion proteins that wield considerable oncogenic potential across diverse malignancies.^181^ Ependymal tumors (EPNs), primarily affecting children and developing in the ventral stream of the brain and spinal cord, exemplify pathologies where YAP fusion proteins play pivotal roles as oncogenic drivers. In individuals with EPN, fusion events involving the YAP gene and mastermind-like domain-containing protein (MAMLD1), as well as C11ORF95, have been recently identified.^182–185^ These fusion proteins, such as YAP-MAMLD1 and C11ORF95-YAP, undergo phase separation and inhibit the activity of polycomb repressive complex 2 (PRC2) in an IDR-dependent manner. Consequently, these fusion proteins prevent the repressive function of PRC2 complex within nuclear condensates, thereby activating an oncogenic transcriptional program that contribute to tumor progression. Studies suggest that YAP regulates transcriptional activity selectively through phase separation, impacting various oncogenic programs. Studies investigating the YAP-MAML2 fusion protein have shown that apart from specific Hippo-target genes such as CYR61, CTGF, and ANKRD1, the YAP-MAML2 condensate displays a distinct transcriptional program compared to regular YAP activity. This finding indicates that phase separation mechanisms may enable more precise intracellular signaling and communication^186^ (Fig. 4a).

TAZ functions as a nuclear effector within the Hippo pathway, sharing similar protein sequences, regulatory mechanisms, and functions with YAP. Like YAP, TAZ also undergoes phase separation both in vitro and in vivo under various stress conditions. Interestingly, TAZ exhibits a more flexible phase separation capability compared to YAP, forming condensates at lower thresholds. This dynamic nature of TAZ phase separation is tightly regulated under conditions influencing Hippo signaling, often co-localizing with transcriptional regulators such as TEAD4, BRD4, and MED1 to facilitate transcriptional activation.^19^ Recent studies have unveiled a novel regulator of TAZ phase separation with potential clinical implications. The Non-POU domain-containing octamer-binding protein (NONO) is frequently upregulated in various human cancers, particularly glioblastoma (GBM), where its expression correlates strongly with disease development, aggressiveness, and patient survival.^187,188^ Wei et al. identified NONO as a critical TAZ-interacting protein in the nucleus, essential for TAZ activation by enhancing accessibility to transcriptional enhancers. Moreover, the interaction between NONO and TAZ not only facilitates TAZ nuclear phase separation but also contributes to GBM oncogenicity.^189^ Expanding our understanding of TAZ nuclear condensates dynamics, Shao et al. employed a novel L-NHSF-mediated method utilizing cross-linking mass spectrometry to identify interacting proteins within protein condensates. Through this approach, they discovered that the RNA-binding protein FUS serves as a crucial regulator of TAZ fluidity and function, acting as a chaperone. In the absence of FUS, TAZ phase separation transitions towards a more solid-like state, resulting in reduced transcriptional activity and subsequent downregulation of target gene expression, ultimately hindering tumor progression^20^ (Fig. 4b).

Multiple regulatory layers precisely control the activity and stability of LATS1 and LATS2, which are pivotal and direct kinases to phosphorylate and subsequently inhibit YAP/TAZ. Through this inhibition, LATS1/2 performs a crucial tumor-suppressive function. When LATS1/2 are aberrantly repressed or mutated, their ability to inhibit YAP/TAZ is compromised, leading to unchecked cell proliferation and tumor growth. This dysregulation has been implicated in the malignant transformation of cells, contributing significantly to cancer progression.^190–192^ LncRNAs have emerged as key regulators of various signaling pathways, including the Hippo pathway, due to their diverse roles in cellular processes based on their subcellular localization. LncRNAs influence a range of cellular functions and are critical players in cancer progression.^193,194^ One such lncRNA, small nucleolar RNA host gene 9 (*SNHG9*), has been identified as substantially upregulated in several cancers, including triple-negative breast cancer (TNBC). Elegant and combined experiments with cell culture systems, in vivo tumor models, and analyses of datasets from public cancer databases has demonstrated that lncRNA *SNHG9* may be significantly associated with the development of TNBC. *SNHG9* exerts its oncogenic effects by modulating the phase separation of LATS1 with phosphatidic acid (PA), a process that suppresses LATS1 activity. By inhibiting LATS1, *SNHG9* effectively promotes YAP activity, which in turn drives tumor development and progression^195^ (Fig. 4c).

A recent report has unveiled an intriguing discovery regarding the response of LATS2 to the reduction of the F-actin cytoskeleton. It has been found that LATS2 forms a specialized molecular complex known as the proline-rich motif (PRM)-mediated LATS2 signalosome in this scenario. This signalosome serves a dual role: firstly, it physically protects LATS2 from degradation by preventing its interaction with the E3 ligase FBXL16 complex, thereby ensuring the stability of LATS2 within the cell. Secondly, the signalosome plays an active role in suppressing YAP/TAZ activities. By recruiting and inactivating YAP/TAZ, the LATS2 signalosome effectively impedes tumor progression. This inhibition of YAP/TAZ activity serves to suppress the aberrant signaling cascades associated with tumor growth and metastasis, thus exerting a tumor-suppressive effect. The mechanism underlying LATS phase separation, as observed in the formation of the LATS2 signalosome, holds significant promise as a novel diagnostic and therapeutic strategy for cancer. This phenomenon involves the dynamic organization of LATS2 into distinct liquid-like condensates in response to cellular cues. These condensates not only facilitate the sequestration of LATS2 but also enable its interaction with other cellular components, including YAP/TAZ, leading to the modulation of critical signaling pathways implicated in cancer development.^196^

MST1 and MST2 (hereafter MST1/2) function as upstream negative regulators of YAP/TAZ, inhibiting their transcriptional activity by activating the LATS1/2 serine/threonine kinase. Genetic studies and clinical analyses have demonstrated that MST1/2 are crucial for maintaining liver homeostasis and suppressing the progression and development of hepatocellular carcinoma (HCC) via YAP inactivation.^164,197,198^ Liu et al. found through analyses of liver cancer tissues from chemically treated mice, genetically engineered mouse models, and human patients that glycogen accumulation is prevalent in small, pre-malignant nodules but absent in larger tumors, suggesting its role in tumor initiation.

Notably, the accumulation of glycogen undergoes phase separation, which recruits MST1/2 and Laforin, excluding MST1/2 from the Hippo pathway. This exclusion activates YAP, leading to increased cell proliferation and tumorigenesis. Consequently, it is posited that LLPS plays a fundamental role in the regulation of Hippo-YAP signaling. This process, which involves the dynamic segregation of cellular components into distinct liquid phases (e.g., glycogen), appears to be crucial for the modulation of key signaling pathways that control cell proliferation and tumor growth. By influencing the localization and activity of critical proteins such as MST1/2 and YAP, LLPS can alter the downstream effects of the Hippo pathway, thereby impacting tumor initiation and progression. Exploring the pathophysiological implications of LLPS in greater detail promises to enhance our understanding of cancer development^199^ (Fig. 4d).

#### Phase separation of Hippo pathway in vascular disease

Arterial stiffness is a common condition observed in patients with chronic kidney disease and is closely linked to various cardiovascular risk factors including diabetes, hypertension, advancing age, and smoking, among others.^200–203^ The increased stiffness of arteries serves as a well-documented clinical indicator for the development of cardiovascular diseases and increased mortality rates. Recent findings suggest that the receptor tyrosine kinase DDR1 (Discoidin Domain Receptor 1) functions as a mechanosensor in vascular smooth muscle cells (VSMCs), playing a key role in sensing stiffness not only in VSMCs but also in diverse cell types.^204^ Mechanical signals originating from factors such as extracellular matrix (ECM) rigidity and collagen interactions are known to modulate the activity of the YAP protein.^205^ The heightened stiffness triggers DDR1 to form phase-separated condensates with LATS1, leading to the inactivation of LATS1 by disrupting its association with MOB. This DDR1-LATS1 co-condensate plays a critical role in activating YAP during the process of arterial stiffening, which is essential for YAP’s functional activation. Activation of DDR1 occurs through binding with collagen or in response to ECM stiffness, facilitating the recruitment of LATS1 to form a co-condensate via the C-terminus of DDR1^206^ (Fig. 4e).

#### Phase separation of Hippo pathway in immune response

The tumor suppressor NF2 plays a pivotal role as an upstream regulator of the Hippo pathway, which in turn modulates the activity of YAP/TAZ. Mutations in the *NF2* gene, whether germline or somatic, are the primary etiological factors for type 2 neurofibromatosis, a condition associated with a predisposition to various malignancies.^207–210^ In a recent study, Meng et al. proposed a novel pathogenic mechanism by which NF2 mitigates the inhibition of TANK-binding kinase 1 (TBK1) by reducing the function of YAP/TAZ and thereby facilitating the innate sensing of nucleic acids. However, patient-derived mutations in the *NF2* gene compromise this immunological role, rendering the NF2 protein incapable of facilitating nucleic acid sensing and inhibiting the detection of DNA/RNA through phase separation mechanisms. The NF2 protein demonstrates a remarkable ability to localize to various subcellular compartments, including the plasma membrane, cell cortex, cytoplasm, and nucleus. This localization is influenced by a variety of factors, such as physiological changes, specific mutations, or the cellular context in which NF2 operates. Intriguingly, the induction of nucleic acid sensing, followed by the activation of Interferon Regulatory Factor 3 (IRF3), leads to the dynamic condensation of NF2 mutants in the cytoplasm. These IRF3-mediated condensates of NF2 mutants enable NF2 to function as a profound suppressor of the cyclic GMP-AMP synthase-stimulator of interferon genes (cGAS-STING) signaling pathway, which is critical for nucleic acid sensing by inactivating TBK1. By disrupting the normal function of NF2, these mutations not only impair the cellular ability to detect nucleic acids but also lead to inappropriate suppression of the cGAS-STING pathway, thereby undermining the innate immune response against tumors^211^ (Fig. 4f). Cancer cells evade T cell immune responses by expressing PD-L1 ligands, which deactivate T cells through interactions with PD-1 receptors. Anti-PD-1/PD-L1 therapies, also known as immune checkpoint inhibitors, aim to block this interaction, thereby revitalizing T cell activity and restoring anti-tumor immunity.^212–214^ Despite their potential, due to various immune escape mechanisms employed by cancer cells, these treatments are effective in only about 20% of cancer patients.^215–217^ A recent study has revealed a pivotal role of YAP nuclear phase separation in the adaptive resistance to tumor immunotherapy driven by IFN-γ. Specifically, anti-PD-1 treatment has been shown to induce YAP translocation into the nucleus and the formation of punctate structures in lung adenocarcinoma cells, a phenomenon associated with immunotherapy resistance. The presence of YAP nuclear condensates in CD8^+^ T cell-infiltrated regions has enabled researchers to uncover cytokine-related signaling originating from the tumor microenvironment, identifying IFN-γ as a key factor responsible for YAP puncta formation independent of the canonical STAT1-IRF1 pathway. Domain mapping analysis has further elucidated the mechanisms behind this process, revealing that a coiled-coil (CC) region and IDR mediate YAP phase separation. This region overlaps with the previously described transactivation domain. Importantly, mutations in the leucine residues within the CC domain of YAP have been found to disrupt its phase separation capabilities. Additionally, nuclear YAP forms condensates that co-phase with various components, including TEAD4, MED1, and EP300, ensuring the effective expression of target genes. The study also demonstrated that transcription deactivation caused by mutations that disrupt YAP phase separation renders tumor cells more vulnerable to anti-PD-1 therapy^218^ (Fig. 4g). We summarized the molecular characteristics for condensates of Hippo signaling components in Table 1.

**Table 1:** Summary of molecular characteristics for condensates of Hippo pathway components

Hippo pathway components	Domain necessary for phase separation	Subcellular localization	Condensate components	Expression of YAP/TAZ dependent target genes	Function	Pathophysiological effect	Ref.
AMOT, KIBRA, SLMAP, YAP, MST1/2, LATS1/2	WW	Cytoplasm	AMOT, KIBRA, SLMAP, MST1/2, LATS1/2, YAP, SIKE, STRIPAK, PP2A	Down	-	-	^180^
YAP	TAD, CC	Nucleus, Cytoplasm	-Cytoplasm: NLK, LATS, Dcp1 -Nucleus: TEAD, TAZ, RNA pol II, nascent RAN	Up	Transcription activation hub	Anti-PD1 therapy resistance	^130,220^
YAP-MAMLD1, C11ORF95-YAP (YAP fusion protein)	IDR of fusion protein	Nucleus	MED1, BRD4, RPB1, TEAD		Driver of oncogenic programming	Ependymomas tumorigenesis	^189^
TAZ	WW, CC		NONO, TEAD4, BRD4, MED1, CDK9		Transcription activation	Glioblastoma development (GBM)	^19,192^
LATS1	PrLD	Cytoplasm	*SNHG9*, PA		Inhibiting LATS kinase activity	Breast cancer progression	^198^
	MBD		DDR1			Arterial stiffness	^209^
MST1/2	N-terminal (1-326)		Glycogen, Laforin		Sequestering MST from Hippo pathway	Liver tumor initiation (G6PC gene downregulation)	^202^
NF2	FERM domain		IRF3, TBK1	-	abolishes STING-initiated antitumor immunity in mice	-	^213^

*TAD* transactivation domain, *IDR* intrinsically disordered region, *WW* Tryptophan-Tryptophan-Proline repeating domain, *CC* coiled-coil domain, PrLD Prion-like domain, *MBD* MOB-binding domain, *G6PC* glucose-6-phosphatase catalytic subunit, *FERM domain* present in erythrocyte band 4.1, ezrin, radixin, and moesin domain

### Phase separation of cGAS-STING pathway

#### Brief overview of cGAS-STING pathway

The cGAS-STING signaling pathway plays a key role in the innate immune system, which detects double-stranded DNA (dsDNA) in the cytoplasm to elicit an immune response. In this pathway, cGAS recognizes the presence of abnormal dsDNA in the cytoplasm and produces a secondary messenger called cGAMP.^219–225^ The cGAMP binds to STING and causes a conformational change in STING, activating it.^226^ The activated STING translocates to the Golgi apparatus, where it plays a key role in activating TBK1. TBK1 phosphorylates and activates IRF3, which then translocates to the nucleus where it binds to specific gene sequences and promotes the transcription of genes important for the immune response, such as type I interferon (IFN-I).^227–232^ The cGAS-STING pathway is tightly regulated to avoid excessive immune activation or insufficient responses, and dysregulation can lead to problems such as susceptibility to infection, autoimmune disease, and cancer progression.^233–238^ Recent studies have identified biomolecular condensates as critical regulators of the cGAS-STING pathway, and modulating their formation and dynamic changes could lead to the development of novel immunomodulatory therapeutics.^239,240^

#### cGAS condensates in innate immune response

**Fig. 5:**
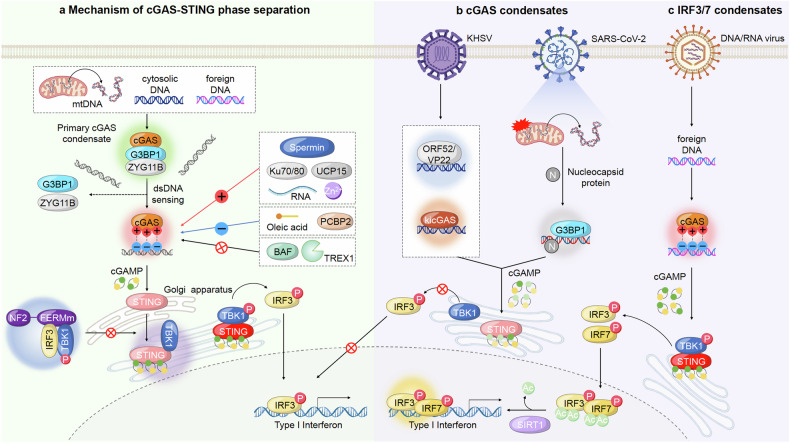
Molecular mechanisms of phase separation in cGAS-STING-mediated innate immune responses. **a** Upon sensing double-stranded DNA (dsDNA) from various sources such as mitochondrial DNA (mtDNA), cytosolic DNA, or foreign DNA, cyclic GMP-AMP synthase (cGAS) becomes activated. Activated cGAS produces cyclic GMP-AMP (cGAMP), which then binds to the stimulator of interferon genes (STING) located on the Golgi apparatus. This binding initiates the recruitment of TBK1 and leads to the phosphorylation and activation of IRF3. Phosphorylated IRF3 translocates to the nucleus to induce the expression of Type I interferon. Various factors, including RNA-binding proteins (e.g., G3BP1), oleic acid, Spermine, and proteins such as PCBP2, BAF, and TREX1, can influence the phase separation of cGAS and STING. STING condensates and NF2 mutation-mediated condensates operate to suppress the innate immune response. **b** The Kaposi’s sarcoma-associated herpesvirus (KSHV) tegument protein ORF52/VP22 competitively binds to DNA alongside cGAS, forming phase-separated condensates that evade immune detection by preventing cGAS-DNA condensate formation. During SARS-CoV-2 infection, stimulated mitochondria release mitochondrial DNA sensed by cGAS. However, the SARS-CoV-2 nucleocapsid protein sequesters cGAS, forming phase separation with G3BP1 and DNA, thereby inhibiting innate immune signaling activation. **c** Upon infection with DNA/RNA viruses, cGAS-DNA phase separation activates the cGAS-STING pathway, leading to IRF3/IRF7 activation. SIRT1 deacetylates the IRF3/IRF7 dimer to further promote innate immune signaling

The innate immune response is initiated when cGAS detects dsDNA in the cytoplasm. The basic molecular basis is that cGAS forms dimers with other nearby cGAS and binds to dsDNA through sites A, B, and C.^241^ When the four-bound activation sites are met through binding, cGAS is activated. The N-terminus of cGAS has a randomized sequence and a positive charge and has been reported to cause LLPS with negatively charged DNA.^242^ Specifically, sites A and B of cGAS work together to bind DNA and cGAS in the proper ratio to maintain a stable activation structure, and site C is an additional cGAS-DNA binding interface that promotes the formation of cGAS-DNA complexes. Indeed, siteC of cGAS promotes condensate formation through binding to DNA, and mutations in siteC led to inhibition of cGAS-dsDNA LLPS formation. Longer DNA forms condensates more efficiently with cGAS, and free zinc ions stably increase the formation of these LLPS. The resulting cGAS-dsDNA condensate efficiently recruits and enriches enzymes and the substitutes ATP and GTP, acting as a microreactor.^243^ At this point, TREX1 acts as a DNA exonuclease, cleaving the DNA to prevent interaction with cGAS. However, the formation of cGAS-dsDNA liquid condensate sequesters TREX-1 out of the droplet, protecting the DNA from cleavage, and also sequesters BAF, known as a negative regulator, to activate cGAS-STING signaling^244,245^ (Fig. 5a). For the effective generation of cGAS-dsDNA condensates, cGAS undergoes a process that allows it to react more rapidly with dsDNA through the formation of primary condensates.^246,247^ This primary condensate is mediated by G3BP1, a key assembler of stress granules that form to rapidly adapt to various intracellular stresses. G3BP1 preferentially binds directly to cGAS to form a condensate, which is then degraded by dissociation of G3BP1 and cGAS as dsDNA accumulates in the cGAS-G3BP1 condensate. At this time, zinc ions and RNA also assist in the formation of LLPS from G3BP1-cGAS.^247^ In addition, during the early stages of viral infection when these primary condensates are formed, ZYG11B also binds to cGAS along with G3BP1 to form large droplets, concentrating the relatively low concentration of cGAS in the droplets and increasing its sensitivity to dsDNA. cGAS-dsDNA secondary condensates begin to form, and ZYG11B and G3BP1 dissociate from the secondary condensates and are released into the cytoplasm. Indeed, to evade the cGAS-STING innate immune response via this mechanism, HSV-1-infected cells have been shown to respond by degrading ZYG11B via a yet unknown protein^248^ (Fig. 5a).

In addition, to promote the condensate formation of cGAS-dsDNA, Ku70/80 interacts directly with cGAS and acts as a co-sensor to increase its binding affinity to DNA, and RNA also helps to form a condensate with cGAS-dsDNA when present at a suitably low concentration and is cleaved from the condensate. It helps cGAS react more rapidly with dsDNA to form condensate effectively.^249^ USP15 cleaves the K48, K63-ubiquitination ligation chains, which induces autophagic degradation of cGAS, and has been shown to drive dimerization and liquid condensation of cGAS. In particular, the IDR of USP15 is proposed to be a dynamic regulator of cGAS condensates. The formation of these condensates activated the signaling of cGAS-STING to produce cytokines such as IFN-I, which enhanced immunity against infection and pathogen invasion.^250^ Conversely, PCBP2 inhibited the formation of cGAS-dsDNA condensate in a concentration-dependent manner by binding to the C-terminus of cGAS to which dsDNA binds,^251^ and SIRT2 inhibited the formation of cGAS-dsDNA condensate by cleaving K257, K276, and K376 residues of G3BP1, which mediated the formation of the primary condensate of cGAS, leading to the degradation of the primary condensate of cGAS-G3BP1, which impaired its DNA binding capacity, cGAS-dsDNA condensate formation, and enzymatic activation, thereby attenuating antiviral and anti-infective immune responses^252^ (Fig. 5a). Thus, positive regulation of LLPS of cGAS-dsDNA can be used to activate antiviral and innate immunity, and negative regulation of LLPS of cGAS-dsDNA can be used to treat autoimmune diseases.

#### STING phase-separator in innate immune response

The enzymatic activation of cGAS through cGAS-dsDNA activation leads to the production of cGAMP. As a secondary messenger of cGAS-STING, cGAMP binds to STING and STING undergoes a conformational change, which phosphorylates TBK1 and IRF to produce IFN. STING resides in the ER and translocates to the Golgi with the conformational change. However, excessive production of cGAMP leads to an overactive immune response, and to prevent this, in the late stages of DNA virus infection, STING forms gel-like condensates with a puzzle-like structure. This STING phase-separator traps TBK1 and interferes with the phosphorylation of IRF, which prevents its translocation to the nucleus, thereby inhibiting ISG transcriptional activation through IRF3, a phenomenon termed the STING-cGAMP-TBK1 sponge effect.^253^ Indeed, mutations occurring in the STING 153-173 region (V147L/M, N154S, V155M) can cause autoimmune diseases and a significant decrease in STING condensate and increase in STING activation has been observed in these mutants, suggesting that the mutations affect the formation of the STING phase separator and that impairment of the phase separator, which is responsible for preventing STING-TBK1 hyperactivation, can lead to autoimmune diseases. This may explain the mechanism by which these mutations cause persistent STING activation in patients. Furthermore, as previously described in the Hippo context, NF2 relieves YAP/TAZ-mediated repression of TBK1 and enhances DNA/RNA sensing. Missense mutation of NF2 (mNF2), which is a point mutation in the FERM region, is found in various cancer patients and reduces MAVS/STING-induced RNA/DNA sensing ability. Mutations in the FERM region of NF2 alter the head-to-tail structure to an open structure, resulting in a complex with TBK1 in the C-terminal tail and IRF3 in the FERM domain, which binds to form an intracellular condensate in the cytoplasm. Abundant recruitment of the RACK-PP2A complex into this condensate allows this phosphatase complex to dephosphorylate the S172 residue of TBK1 and prevent IRF3 translocation to the nucleus and transcriptional activity, acting as a negative regulator of the innate immune response. Indeed, in mNF2 mutant mouse models and patient tissues, IRF3 is also present along with mNF2 condensates, where they exert increased suppression of the innate immune response through condensate formation, imprisoning the cGAS-STING machinery to abrogate antitumor immunity^211^ (Fig. 5a).

#### Viral evasion by inhibiting cGAS-DNA phase separation

Viruses employ a variety of strategies to evade the host’s immune response by suppressing the immune system. ORF52 is an envelope protein enriched in herpesviruses that suppresses the innate immune response through viral protein-induced LLPS of DNA rather than direct interaction with cGAS. The VP22 protein, which has high homology to ORF52, also showed the same behavior. Early in viral infection, after the formation of cGAS-DNA condensates, ORF52/VP22 accumulates in cGAS-dsDNA condensates and replaces cGAS-DNA condensates as viral envelope proteins and DNA form condensates through multivalent interactions of positive and negative charges. This results in the release of ATP and GTP, substrates required for cGAS-STING signaling. In addition, the cGAS inhibitor of KHSV (hereafter kicGAS), encoded by ORF52, binds to dsDNA through self-oligomerization and forms liquid droplets upon binding to DNA, inhibiting DNA-induced phase separation and activity of cGAS.^254,255^ Infection of the SARS-CoV-2 virus induces the release of mitochondrial DNA into the cytoplasm, which activates IFN-I signaling through cGAS-STING signaling.^256,257^ However, the virus’s nucleocapsid protein competitively binds to G3BP1 with cGAS and inhibits the formation of the cGAS-G3BP1 primary condensate, reducing the DNA recognition ability of cGAS. The LLPS ability of the N protein plays a key role in this process, and among the various coronavirus variants, the N proteins of the Alpha, Beta, and Gamma variants form fast, fluid condensates that significantly weaken cGAS-DNA binding. On the other hand, the N protein of the Omicron variant forms a relatively less mobile condensate, which inhibits cGAS-DNA binding to a lesser extent, but IFN-I signaling activation tends to be transiently reduced and then recovered. Finally, the N protein of SARS-CoV-2 competitively binds to G3BP1 via LLPS, inhibiting cytoplasmic DNA recognition of cGAS and the IFN-I antiviral response. All of these interfere with the formation of cGAS-dsDNA condensates in the host, thereby sequestering key factors required for the innate immune response, thereby selecting an evasion strategy from the host’s immune response and enhancing viral proliferation.^258–260^ This strategy of weakening immune surveillance may be used not only in viral evasion but also in metabolic syndromes such as obesity. Among the fatty acids that are obesity-related metabolites, oleic acid (OA) is the most abundant fatty acid present in sera, and it has both hydrophobic and hydrophilic end groups, which means that it can disperse one liquid into another immiscible liquid, acting as a solubilizing agent in phase separation phenomena. In fact, dietary accumulated OA reversibly binds to cGAS, which leads to a decrease in cGAS-DNA binding. This suggests that obesity-associated metabolites may act to disperse the LLPS of cGAS-DNA and thus evade the innate immune response, a mechanism that weakens immune surveillance^261^(Fig. 5b).

#### Age-related factors weaken immune response in cGAS-STING phase separation

As aging progresses, the decreased expression of key factors leads to disruption of intracellular homeostasis and, in particular, attenuation of the immune response. In addition, age-related immune dysfunction not only increases susceptibility to viral infections but also contributes to increased morbidity and mortality, posing significant challenges in maintaining effective host defense mechanisms.^262,263^ Recent studies have revealed the role of phase separation in age-related immunity. Spermines are a class of polyamines present in mammals that play an important role in auto- versus non-autoimmune discrimination, and their levels are known to decrease with aging.^264^ Spermine condenses DNA and stabilizes the binding of cGAS to dsDNA, activating the immune response by activating cGAS through the cGAS-dsDNA condensate, and the decrease in spermine with aging leads to an inability to properly form cGAS condensates in age-related immunity, resulting in the phenomenon of immunosenescence.^265^ SIRT1, an NAD-dependent deacetylase known to be involved in delaying aging and regulating immune responses. Mice lacking SIRT1 displayed a complete absence of IRF3/IRF7 condensate formation and a marked impairment in antiviral immune responses. In cells deficient in SIRT1, IRF3 and IRF7 were highly acetylated at two conserved lysine residues within their DNA-binding domains (DBDs). Using a genetic code expansion system, Qin and colleagues demonstrated that acetylation at these key residues completely inhibited the formation of IRF3/IRF7 condensates and their subsequent transcriptional activation. Aligned with SIRT1’s role in aging and IRF3/IRF7 transcriptional regulation, the age-related decline in SIRT1 activity appears to exacerbate the acetylation of IRF3/IRF7 DBDs. Importantly, these processes were reversible upon restoration of SIRT1 activity. These findings open avenue for translational research aimed at enhancing the antiviral capabilities of the elderly. Understanding the deacetylation-mediated control mechanism of IRF3/IRF7 LLPS may explain the diminished antiviral immunity frequently observed in aged patients with viral infections. Targeting the modulation of SIRT1 activity could potentially lead to the development of therapies that boost innate immune responses and address the medical challenges posed by aging^266^ (Fig. 5c). We summarized the molecular characteristics for condensates of cGAS-STING signaling components in Table 2.

**Table 2:** Summary of molecular characteristics for condensates of cGAS-STING pathway

cGAS-STING components	Subcellular localization	Condensates component	Expression of IRF dependent target genes	Function	Pathophysiological effects	Ref.
cGAS	Cytoplasm	dsDNA	Up	Protection dsDNA from exonuclease TREX1	Activation of innate immune response	^242,244^
		Ku70/80		dsDNA co-sensor of cGAS		^249^
		USP15		Dynamic regulator of cGAS-dsDNA condensates		^250^
		G3BP1		Formation of cGAS-G3BP1 primary condensates		^247^
		ZYG11B		Enhancement of G3BP1-cGAS interaction		^248^
		Spermine		Condensation of dsDNA and promoting of cGAS-dsDNA condensates formation		^265^
		RNA, Zn^2+^		Promoting of cGAS-dsDNA condensates formation		^242,247^
		PCBP2	Down	Decrease of cGAS enzymatic activity	Suppression of innate immune response	^251^
		SIRT2		Deacetylation of G3BP1 and negative regulation cGAS-dsDNA condensates		^252^
		Oleic acid		Dissolution of cGAS condensates		^261^
		kicGAS		Binding dsDNA competitively with cGAS	Immune evasion	^255^
		ORF52/VP22 Tegument protein				^254^
		SARS-CoV-2 nucleocapsid protein				^258^
STING	Cytoplasm	STING-cGAMP-TBK1	Down	Formation of puzzle-like structure of STING phase-separator and Holding cGAMP and TBK1 within condensates	Preventing overactivation of immune response	^253^
		Mutation form of NF2		Reduction of STING-initiated antitumor immunity	Suppression of innate immune response	^213^
IRF3/7	Nucleus	SIRT1	Up	Deacetylation of IRF3/7	Relieve of immunosenescence	^266^

*dsDNA* double-stranded DNA, *USP15* ubiquitin specific peptidase 15, *G3BP1* Ras GTPase-activating protein-binding protein 1, *ZYG11B* Zyg-11 family member B, *PCBP2* poly(rC) binding protein 2, *SIRT* Sirtuins, *kicGAS* Kaposi’s sarcoma–associated herpesvirus (KSHV) inhibitor of cGAS

#### Effect of inhibitors on cGAS-STING-related phase separation

XQ2B, discovered through cyclopeptide screening to find inhibitors of cGAS, binds instead to the DNA binding site of cGAS and inhibits DNA-induced cGAS activation and the activity of liquid condensate. In particular, TREX1, which is sequestered through condensate formation, is reactivated by XQ2B treatment and reappears on DNA, promoting DNA degradation.^244^ XQ2B treatment in TREX KO mice efficiently suppressed systemic inflammation, enhanced HSV-1 infection and reduced cGAS-STING-mediated antiviral immunity, while maintaining the cytostatic effect of cGAS, and efficiently inhibited systemic inflammation.^267^ EGCG, a G3BP1 inhibitor, a component of green tea leaves, reduced the primary condensates of cGAS-G3BP1 and ultimately reduced the formation of cGAS-dsDNA condensates and blocked IFN-I production when cells were treated. These drugs selectively degrade cGAS-DNA condensates and could be used as drug targets for autoimmune and hyperimmune responses.^247^ Conversely, treatment with AGK2, a selective inhibitor of SIRT2, maintained the acetylation of G3BP1, which promoted the formation of cGAS-DNA condensates via primary condensates of cGAS-G3BP1 and further increased type I IFN levels and the expression of IFN-stimulated genes. These drugs may act as enhancers of anti-cancer and antiviral immunity by inhibiting the activity of factors that interfere with the formation of cGAS-dsDNA condensates.^252^

### Phase separation of TGF- β signaling pathway

#### Brief overview of TGF-β signaling pathway

Transforming growth factor β (TGF-β) signaling represents a multifunctional pathway crucial for diverse biological processes ranging from embryonic development to tissue homeostasis and disease progression.^268–271^ The TGF-β family shares a structure comprising a secretion signal peptide, a prodomain, and a mature TGF-β domain. These ligands exert their biological effects by binding to specific receptor complexes on the cell surface. The canonical TGF-β pathway primarily involves the interaction between TGF-β ligands and the tetrameric receptor complex consisting of TGF-β receptor II (TβRII) and TGF-β receptor I (TβRI, also known as ALK5). Upon ligand binding, TβRII phosphorylates TβRI, leading to the activation and subsequent phosphorylation of receptor-regulated Smads (R-Smads), specifically Smad2 and Smad3. Activation of R-Smads facilitates their association with the common mediator Smad (Co-Smad), SMAD4, forming complexes that translocate into the nucleus to regulate the transcription of target genes crucial for various cellular processes.^271–275^ In addition to the canonical pathway, TGF-β signaling can also engage in noncanonical pathways that operate independently of SMAD proteins. These pathways include but are not limited to ERK signaling, Rho GTPase signaling, p38 MAPK signaling, JNK signaling, NF-κB signaling, PI3K/AKT signaling, and JAK/STAT signaling.^276^ Activation of these noncanonical pathways allows TGF-β to exert diverse biological effects depending on cellular context and microenvironmental cues.

#### TGF-β pathway phase separation in tumorigenesis

**Fig. 6:**
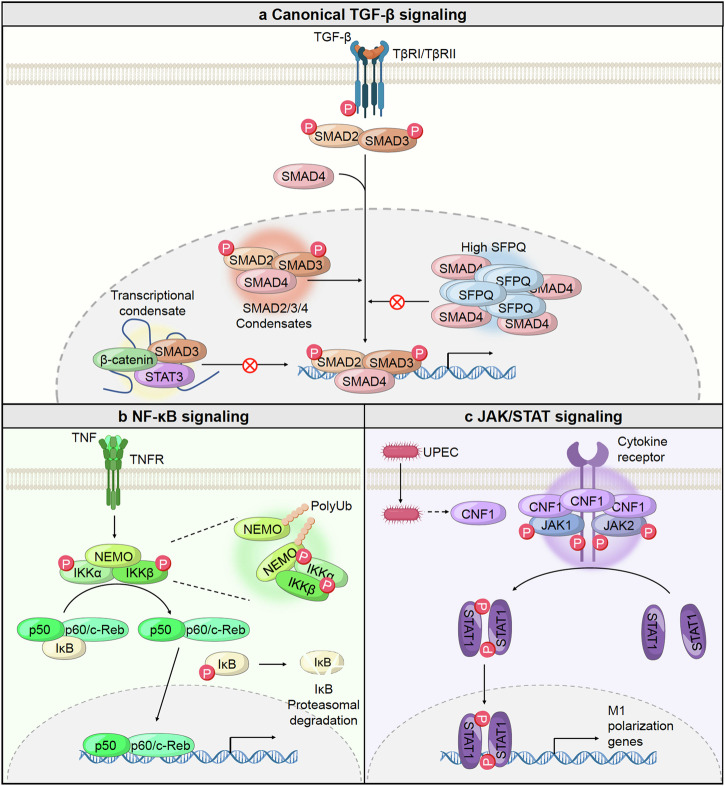
Molecular mechanisms of phase separation in TGF-β, NF-κB, and JAK-STAT pathway. **a** Phase separation in regulation of TGF-β signaling pathway: In canonical TGF-β signaling, in response to the secretion signal peptide, Smad transducer proteins (SMAD2/3) are phosphorylated by type I receptor kinase and translocate to the nucleus with the SMAD4 to regulate transcriptional responses. SMAD2/3-SMAD4 complex facilitates the DNA binding and activates the TGF-β signal. SFPQ LLPS isolated SMAD4 from the SMAD2/3 complex and impeded SMAD-dependent gene expression. SFPQ recruits TDP-43 and FUS to the SMAD4, triggering the formation of paraspeckles and transcriptional regulation. Transcriptional condensates formed by signaling factors β-catenin, STAT3, and SMAD3 selectively engage with super-enhancer-associated genes, thereby driving cell-type-specific responses, while also interfering with TGF-β signaling. **b** NEMO Phase Separation in regulation of NF-κB signaling pathway: Upon cytokine activation, such as TNF, NEMO plays a crucial role in connecting NF-κB signaling pathways. NEMO recruits components of the IκB kinase (IKKα and IKKβ) and polyubiquitin chains, facilitating the phosphorylation of IKKβ by TAK1. This leads to autophosphorylation of IκB and subsequent degradation, allowing NF-κB dimers like p50 and p60/c-Rel to rapidly translocate to the nucleus and activate NF-κB signaling. **c** CNF1 phase separation and M1 macrophage polarization via JAK/STAT signaling: Bacterial toxin CNF1, produced during bacterial infections, forms condensates around cytokine receptors even without external stimuli. CNF1 directly binds to JAK1/2, significantly enhancing their phosphorylation. This activation subsequently induces phosphorylation and dimerization of STAT1. As a result, CNF1 promotes M1 macrophage polarization and contributes to inflammatory dysregulation through the JAK/STAT signaling pathway

The critical role of TGF-β in cancer progression, highlighting its multifaceted influence on cellular processes such as epithelial-mesenchymal transition (EMT) and ECM remodeling, which contribute to tumor invasion, metastasis, and resistance to therapies.^271–275^ A hallmark feature of many cancers is the loss of responsiveness to TGF-β, which enables cancer cells to evade growth inhibition and immune surveillance, thereby facilitating tumor progression. The molecular mechanisms underlying TGF-β resistance in cancer cells remain incompletely understood, indicating a significant challenge in cancer biology and therapy development. The SMAD2/3/4 complex has been shown to undergo LLPS and bind to the promoter region of the TAT (Tyrosine aminotransferase) gene.^277^ This interaction facilitates HCC invasion and promotes EMT by activating pro-EMT inducers. Moreover, recent research has identified SFPQ as a novel regulator of TGF-β signaling in cancer cells. SFPQ, an RNA-binding protein ubiquitously expressed in cells, is involved in alternative splicing, transcriptional regulation, and the formation of paraspeckles in association with ALS-associated RBPs TDP-43 (TAR DNA-binding protein 43) and FUS.^278^ Notably, SFPQ is frequently upregulated in various human cancers and has been suggested as a potent suppressor of TGF-β signaling pathways. The suppressive effect of SFPQ on TGF-β responses is mediated through its PrLD, which drives LLPS. Mechanistically, SFPQ is able to induce phase separation to sequestrate Smad4 physically, disrupting the assembly of active transcriptional complexes crucial for Smad-dependent gene expression induced by TGF-β. Thus, SFPQ significantly attenuates TGF-β‘s tumor-suppressive activities, thereby altering the cellular response to TGF-β and potentially promoting conditions that favor tumorigenesis and cancer progression (Fig. 6a).

In addition to SFPQ, multiple biomolecular condensates appear to modulate Recent studies have expanded our understanding of how TGF-β signaling is modulated by biomolecular condensates. Building on prior research showing that macromolecules undergo phase separation through multivalent interactions, a high-throughput proteome-wide analysis has now identified 75 phase separation proteins associated with TGF-β signaling.^279^ During the short-term activation of TGF-β signaling, these condensates appear to play a role in the negative regulation of canonical Wnt signaling, aligning with earlier.^280^ In contrast, long-term TGF-β exposure leads to the formation of condensates involved in focal adhesion, RNA binding, tight junctions, and Hippo signaling pathways.^281^ These findings suggest the need for further exploration into the potential crosstalk between TGF-β signaling and other pathways via phase separation mechanisms.

### Phase separation of NF-κB signaling pathway

#### Brief overview of NF-κB signaling pathway

Nuclear factor-κB (NF-κB) comprises inducible transcription factors crucial for regulating innate and adaptive immunity, inflammatory responses, cell proliferation, and apoptosis.^282–284^ This system consists of two main pathways: the canonical pathway and the noncanonical pathway, each characterized by distinct components and biological functions.^285,286^ The canonical NF-κB pathway is activated by extracellular signals from Toll-like receptors, TNF receptors, B-cell receptors, and TCR. Upon activation, transforming growth factor-β-activated kinase 1 (TAK1) interacts with the IκB kinase (IKK) complex, leading to the phosphorylation and subsequent degradation of NF-κB inhibitors (IκBs). This degradation allows NF-κB transcription factors to translocate to the nucleus and activate a variety of inflammatory target genes.^287,288^ The IKK complex comprises two catalytic subunits, IKKα and IKKβ, and a regulatory subunit called NEMO or IKKγ.^289,290^ On the other hand, the noncanonical NF-κB pathway is activated by members of the TNF receptor superfamily such as BAFFR, CD40, Lymphotoxin β receptor, and receptor activator of NF-κB ligand (RANK).^291^ In this pathway, upon phosphorylation of IKKα, NF-κB-inducing kinase (NIK) stimulates the degradation of the C-terminal IκB-like structure of p100. Upon p100 degradation, RelB is released and dimerizes with p52, forming a RelB-p52 complex that translocates to the nucleus to activate specific target genes.^292^ NF-κB proteins exist in various forms, including homo-dimers and heterodimers, each with distinct functional roles. For example, the RelA (p65) and cRel heterodimer primarily acts as a transcriptional activator, promoting gene expression, whereas p50 or p52 homodimers typically function as transcriptional repressors. The specific and regulatory combinations of NF-κB dimers result in a wide range of cellular responses, indicating the complexity and versatility of the NF-κB signaling pathway.^283^

#### Phase separation as NEMO-dependent regulatory mechanism for NF-κB activation

LLPS potentially represents a critical regulatory stage in immune responses and related diseases, as supported by increasing research.^293–295^ Central to this process is the NF-κB essential modulator NEMO, a critical component of the inhibitor of κB kinase (IKK) complex,^296,297^ indispensable for canonical NF-κB signaling downstream of diverse immune receptors. Upon activation by cytokines like TNF or IL-1β, NEMO interacts with polyubiquitin (polyUb) chains, particularly those linked via Lys63 (K63) or M1 linkages. This interaction induces NEMO’s oligomerization and triggers the formation of liquid-like droplets through phase separation. This phase separation is driven by multivalent interactions between NEMO and polyUb, facilitated by key domains such as the NEMO ubiquitin-binding (NUB) domain and zinc-finger (ZF) domain. These interactions are crucial for initiating IKK activation within cells, a prerequisite for NF-κB pathway activation.^293–295^ Impairments in NEMO’s phase separation capability due to mutations are associated with various human immunodeficiencies and cancers, primarily due to compromised NF-κB activation. Furthermore, NEMO’s role extends beyond NF-κB signaling into NF-κB-independent functions, particularly in maintaining cellular proteostasis by promoting the clearance of protein aggregates through autophagy. Under conditions of proteotoxic stress, NEMO-deficient cells exhibit impaired protein aggregate clearance, highlighting its broader implications in cellular homeostasis. Recent studies have elucidated additional molecular mechanisms whereby the intervening domain (IVD) of NEMO coordinates signal-induced conformational changes and facilitates the formation of disulfide-bonded dimers upon IL-1β stimulation, dependent on M1-ubiquitin linkages.^298^ Taken together, NEMO integrates signals from innate immune receptors to modulate NF-κB signaling activation via phase separation induced by polyUb binding, thereby driving immune responses and contributing to cellular homeostasis maintenance (Fig. 6b).

#### Effect of phase separation on NF-κB signaling pathway in viral infection

Single-cell sequencing and RNA-sequencing studies conducted on peripheral blood mononuclear cells and bronchoalveolar immune cells from COVID-19 patients have consistently demonstrated that SARS-CoV-2 infection triggers robust activation of the NF-κB pathway.^299–302^ Despite these findings, the precise mechanisms through which SARS-CoV-2 induces NF-κB activation are still uninvestigated. Recent research has highlighted the role of the nucleocapsid protein (N protein) of SARS-CoV-2 in not only facilitating viral replication but also modulating immune responses via the NF-κB signaling pathway.^303–306^ Similar to the cGAS-STING pathway described earlier, SARS-CoV-2 induces hyper-activation of NF-κB and subsequent inflammation. The N protein of SARS-CoV-2 promotes NF-κB signaling activation by enhancing the association between TAK1 and the IKK complex. Through its interaction with viral RNA, the N protein forms membrane-less organelles and recruits TAK1 and the IKK complex, thereby facilitating NF-κB activation. Importantly, LLPS mediated by the N protein is critical for SARS-CoV-2-induced NF-κB activation. Studies have demonstrated that inhibition of LLPS using compounds like 1,6-hexanediol, which widely used reagent to disrupt condensate formation by blocking hydrophobic interactions, attenuates NF-κB activation triggered by SARS-CoV-2. This suggests that targeting LLPS could potentially serve as a therapeutic strategy for mitigating inflammation in COVID-19 patients. Additionally, LLPS has been observed in other RNA viruses such as respiratory syncytial virus (RSV), where it plays a contrasting role by sequestering the p65 subunit of NF-κB in perinuclear puncta. This sequestration inhibits the nuclear translocation of p65, thereby dampening the innate immune responses mediated by the NF-κB signaling pathway.^307^

### Phase separation of JAK/STAT signaling pathway

#### Brief overview of JAK/STAT signaling pathway

The JAK/STAT signaling pathway constitutes a fundamental mechanism in cellular communication, involved in crucial biological processes such as development, immune response modulation, and cell differentiation.^308,309^ This pathway responds to a diverse array of cytokines, growth factors, and hormones, including interferons, interleukins, and colony-stimulating factors, which bind to specific receptors.^310^ Central to the functionality of the JAK/STAT pathway are its key components: ligand-receptor complexes, Janus kinases (JAKs), and signal transducers and activators of transcription (STATs).^311^ The mammalian JAK family comprises four members—JAK1, JAK2, JAK3, and TYK2—each distinguished by a unique set of structural domains that dictate their functional specificity.^312–314^ Upon ligand binding to the receptor, JAKs undergo conformational changes that activate their kinase function. This activation leads to the phosphorylation of specific tyrosine residues on the receptor itself and subsequent recruitment and phosphorylation of STAT proteins.^311^ Phosphorylated STATs form homodimers or heterodimers via their SH2 domains, enabling their nuclear translocation. Inside the nucleus, STAT dimers bind to responsive DNA elements and regulate the transcription of target genes essential for various cellular responses. The structure of STAT proteins includes an N-terminal domain (NTD) for dimerization, a CC domain for protein-protein interactions, a DNA-binding domain, a linker domain for flexibility, an SH2 domain for phosphotyrosine recognition, and a transcription-activation domain.^311^

Dysregulation of the JAK/STAT pathway plays a pivotal role in the pathogenesis of a wide range of diseases, encompassing cancers and autoimmune disorders, where abnormal activation of this signaling cascade drives disease progression. In cancer, for instance, hyperactivation of JAK/STAT signaling can promote uncontrolled cell proliferation, survival, and metastasis, contributing to tumor growth and resistance to treatment. Similarly, in autoimmune diseases, such as rheumatoid arthritis and lupus, overactive JAK/STAT signaling can lead to excessive immune responses and tissue damage.^315–318^ Given its central role in disease pathophysiology, therapeutic strategies aimed at targeting components of the JAK/STAT pathway have emerged as promising approaches for managing these conditions. Small molecule inhibitors that selectively target JAKs or interfere with STAT activation have shown efficacy in preclinical and clinical studies. These inhibitors work by blocking specific steps in the pathway, thereby reducing inflammatory responses in autoimmune diseases or inhibiting tumor cell proliferation and survival in cancers. For example, JAK inhibitors such as Tofacitinib and Ruxolitinib have been approved for the treatment of rheumatoid arthritis, psoriasis, and certain types of blood cancers.^319^ These inhibitors offer targeted therapy options that aim to restore normal immune function or suppress aberrant cell growth driven by dysregulated JAK/STAT signaling. Ongoing research continues to explore new therapeutic agents and strategies to further optimize treatment outcomes and expand the therapeutic spectrum of JAK/STAT pathway modulation in various diseases.

#### Phase separation dynamics of JAK/STAT signaling in macrophage polarization

Macrophages play a crucial role in the innate immune response, serving as pivotal defenders during urinary tract infections (UTIs) against bacterial invasion. While essential for clearing pathogens, macrophages must strike a delicate balance; excessive activity can lead to heightened inflammation and tissue damage. M1 macrophages, activated by T_h_1 cytokines like IFN-γ and TNF-α or bacterial lipopolysaccharide, express pro-inflammatory genes such as TNF-α, IL-6, IL-12, and IL-1β to combat infections. M2 macrophages, induced by T_h_2 cytokines IL-4 and IL-13 via STAT6 activation, promote anti-inflammatory responses and tissue repair.^320,321^ The dynamic polarization of macrophages in response to microenvironmental cues is critical in shaping disease outcomes, influencing both initiation and progression.^322–325^ For instance, bacterial toxin cytotoxic necrotizing factor 1 (CNF1) from uropathogenic *Escherichia coli* (UPEC) induces urinary tract inflammation and impairs macrophage phagocytosis.^326–328^ Recent studies have revealed that CNF1 promotes M1 macrophage polarization through a mechanism independent of Rho GTPase but dependent on NF-κB and JAK-STAT signaling pathways.^329–331^ Specifically, CNF1 interacts physically with JAK1 and JAK2, leading to enhanced STAT1 phosphorylation and the formation of phase-separated CNF1-JAK1-JAK2 complexes. This condensate drives M1 polarization and triggers an inflammatory response in the kidneys during acute UTIs.^330,332^ The phase separation dynamics of JAK/STAT pathways in macrophages represent a crucial aspect of immune regulation and disease pathology (Fig. 6c). We summarized the molecular characteristics for condensates of TGF- β, NF-κB, JAK/STAT signaling components in Table 3.

**Table 3:** Summary of molecular characteristics for condensates of TGF-β, NF-κB, and JAK/STAT pathway components

Signal transduction	Signaling transduction components	Domain necessary for phase separation	Condensate components	Function	Pathophysiological effect	Ref.
TGF-β	SMAD3	-	β-catenin, STAT3, Nanog	Mediates the cell-type specificity of the response to signaling at super-enhancer driven genes	-	^542^
	SMAD4	MH2	SFPQ	Inhibition of TGF-β tumor-suppressive responses	HCC tumorigenesis	^646^
	SMAD2/3/4	TAT gene promotor site	-	Regulate the expression of TAT	Inhibit HCC progression	^647^
NF-κB	NEMO	NUB (Ub binding site)	NEMO, IKKα/β, Ubiquitin, TAK1, TAB1, TAB2/3	Essential for IKK activation	-	^293^
JAK/STAT	JAK1/2	-	CNF1, JAK1/2	Promote the M2 macrophage polarization	Urinary tract infections	^330^

*MH2* Mad Homology 2, *TAT* tyrosine aminotransferase, *NUB* N-ubiquitin, *TAK1* transforming growth factor-β activated kinase 1, *TAB* TAK1-binding protein, *HCC* hepatocellular carcinoma

### Phase separation of MAPK/ERK signaling pathway

#### Brief overview of MAPK/ERK pathway

The MAPK/ERK signaling pathway regulates important intracellular processes such as cell proliferation, growth, and differentiation in response to a variety of external signals, including growth factors, hormones, and cytokines.^333–338^ The pathway begins when specific receptors on the cell membrane sense an external stimulus and, following ligand binding, induce the activation of intrinsic tyrosine kinases. This activation leads to the recruitment of RAS proteins, and adaptor proteins such as Grb2 and SOS1 play an important role in this process 16760435.^339^ RAS is activated by exchanging GDP for GTP, which in turn activates RAF proteins (A-Raf, B-Raf, and C-Raf),^340,341^ triggering a phosphorylation chain reaction that leads to MAP3K, MAP2K, and ERK.^342–344^ Finally, activated ERK translocates to the nucleus where it phosphorylates nuclear targets, such as transcription factors, to directly regulate gene expression. The precise regulation of the MAPK/ERK pathway is essential for maintaining tissue homeostasis and organ function, and aberrant activation of this pathway is observed in several diseases, including cancer.^345–349^ In particular, aberrant MAPK/ERK activation plays an important role in tumor formation, progression, and metastasis, and recent studies suggest that a novel mechanism called phase separation is involved in the dynamic regulation of this signaling pathway. The MAPK/ERK pathway also plays an important role in T cell activation, and the development of targeted therapeutics to restore proper function of the pathway has emerged as an important challenge.^350,351^

#### Phase separation in T cell activation: MAPK/ERK pathway

**Fig. 7:**
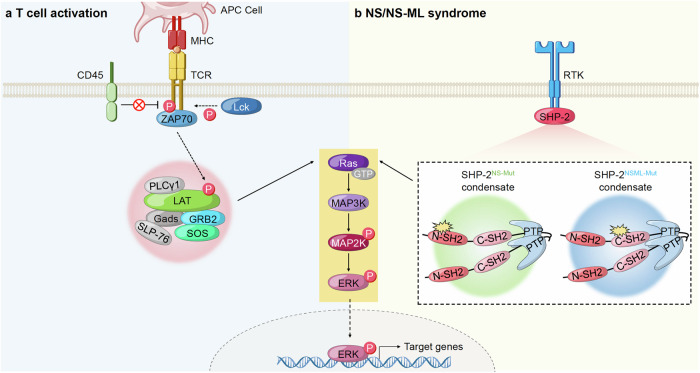
Phase separation-mediated MAPK/ERK activation of T cell signaling pathways. **a** Upon activation of the T cell receptor (TCR) by an MHC-antigen complex, CD45 enzymatic activity is disrupted, allowing Lck to phosphorylate the TCR. This event promotes the membrane enrichment of ZAP70 protein, which phosphorylates LAT (Linker for Activation of T cells). LAT then undergoes phase separation and recruits its binding partner proteins, forming signaling complexes that act as upstream signal transducers of the MAPK/ERK pathway. This activation cascade ultimately enhances T cell signaling and immune responses. **b** Mutations in the SHP2 protein associated with Noonan syndrome and related disorders (NS/NS-ML syndrome) lead to phase separation-mediated recruitment of wild-type SHP2 protein. This recruitment enhances activation of the MAPK/ERK pathway, contributing to dysregulated cell signaling observed in these syndromes

The MAPK/ERK pathway is instrumental in preserving immune homeostasis, keeping a delicate balance between robust pathogen responses and preventing autoimmunity or chronic inflammation.^352,353^ In particular, T cells, originating in the thymus and essential for detecting and eliminating invading antigens, rely significantly on the MAPK/ERK pathway for their optimal functionality.^354–357^ T cell receptors (TCRs) are central to this process, as they recognize antigens presented by antigen-presenting cells (APCs) and orchestrate the immune response. An intriguing aspect arises when TCRs engage with antigens, prompting their coalescence with associated signaling molecules into phase-separated microdomains on the T cell membrane. This clustering enhances signal transmission by centralizing signaling components and promoting their interactions.^358–360^ Recent studies have shed light on the underlying mechanism behind the formation of liquid-like, micrometer-sized clusters. Phosphorylation of the TCR by Lck recruits and activates ZAP70, which in turn phosphorylates LAT.^361,362^ This leads to multivalent assembly and subsequent phase separation of LAT. Upon TCR activation, LAT, along with other adapter proteins, forms phase-separated condensates, thereby facilitating downstream signaling pathways such as MAPK/ERK activation.^363^ Furthermore, it has been reported that PLCγ1 also promotes the formation of T cell activation microreactor condensates. PLCγ1 interacts with a scaffold protein called LAT to promote cluster formation, and these clusters act as condensates to concentrate signaling molecules into high concentrations. During this process, LAT remains phosphorylated and is physically inaccessible to dephosphorylating enzymes such as CD45, allowing signal transduction to proceed efficiently. The SH2 and SH3 domains of PLCγ1 bind to LAT clusters and recruit proteins such as Grb2, which further activate the signaling pathway. In addition, the concentration of PLCγ1 regulates cluster size and stability, which is important for T cells to regulate signal strength and persistence in response to different physiological conditions. Interestingly, PLCγ1 can be overexpressed in pathological conditions such as cancer, which can lead to the formation of abnormally large LAT clusters, which trigger excessive signaling, which can contribute to abnormal cell growth and survival^364^ (Fig. 7a).

#### MAPK/ERK phase separation in disease-associated mutations

The Src homology region 2 domain-containing phosphatase-2 (SHP2) encoded by *PTPN11*, is a non-receptor protein tyrosine phosphatases (PTPs) required for MAPK signaling pathway.^365–367^ Germline heterozygous mutations in *PTPN11* gene are closely associated to various diseases, Noonan syndrome (NS), and Noonan syndrome with multiple lentigines (NS-ML). Interestingly, NS mutations are viewed as gain-of-function (GOF), while NS-ML mutations are considered loss-of-function (LOF). Despite differing activities, both mutations lead to similar clinical manifestations and an increased risk of malignancy, suggesting a complex role for SHP2 in disease pathogenesis.^368–372^ SHP2 is an allosteric enzyme comprising tandem SH2 domains, a central PTP catalytic domain, and a C-terminal tail. Its phosphatase activity is carefully regulated by an autoinhibitory mechanism involving intramolecular.^373,374^ GOF mutations disrupt this autoinhibition, while LOF mutations impair phosphatase activity. Interestingly, both types of mutations promote an open conformation, yet the link to disease mechanisms remains unclear. Both NS/NS-ML and cancer-associated SHP2 mutants gain the ability to undergo LLPS to enhance PTP enzyme activity. The NS-associated SHP2 mutant has a mutation in the SH2 region of the N-terminus of SHP-2, and the NS-ML-associated SHP2 mutant has a mutation in the SH2 region of the C-terminus, both of which mutate to transform SHP2 from a closed to an open conformation, forming a phase separation driven by a conserved PTP domain that is well folded through intermolecular electrostatic interactions. Disease-associated SHP2 mutations also recruited and activated wild-type SHP2 into phase-separated condensates, and somatic mutations of SHP2 in cancer cells also promoted SHP2 LLPS. Together, these ultimately demonstrate that LLPS of cancer and disease-associated SHP2 mutants hyperactivate MAPK signaling through ERK1/2 activation^375^ (Fig. 7b). We summarized the molecular characteristics for condensates of MAPK/ERK pathway in Table 4.

**Table 4:** Summary of molecular characteristics for condensates of MAPK/ERK pathway

Condensate	Subcellular localization	Condensates component	Expression of ERK dependent target genes	Function	Pathophysiological roles	Ref.
TCR condensates	Cytoplasm	ZAP70, LAT, Grb2, Gad, SOS, SLP-76,	Up	Recruiting factors required for T-cell signaling activation	Activation of T cell signaling	^361,362^
		PLC-γ1, LAT, Grb2, SLP-76		Sequestrating CD45 and promoting T cell activation condensates		^362^
Mutant form of SHP-2 condensates		SHP-2 mutants, SHP-2 WT		SHP-2 mutants recruit wild type SHP-2 and forms the LLPS	NS/NS-ML syndrome and cancer associated with SHP-2 mutations	^373^

*TCR* T-cell receptor, *ZAP70* Zeta chain of T cell receptor associated protein kinase 70, *LAT* linker for activation of T cell, *SOS* Son-of-sevenless, *Grb2* growth factor receptor-bound protein 2, *Gad* glutamic acid decarboxylase, *SLP-76* SH2 domain-containing leukocyte phosphoprotein of 76 kDa, *PLC-γ1* phospholipase C, gamma 1, *CNS* Noonan syndrome, *NS-ML* Noonan syndrome with multiple lentigines

### Phase separation of Wnt pathway

#### Brief overview of Wnt/β-catenin pathway

The Wnt signaling pathway, a fundamental signaling cascade, orchestrates critical cellular processes vital for development, tissue homeostasis, and disease pathogenesis.^376–382^ It comprises a complex network of interactions and is delineated into three distinct pathways, each instigated by the binding of Wnt ligands to specific cell surface receptors.^383–389^ Upon activation, Wnt signaling orchestrates a myriad of biological responses mediated by a diverse repertoire of intracellular proteins. The canonical Wnt/β-catenin signaling pathway, primarily governed by the transcription factor β-catenin, exerts profound influences on tissue development, stem cell maintenance, and cell fate determination. At the core of its functionality lies the β-catenin destruction complex (DC), composed of adenomatous polyposis coli (APC),^390,391^ Axis inhibition (AXIN)^390,392,393^ and the kinases glycogen synthase kinase 3β (GSK-3β),^394–396^ and casein kinase 1α (CK1α).^397^ In the absence of Wnt ligands, this complex facilitates the degradation of cytoplasmic β-catenin through phosphorylation and subsequent β-TrCP-mediated ubiquitination, thereby impeding its nuclear translocation.^398,399^ Conversely, upon Wnt binding to its receptors Frizzled (Fzd) and Lipoprotein receptor-related protein 5/6 (LRP5/6), the destruction complex is inactivated, leading to the stabilization and accumulation of β-catenin. The accumulated β-catenin translocates into the nucleus, where it forms complexes with T-cell factor/lymphoid enhancer factor (TCF/LEF) transcription factors,^400,401^ thereby modulating gene expression and eliciting diverse cellular responses. Given that the majority of studies on phase-separated condensates are currently associated with the canonical pathway, this review paper will exclusively focus on the Wnt/β-catenin pathway.

#### Inhibition of Wnt/β-catenin signaling by phase separation-mediated formation of destruction complex

**Fig. 8:**
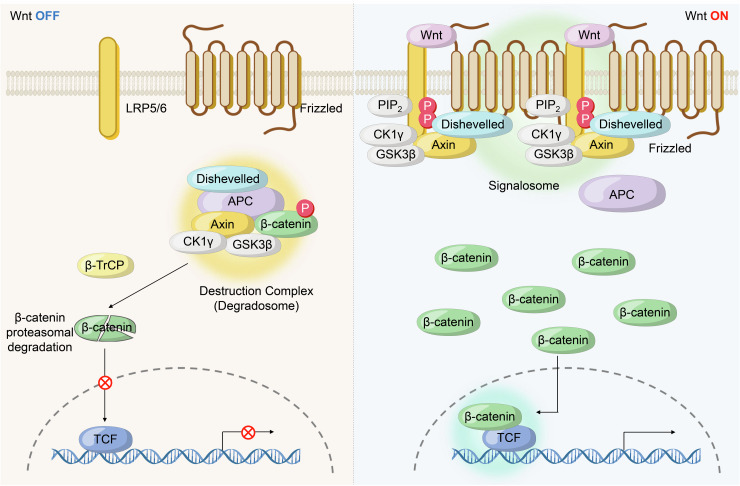
Regulation of Wnt/β-Catenin signaling via biomolecular condensates. In the absence of Wnt ligands, biomolecular condensate formation at the membrane is inhibited, preventing the phase separation of Disheveled, Axin, and GSK-3β into signalosomes. This results in the assembly of the β-catenin destruction complex (Degradosome) in the cytoplasm, consisting of Axin, GSK-3β, APC, and CK1. The Degradosome facilitates the phosphorylation and subsequent ubiquitination of β-catenin, targeting it for proteasomal degradation. As a result, β-catenin levels decrease, thereby preventing its nuclear accumulation and association with TCF/LEF transcription factors. This inhibition suppresses the expression of Wnt-responsive genes involved in cellular functions (Left panel). In the presence of Wnt ligands, Wnt binds to its receptors Frizzled and co-receptor LRP5/6, initiating the formation of biomolecular condensates involving Disheveled, Axin, and GSK-3β at the membrane. These condensates, known as signalosomes, facilitate the sequestration of components that would normally form the β-catenin destruction complex, such as Axin, GSK-3β, APC, and CK1. Consequently, β-catenin accumulates in the cytoplasm due to reduced degradation. Upon accumulation, β-catenin translocates into the nucleus where it binds to TCF/LEF transcription factors, activating the expression of target genes critical for cellular processes like proliferation and differentiation (Right panel)

Recent studies have provided insights into the role of phase separation in modulating the Wnt signaling pathway, specifically focusing on its impact on the assembly and functionality of the β-catenin destruction complex. At the heart of this regulatory mechanism lies the scaffolding protein Axin, which plays a pivotal role in orchestrating the formation and organization of the destruction complex within cellular compartments. Axin’s multifunctional feature may be endowed by its ability to form distinct compartmentalized structures in living cells, contributing to the spatial organization and efficient functioning of the destruction complex.^402–404^ Considering its structural aspects, Axin presents a complex architecture characterized by distinct domain structures that play critical roles in its functional interactions with the main Wnt pathway components. At its N-terminus, Axin harbors a Regulator of G-protein signaling (RGS) domain, which serves as a binding site for the APC, facilitating their interaction. Additionally, Axin possesses a middle region capable of interacting with GSK-3β and β-catenin,^114,405^ pivotal components of the β-catenin destruction complex. Furthermore, Axin features an IDR, adding a layer of flexibility and adaptability to its structural configuration. Moreover, Axin’s structural complexity extends to its C-terminal domain known as the Disheveled-Axin (DAX) domain, which contains another IDR and enables Axin’s self-polymerization and hetero-polymerization with Disheveled through head-to-tail polymerization.^406,407^ The IDR located within Axin’s middle region emerges as a central player in its ability to undergo LLPS, leading to the formation of phase-separated punctate structures. These structures serve as scaffolds for the recruitment of critical signaling components such as GSK-3β, CK1α, and β-catenin, facilitating their interactions and subsequent regulatory processes. Interestingly, while the IDR is indispensable for Axin’s LLPS and functional activities, studies have demonstrated that Axin lacking the DAX domain retains the ability to inhibit punctate formation in vivo and exert influence over the size and number of droplets in vitro.^402,408^ These findings highlight the multifaceted nature of Axin’s structural organization and the dynamic regulation between phase separation and Wnt signaling pathway regulation (Fig. 8).

Axin is a well-characterized, unstable protein that plays a crucial role in Wnt signaling. Increasing the stability of Axin is an effective strategy to modulate this pathway. One mechanism that destabilizes Axin is ADP-ribosylation, a PTM-mediated by the enzyme Tankyrase. This modification leads to Axin’s recognition and subsequent RNF146-mediated proteolysis.^409^ To counteract this, small-molecule inhibitors of Tankyrase, such as XAV-939, have been developed. These inhibitors prevent the ADP-ribosylation of Axin, thereby stabilizing the protein and offering a potential therapeutic approach for cancer.^410,411^ Axin condensates promote phosphorylation by enriching the presence of CK1α over PP1 (Protein Phosphatase 1), thereby facilitating a higher rate of phosphorylation within the condensates. Crucially, this phosphorylation occurs specifically within the Tankyrase-binding site on Axin. The phosphorylation at this site generates both electrostatic and steric hindrance, effectively blocking the interaction between Axin and Tankyrase. By preventing this interaction, the phosphorylation within the Tankyrase-binding site on Axin counteracts the Tankyrase-mediated degradation pathway. This stabilization of Axin allows it to continue its vital role in the Wnt signaling pathway, which is essential for the proper regulation of β-catenin levels and the expression of Wnt target genes.^402,404,412,413^

In addition, recent studies have identified Ubiquitin-specific peptidase 10 (USP10) as an inhibitor of Wnt/β-catenin signaling through a dual-regulation mechanism. Firstly, USP10 exhibits deubiquitinase (DUB) activity, which stabilizes Axin by interacting with its polybasic region and the DIX domain, leading to K48-linked deubiquitination of Axin. This enhances the formation of the β-catenin degradation complex. Secondly, USP10 regulates β-catenin independently of its DUB activity by acting as a scaffold through its IDR. This IDR-mediated interaction induces the formation of Axin granules, stabilizing these structures through phase separation rather than Axin’s typical DAX domain-mediated polymerization. The stabilized, droplet-like Axin structures dynamically undergo fusion and splitting, ultimately leading to the inhibition of β-catenin.^414^

APC is proposed to play a crucial role in the function of Axin and in the formation of the signalosome. Mutational inactivation of APC is a major driver in most colorectal cancers. In this context, LLPS leads to the formation of a β-catenin destruction complex organized by Axin, which degrades β-catenin and downregulates Wnt signaling.^408,415^ Wild-type APC stabilizes Axin-mediated condensates, facilitating their formation and function. In contrast, truncated APC does not affect condensate formation but interferes with the recruitment of GSK-3β and CK1α, resulting in the inhibition of β-catenin degradation.^413^ APC promotes LLPS of Axin by facilitating homo-polymerization through the DAX domain of Axin,^416^ enhancing the dynamics of Axin condensates, and lowering the threshold concentration of Axin required for droplet formation.^404^ Recent studies have shown that APC not only affects the formation of Axin puncta but also forms puncta via APC. The IDR of APC spans the Axin-interacting domain and the β-catenin-interacting 15Rs and 20Rs repeat domains, contributing to LLPS formation.^417^ APC forms LLPS with Axin through the self-association domain and can also form LLPS independently through the 20R3/20R5 domain within the 20R region. The 20R3/20R5 domain is phosphorylated by GSK-3β and CK1α, which are components of the destruction complex and promote β-catenin degradation.^418^ Studies indicate that Axin-mediated LLPS forms a β-catenin destruction complex, a process promoted by APC. Consequently, β-catenin activity is inhibited, leading to the inactivation of Wnt signaling (Fig. 8).

#### Phase separation to activate Wnt signaling pathway

The activation of the Wnt signaling pathway is mediated by key receptors, including Fzd and LRP5/6, which are essential for processes such as body axis patterning, embryonic development, and tissue homeostasis.^419–422^ Upon binding to Wnt ligands, the extracellular domain of LRP5/6 and the cysteine-rich domain (CRD) of Fzd interact, forming a Wnt-Fzd-LRP5/6 ternary complex at the plasma membrane.^423–426^ This complex is essential as it initiates the phosphorylation of five PPPSP motifs in the LRP5/6 tail by GSK-3 and CK1,^388,427,428^ leading to the assembly of LRP6 signalosomes. These signalosomes, which include Fzd, Axin, Dvl, and GSK-3,^429^ are formed in a Dvl-dependent manner, further stabilized by PIP2, and play a crucial role in amplifying Wnt/β-catenin signal transduction.^430,431^ The presence of membrane components such as LRP6 and Fzd is indispensable for the proper function of the signalosome; excluding these components entirely reverses its function. Furthermore, recent studies have shown that the stabilization of LRP6 signalosomes enhances Wnt/β-catenin signaling, which is crucial for promoting intestinal organoid growth and the self-renewal of intestinal stem cells, particularly under osmotic and mechanical stress.^432^

In the complex landscape of Wnt signaling, Disheveled protein emerges as a central player orchestrating critical cellular decisions through its multifaceted interactions and unique structural properties. Among the three isoforms found in mammals, Dvl2 stands out as a key regulator of the Wnt pathway, crucial for steering developmental processes such as cell proliferation, survival, and differentiation.^433^ Central to Dvl2’s function is its capacity to form dynamic protein assemblies. Upon activation by Wnt ligands binding to Fzd, Dvl2 engages in a complex series of molecular interactions. Initially, its N-terminal DIX domain facilitates hetero-oligomerization with Axin via head-to-tail polymerization, thereby disrupting the β-catenin destruction complex.^415,434^ A significant aspect of Dvl2’s signaling mechanism involves phase separation, driven by its IDRs. This process enables Dvl2 to organize into cytoplasmic condensates known as signalosomes, which serve as hubs for Wnt signal transduction.^435,436^ Within these signalosomes, Dvl2 collaborates with receptors such as Fzd5, Fzd8, and Lrp6, enhancing the efficiency and specificity of Wnt signaling pathways.^433,437^ Furthermore, Dvl2’s middle PDZ domain facilitates interactions with various components, including membrane receptors, thereby integrating external signals into the pathway with precision and specificity.^438,439^ Meanwhile, the C-terminal DEP domain stabilizes these interactions, reinforcing the binding of Fzd receptor-PDZ domains and contributing to signalosome integrity.^440,441^ PTMs further regulate Dvl2 activity. Notably, the HECT-E3 ligase WWP2 mediates K63 ubiquitination, a modification crucial for the phase separation of Dvl2 and the subsequent formation of functional signalosomes. This dynamic regulatory mechanism ensures precise spatial and temporal control over Wnt signaling events.^442^ Dvl2 exemplifies a coordinated scaffold protein that bridges the cell surface-localized Wnt receptor signalosome with the cytosolic β-catenin destruction complex.

When Wnt signaling is activated, β-catenin translocates to the nucleus where it interacts with the N-terminal region of TCF/LEF transcription factors. This crucial interaction transforms β-catenin into a co-activator, initiating the expression of target genes essential for various cellular processes. LEF1, a member of the TCF/LEF transcription factor family, assumes a pivotal role in this pathway.^443,444^ A recent study has revealed that upon activation of the Wnt signaling pathway in colorectal cancer cells, LEF1 undergoes a remarkable LLPS with β-catenin. This phenomenon involves the IDR of LEF1, essential for facilitating LLPS. LLPS mediated by the IDR domain of LEF1 is not merely a structural phenomenon but a functional necessity. It serves as a platform that concentrates and organizes β-catenin and LEF1 molecules, thereby facilitating targeted gene transactivation crucial for Wnt signaling. The interaction between LEF1 and β-catenin is pivotal for the formation and dynamics of these biomolecular condensates, ensuring robust and coordinated gene expression in response to Wnt signals. Importantly, LEF1’s promotion of proliferation and migration in colorectal cancer cells is contingent upon its IDR-dependent LLPS. This finding suggests a mechanistic link between Wnt-mediated signaling, phase separation of transcriptional regulators like LEF1, and the pathological processes associated with tumor progression^404,445^ (Fig. 8). We summarized the molecular characteristics for condensates of Wnt/β-catenin signaling components in Table 5.

**Table 5:** Summary of molecular characteristics for condensates of Wnt/β-catenin pathway

Wnt pathway components	Domain necessary for phase separation	Subcellular localization	Condensate components	Expression of Wnt/β-catenin target genes	Function	Pathophysiological effect	Ref.
Wnt, Fzd, LRP6, GSK3, CK1, Axin, Dvl	DIX domain of Dvl and Axin, IDR of Dvl2	Plasma membrane	Wnt, Fzd, LRP6, GSK3, CK1, Axin, Dvl, Clathrin, AP2, PIP_2_,	Up	Inhibit formation of β-catenin destruction complex	Intestinal organoid growth	^413,414,421,424,426,648–650^
Axin, APC, Dvl, β-catenin	DIX domain of Dvl and Axin, N-terminal IDR of Dvl2, IDR of Axin, 20 R domain and ASAD of APC1	Cytoplasm	Axin, APC, Dvl, GSK3, CK1, β-catenin, RNF146, β-TrCP, Tankyrase	Down	β-catenin degradation		^397,406,407,411,412^
β-catenin, TCF	IDR of LEF and β-catenin	Nucleus	β-catenin, TCF	Up	Induce transcription		^436,651^

*DIX* Disheveled and Axin, *ASAD* APC self-association domain, *Fzd* Frizzled, *LRP* low-density lipoprotein receptor-related protein, *Dvl* Disheveled, *APC* Adenomatous polyposis coli, *TCF* T cell factor, *LEF* lymphoid enhancer factor family, *AP2* adipocyte Protein 2, *RNF146* Ring Finger Protein 146, *β-TrCP* Ring Finger Protein 146, *PIP*_*2*_ PtdIns(4,5)P_2_, *GSK3* Glycogen synthase kinase-3, *CK1* Casein kinase I

### Phase separation of Notch pathway

#### Brief overview of Notch pathway

The Notch signaling pathway is a key intercellular communication mechanism that regulates cell fate determination, differentiation, proliferation, and apoptosis, and is activated in mammals by four receptors called Notch1-4 and ligands from the Delta-like and Jagged families.^446–448^ When a ligand binds to a Notch receptor, the ADAM protease cleaves the extracellular domain of the Notch receptor, activating the receptor.^449^ After this cleavage, a γ-secretase complex performs further cleavage within the cell membrane, releasing the Notch Intracellular Domain (NICD).^450^ The released NICD translocates to the nucleus where it binds to Recombination Signal Binding Protein for Immunoglobulin Kappa J Region (RBPJ) and Mastermind-like Coactivators (MAML) to form a transcriptional activation complex. This complex promotes the transcription of genes that are downstream targets of Notch signaling. When NICD binds to RBPJ, the co-repressor (e.g., CSL) dissociates, and the co-activator, MAML, binds and activates gene transcription.^451,452^ The precise regulation of Notch signaling is essential for tissue development and cellular function, and dysregulation of this pathway contributes to a variety of diseases, particularly cancer and developmental disorders.^453–456^ Abnormal activation of Notch signaling can lead to cancer, and conversely, inhibition can lead to developmental disorders. Recent studies have shown that phase separation plays a critical role in the spatial organization and regulation of Notch signaling. This mechanism regulates the formation and positioning of the NICD-RBPJ-MAML complex, enabling dynamic regulation of signaling. These findings advance our functional understanding of the Notch signaling pathway and provide important insights for the development of novel therapeutic strategies.^457^

#### Notch transcriptional condensates in gene regulation

**Fig. 9:**
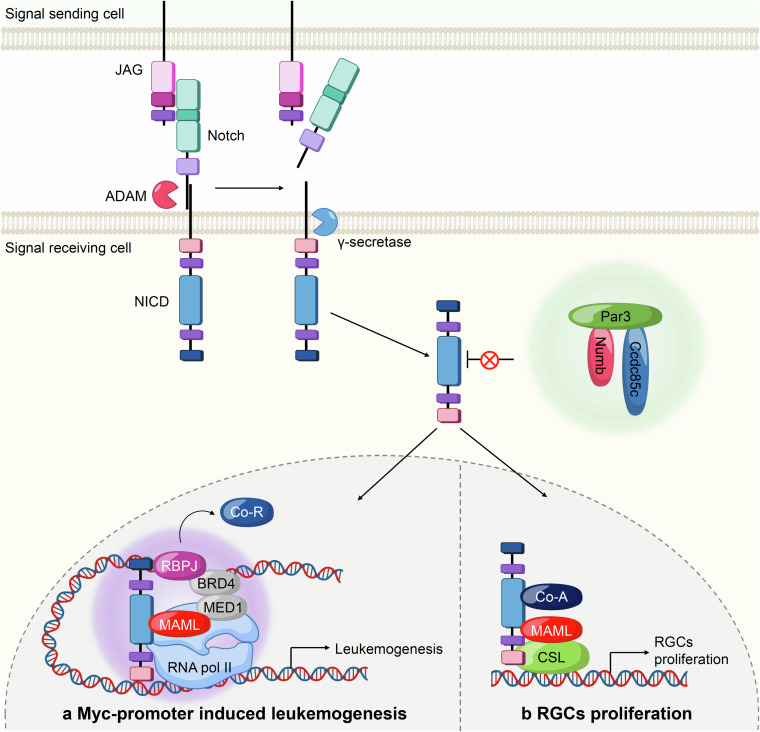
Phase separation in Notch signaling for oncogenic Myc activation and developmental fate control. **a** NICD-Dependent Super-Enhancer Loop in T-ALL Cells. In human T-cell acute lymphoblastic leukemia (T-ALL) cells, Notch Intracellular Domain (NICD)-dependent super-enhancer loops activate the proto-oncogene Myc. This activation is facilitated by a phase-separated transcription machinery that sequesters Co-R and recruits RNA polymerase II (RNA pol II), BRD4, MED1, MAML, and RBPJ. These components form condensates that enhance the transcriptional activity required for Myc expression, contributing to oncogenic transformation in T-ALL. **b** Numb regulation of NICD during asymmetric cell division. Numb, a negative regulator of NICD signaling during asymmetric cell division, undergoes phase separation with polarity proteins Par3 and Ccdc85c. This phase separation sequesters Numb away from the Notch signaling pathway, promoting radial glial cell differentiation and proliferation. By sequestering Numb, phase-separated condensates modulate Notch pathway dynamics, influencing cellular fate decisions during development

Accumulating evidences reveal that enhancer-looping, a critical gene regulation mechanism, is controlled by transcription and regulatory factors undergoing LLPS.^458–461^ This process gives rise to specialized nuclear phase condensates known as transcriptional condensates, creating the transcriptionally active environment to enhance gene expression. Several pivotal transcriptional regulators, including YAP/TAZ, MED1, and histone acetyltransferase P300, have been identified as operators of transcriptional condensates.^19,127^ The phase separation of these transcriptional regulators into condensates suggests the importance of LLPS in the spatial and temporal regulation of gene expression. By concentrating and organizing transcriptional machinery and regulatory proteins, transcriptional condensates provide a mechanism for cells to achieve high levels of gene expression with precision and efficiency. However, despite the well-established roles and mechanisms in other signaling pathways, the formation and operational mechanisms of transcriptional condensates remain largely unexplored in the context of Notch signaling. Recently, Foran et al. demonstrated that NICD forms functional transcriptional condensates to efficiently activate gene expression by enriching transcriptional machinery at target genomic loci. In line with findings from other signaling pathways, they observed NICD-dependent SE looping and subsequent activation of the proto-oncogene Myc in human T-cell acute lymphoblastic leukemia (T-ALL) cells, facilitated by transcriptional condensates^462^ (Fig. 9a).

#### Notch signaling regulation through phase separation in cell polarity

Cell polarity is a fundamental attribute crucial for coordinating diverse cellular processes and influencing both cellular signaling and tissue architecture. In polarized cells, specific protein complexes, such as Par-3/Par-6/aPKC, dynamically regulate cellular asymmetry by condensing at distinct membrane domains in response to internal and external cues.^463–467^ Notch signaling plays a pivotal role in orchestrating asymmetric cell division and maintaining cell polarity, thereby exerting profound influences on organogenesis. This signaling pathway is carefully and precisely regulated to ensure precise cellular outcomes, with Numb acting as a key negative regulator.^468,469^ Previous works have demonstrated that during asymmetric cell division in *drosophila* neuroblasts, distinct protein complexes exhibit asymmetric localization. In particular, the Notch pathway inhibitor Numb is found at the basal cortex of neuroblasts, a process controlled by the Par-3/Par-6/aPKC complex, Partner of Numb (Pon), and Polo kinase. aPKC phosphorylation prevents Numb and Pon from attaching to the apical cortex, whereas Polo kinase phosphorylation targets Pon and Numb to the basal complex.^470–473^ Research has clarified that the PTB domain of the protein Numb recognizes particular motifs on Pon, enabling a multivalent interaction that triggers LLPS of the Numb/Pon complex in *drosophila*. This condensed phase is notably dense and dynamically interacts with cytoplasmic proteins. Crucially, the formation of these condensates through the multivalent interaction between Pon and Numb is essential for promoting the differentiation of larval neuroblasts and for maintaining Numb-mediated inhibition of Notch signaling.^474^ Radial Glial Cells (RGCs) are progenitor cells for the generation of most cortical neurons, playing an important role in neuronal proliferation and differentiation. These cells exhibit highly polarity, regulated by various polarity proteins. Par3 engages with Ccdc85c to form a condensate. This condensate alters the closed conformation of Par3 and binds to Numb, thereby enhancing neuronal differentiation and proliferation through modulation of Notch signaling. Thus, this LLPS-mediated mechanism represents an additional regulatory layer for the polarity control complex.^128,475^ This finely tuned regulation ensures the fidelity of cellular processes necessary for proper tissue development and homeostasis. Cell polarity orchestrates the precise spatial organization of proteins and organelles within cells, enabling them to carry out specialized functions and respond effectively to diverse stimuli. Considering its relevance to human disease, Dysregulation of phase separation dynamics involving polarity proteins and their complexes could potentially impair cellular asymmetry and disrupt normal tissue architecture. These disruptions have been implicated in a spectrum of human diseases, including developmental abnormalities, tissue degeneration, and the metastatic progression of cancer. In such contexts, the loss of cell polarity often correlates with increased cellular motility and invasive behavior, contributing to disease progression. Therefore, unraveling the regulatory role of phase separation in governing the spatial organization and function of polarity complexes in both human physiology and disease is paramount (Fig. 9b). We summarized the molecular characteristics for condensates of Notch signaling in Table 6.

**Table 6:** Summary of molecular characteristics for condensates of NOTCH pathway

Condensate	Subcellular localization	Condensates component	Expression of NICD target genes	Function	Pathophysiological roles	Ref.
NICD	Nucleus	NICD, RNA polymerase II, MAML, MED1, BRD4, RBPJ	Up	Recruiting transcriptional machinery to myc-promoter via DNA looping	Myc-promoter induced T-ALL leukemogenesis	^451^
Numb		Par3, Numb, Ccdc85c		Sequestering Numb, negative regulators of Notch signaling	RGC proliferation	^462^

*NICD* Notch intracellular domain, *MAML* mastermind-like, *RBPJ* Recombination Signal Binding Protein For Immunoglobulin Kappa J Region, *T-ALL* T-cell acute lymphoblastic leukemia, *RGC* Retinal ganglion cells, *Ccdc85c* Coiled-coil domain containing 85c, *Par3* partitioning defective protein 3

### Phase separation of mTOR pathway

#### Brief overview of mTOR pathway

The mammalian Target of Rapamycin complex 1 (mTORC1) is a crucial and evolutionarily conserved effector that orchestrates cell growth and metabolism in response to various stimuli, including growth factors, cellular energy levels, and amino acids.^476,477^ Its dysregulation is implicated in numerous diseases such as cancer, metabolic disorders, aging, and diabetes.^477–486^ mTOR functions as part of two distinct protein complexes: mTORC1 and mTORC2. mTORC1 is characterized by its specific binding partners, the regulatory-associated protein of mTOR (RAPTOR)^480^, and the proline-rich Akt substrate of 40 kDa (PRAS40).^487,488^ Conversely, mTORC2 includes the rapamycin-insensitive companion of mTOR (RICTOR) along with other binding partners. The roles of these complexes differ significantly. mTORC1 serves as a cellular nutrient sensor; when cells are in a nutrient-rich environment, mTORC1 is activated to promote anabolic processes and cell growth. mTORC2, on the other hand, is primarily associated with protein phosphorylation, impacting cell survival, growth, cytoskeletal organization, migration, division, and signaling pathways.^489–495^

The activation mechanism of mTORC1, particularly through growth factors (such as insulin) and amino acids, is well elucidated. Upon stimulation by growth factors, the phosphoinositide 3-kinase (PI3K) pathway is activated. PI3K phosphorylates phosphatidylinositol 4,5-bisphosphate (PIP2) to produce phosphatidylinositol 3,4,5-trisphosphate (PIP3).^496^ This phosphorylation event is mediated via the insulin receptor (IR) and its substrate. Subsequently, phosphoinositide-dependent kinase-1 (PDK1) phosphorylates and activates protein kinase B (AKT). Activated AKT, in turn, inhibits the Tuberous Sclerosis Complex (TSC), a negative regulator of mTORC1.^497,498^ The inhibition of TSC leads to the activation of mTORC1 substrates, such as ribosomal protein S6 kinase beta-1 (S6K1), eukaryotic initiation factor 4E-binding protein 1 (4EBP1), and PRAS40.^499^ These events collectively drive cell growth and proliferation. Moreover, mTORC1 is responsive to cellular energy levels. When energy is scarce, AMP-activated protein kinase (AMPK) is activated. AMPK, in turn, activates the TSC complex and inhibits mTORC1, thereby conserving energy and suppressing anabolic activities under low energy conditions. This energy-sensing pathway highlights mTORC1’s role in maintaining cellular energy homeostasis.^500^ Overall, mTORC1 dysregulation is central to the pathology of various diseases, making it a critical target for therapeutic interventions aimed at restoring cellular homeostasis. Recent research has unveiled an intriguing aspect of mTOR signaling: its involvement in phase separation. The concept of phase separation adds a new layer of complexity to our understanding of mTOR signaling, highlighting its importance in the dynamic regulation of cellular functions.

#### Reciprocal crosstalk between mTOR and stress granule

Stress granules (SGs) are dynamic, membrane-less organelles that form within the cytoplasm of eukaryotic cells in response to various stress conditions, including oxidative stress, heat shock, nutrient deprivation, and viral infections.^501–504^ Unlike membrane-bound organelles, stress granules lack a surrounding lipid bilayer, allowing for rapid assembly and disassembly as dictated by cellular conditions. These transient structures play crucial roles in cellular stress responses, serving as protective repositories that sequester mRNAs and RBPs, thereby halting translation and conserving cellular energy.^502,505–507^ This adaptive mechanism enables cells to survive adverse conditions and recover once the stress subsides.

**Fig. 10:**
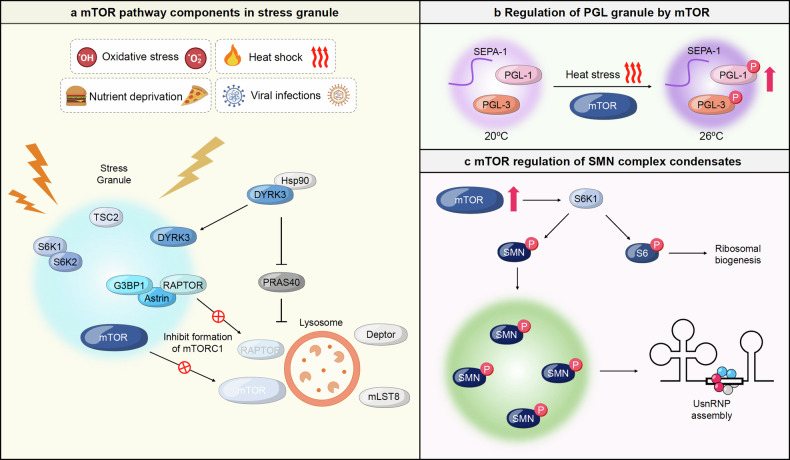
Regulation of mTORC1 pathway by condensate. **a** Stress granules (SGs) formed in response to cellular stress incorporate components of the mTORC1 pathway, including TSC2, mTOR, S6K1/2, and RAPTOR. These SGs act as dynamic assemblies that sequester various proteins, altering cellular signaling under stress conditions. During cellular stress, DYRK3, a stress-responsive kinase, is inactivated and sequestered within SGs. This process sequesters mTOR away from the cytoplasm, preventing its assembly into mTORC1 complexes and thereby inhibiting downstream signaling pathways involved in RNA translation and protein synthesis. **b** mTORC1-mediated phosphorylation of PGL-1/-3 triggers stress-induced phase separation, forming PGL granules that resist autophagic degradation. **c** Spliceosomes are essential for processing RNA transcripts in eukaryotic cells by removing introns and joining exons in pre-mRNAs. They are composed of UsnRNPs, which are synthesized in a complex, tightly regulated process. mTOR acts as potential regulators of SMN complex condensates, linking them to RNA processing and cellular signaling

Extensive research has unveiled a complex relationship between stress granules and the inhibition of mTOR pathway,^508,509^ which has provided profound insights into the mechanisms underlying this relationship. Central to this molecular mechanism is the phosphorylation of eukaryotic initiation factor 2 alpha (eIF2α), a pivotal regulator of translation initiation, alongside the involvement of other translation factors like the mRNA helicase eIF4A.^510–513^ These factors collaborate to orchestrate the formation and regulation of SGs, dynamic cytoplasmic structures devoid of membranes but enriched with mRNA, translation initiation complexes, and RBPs. When cells encounter stress, particularly oxidative stress, SGs emerge as a crucial intrinsic mechanism for cell survival.^514,515^ Remarkably, key components of the mTOR pathway, including S6K1 and S6K2,^516^ recognized substrates of mTORC1, and TSC2,^517^ a critical subunit of the TSC complex, have been found to localize within SGs. The dynamics of SGs are complicatedly regulated, with S6K1 and S6K2 playing distinct roles. S6K1 initiates SG formation by phosphorylating eIF2α, while S6K2 functions to uphold the integrity of assembled SGs.^518^ This dual regulation underscores the significance of S6 kinases in governing SG dynamics and emphasizes the link between mTOR signaling and cellular stress responses. Furthermore, under stress conditions, TSC2 undergoes a notable translocation from its usual association with the lysosome to SGs. This relocation is facilitated by its interaction with HDLBP, a critical protein involved in SG organization. The recruitment of TSC2 to SGs adds another layer of complexity to mTOR signaling regulation, accentuating the complex interplay between translation inhibition and cellular survival mechanisms.^509,517,519^ Astrin, known for its role as a negative regulator of mTORC1, undergoes upregulation in response to oxidative stress. Mechanistically, Astrin orchestrates the recruitment of RAPTOR, a critical component of mTORC1, to stress granules. Within these SGs, RAPTOR’s association with Astrin inhibits the binding of mTORC1 to RAPTOR, effectively impeding mTORC1-mediated hyperactivation-induced apoptosis and thwarting the formation of anti-apoptotic stress granules.^508^ Moreover, the cellular stress response involves the participation of DYRK3, a dual-specificity kinase implicated in various physiological processes including cell cycle regulation, apoptosis, and DNA damage response.^520^ In response to cellular stress, DYRK3 undergoes inactivation and translocates to SGs via its N-terminal IDR. Once localized within SGs, DYRK3 phosphorylates PRAS40, a known inhibitor of mTORC1. This phosphorylation event effectively dampens mTORC1 activity, stabilizing stress granules and sequestering mTOR away from the cytosol. This spatial sequestration of mTOR within SGs enhances its inhibitory effect, further contributing to the cellular stress response. Overall, stress granules serve as dynamic entities that are tightly regulated by various cellular stresses, including those encountered in cancer-related contexts. The reciprocal connection between Astrin, RAPTOR, DYRK3, and mTOR within stress granules unveils novel regulatory mechanisms that govern cellular responses to stress, offering valuable insights into the multifaceted nature of cellular stress adaptation and survival mechanisms (Fig. 10a).

Obesity represents a significant global health concern, predisposing individuals to various medical complications, including an increased risk of cancer development and elevated mortality rates.^521–524^ Obesity induces cellular stress, encompassing endoplasmic reticulum (ER), oxidative, genotoxic, and biomechanical stress, which contribute to the pathogenesis of obesity-associated cancers.^525–527^ Recent investigations have shed light on SGs as potential mediators of cancer development, particularly in the context of obesity-associated pancreatic ductal adenocarcinoma (PDAC). Specifically, studies have revealed an augmented formation of SGs in response to cellular stress in individuals with obesity-related PDAC, a casual linked to the overactivation of IGF1/PI3K/mTOR/S6K1 pathway by serine/arginine protein kinase 2 (SRPK2).^528^

In summary, stress granules SGs, dynamic and transient biomolecular condensate structures induced by cellular stress, play a crucial role in cellular stress responses by interacting with the mTOR pathway to regulate cell survival and adaptation. The regulation of SG dynamics involves key components like S6 kinases, TSC2, Astrin, and DYRK3, illustrating the complexity of cellular stress adaptation mechanisms. Understanding these mechanisms provides valuable insights into broader cellular stress responses and survival strategies. Moreover, recent findings linking obesity to cancer emphasize the need to explore novel pathways, such as SG formation, in cancer development. SGs may serve as mediators of cancer progression in obese individuals, offering a perspective on the mechanisms underlying obesity-associated cancers. Overall, investigating SGs in the context of cellular stress and cancer development offers promising avenues for future research and therapeutic interventions to mitigate the adverse effects of obesity on cancer risk and progression.

#### Regulation of PGL granules by mTOR

LLPS, often triggered by multivalent interactions among proteins or protein-interacting domains, is crucial for the formation of various cellular structures, including RNP granules like stress granules and P bodies.^529,530^ These granules, formed through LLPS-mediated protein-RNA interactions, contribute significantly to cellular organization and function. Additionally, LLPS-mediated formation of RNP granules, such as stress granules and P bodies, involves protein-RNA interactions, further highlighting the versatility of phase separation in cellular organization.^6,17^ One extensively studied example of LLPS is the assembly of the germline P granule in one-cell-stage *C. elegans* embryos.^13^ The establishment of distinct boundaries of P granules along the anterior-posterior axis is comprehensively regulated by gradients of scaffold proteins, whose LLPS properties are modulated by RNA-binding and spatially controlled phosphorylation. As embryonic development progresses, P granules become specifically localized in germline blastomeres and are eventually partitioned into germline precursor cells.^13,531^ During asymmetric cell divisions that generate germline blastomeres, selective removal of components from P granules occurs, with proteins such as PGL-1 and PGL-3 being targeted for degradation by autophagy.^532,533^ Recent research has unveiled a novel aspect of LLPS regulation under heat-stress conditions. It has been discovered that mTORC1-mediated phosphorylation of PGL-1/-3 acts as a switch-like stress sensor, accelerating phase separation and leading to the formation of PGL granules resistant to autophagic degradation. This accumulation of PGL granules serves a critical role in maintaining embryonic viability during heat stress, underscoring the pivotal involvement of mTORC1 signaling in coordinating LLPS dynamics to facilitate cellular adaptation during development^534^ (Fig. 10b).

#### Role of mTOR in cytoplasmic phase separation and biophysics

Cellular function depends on complex interactions of molecular processes in which molecular crowding events play a central role.^535^ Within the crowded cellular environment, high concentrations of molecules dictate the efficiency of various biological processes. Notably, molecular crowding has been shown to profoundly influence phase separation, a process wherein proteins form liquid droplets within cells.^536^ The study of macromolecular crowding often involves observing the movement of tracer particles within cells, offering insights into cellular biophysics. Genetically encoded multimeric (GEM) nanoparticles have emerged as invaluable tools in this realm, providing a non-invasive means to analyze molecular dynamics. Leveraging GEMs, recent research has uncovered the regulatory role of the mTORC1 kinase in ribosome abundance, shedding light on its impact on cellular biophysical properties and phase separation dynamics. Inhibition of mTORC1 has been found to significantly alter ribosome concentration, thereby influencing particle diffusion and the behavior of phase-separated entities in both yeast and human cells. This comprehensive study uncovers the indispensable role of macromolecular crowding in cellular physiology and highlights its profound influence on phase separation phenomena.^537^

#### mTOR regulation of SMN complex condensates for RNA processing

Spliceosomes, complex molecular machines found in the cells of eukaryotes, are essential for precisely processing RNA transcripts. They achieve this by removing non-coding introns and joining coding exons in pre-messenger RNAs (mRNAs).^538,539^ These spliceosomes are composed of uridine-rich small nuclear ribonucleoproteins (UsnRNPs), crucial components that ensure the accuracy of RNA processing. The production of UsnRNPs is a highly regulated process, reflecting their pivotal role in cellular function. They are synthesized abundantly, especially in actively dividing cells, where the demand for RNA processing is high and must be carefully coordinated with cellular growth and nutrient availability. The synthesis of UsnRNPs involves a series of complex steps occurring in both the nucleus and cytoplasm.^540,541^ Initially, UsnRNAs, the RNA components of UsnRNPs, are transcribed and processed within the nucleus before being exported to the cytoplasm. There, they undergo assembly with Sm proteins to form functional core particles. This assembly process is facilitated by the chaperone activity of the survival motor neuron (SMN) complex.^542–544^ Once assembled, these UsnRNPs undergo further maturation within the nucleus, often within specialized subnuclear biomolecular structures called Cajal bodies. The condensation of the SMN complex within Cajal bodies is an intriguing phenomenon that may optimize the maturation process of UsnRNPs.^545–548^ However, the precise mechanisms underlying this phenomenon are still under investigation. Recent research has focused on unraveling the regulatory mechanisms governing the function of SMN complex condensates. In particular, various phosphatases and kinases, including mTOR/RPS6KB1, have emerged as potential regulators of SMN complex activity, indicating associations between biomolecular condensates, molecular machinery and cellular signaling pathways in RNA processing^549^ (Fig. 10c). We summarized the molecular characteristics for condensates of mTOR signaling components in Table 7.

**Table 7:** Summary of molecular characteristics for condensates of mTOR pathway

mTOR pathway components	Domain necessary for phase separation	Subcellular localization	Condensate components	Expression of mTOR dependent target genes	Function	Pathophysiological effect	Ref.
mTOR, S6K1/2, TSC2, RAPTOR	LCR of DYRK3	Cytoplasm	mTOR, TSC2, S6K1/2, G3BP1, Astrin, RAPTOR, DYRK3	Down	Inhibit formation of mTOR complex 1		^494,501,504,505^
mTOR-mediated phosphorylation induces Phase separation		Cytoplasm, PGL granule	PGL1/3, SEPA-1, EPG-2,	-	Regulates PGL granule formation	Regulation of autophagic degradation during development	^520^
		Cytoplasm SMN	SMN complex (SMN, Gemins, sm Protein),	-	-	UsnRNP biogenesis	^535^

*S6K* Ribosomal protein S6 kinase beta-1, *TSC* Tuberous sclerosis complex, *RAPTOR* Regulatory-associated protein of mTOR, *LCR* low-complexity regions, *DYRK3* Dual Specificity Tyrosine Phosphorylation Regulated Kinase 3, *PGL1/3* Phenolic glycolipid–1/3, *EPG-2* Ectopic P granules protein 2, *SMN* survival motor neuron, *UsnRNP* U-rich small nuclear ribonucleoprotein particles

### Phase separation-mediated crosstalk between signaling pathways

The determination and maintenance of cell identity, with its unique properties during development or in adult tissue, are regulated by the combined actions of cell type-specific master transcription factors and signaling molecules that guide transcriptional programs. During processes like cell trans-differentiation and reprogramming, these combined actions are essential for controlling cell type-specific gene expression. For instance, the Yamanaka factors -Oct4, Sox2, Klf4, and Myc- are well-documented for their capacity to reprogram induced pluripotent stem cells (iPSCs) from various cell types, including fibroblasts.^550–552^ Similarly, MyoD is a key factor for trans-differentiation into muscle-like cells.^553^ During mammalian development, cells depend on the exquisite and sequential actions of complex activators and effector molecules from pathways such as WNT, TGF-β, and JAK/STAT to sustain their identity and respond to external stimuli.^309,376,554,555^ Recent studies have shown that these master transcription factors, along with mediator coactivators and DNA-binding factors, form IDR-mediated condensates. These condensates act as SEs, playing a crucial role in regulating gene expression with high specificity and efficiency.^556^

Furthermore, it has become increasingly apparent that various signaling pathways can interact to form phase separations, regulating numerous cellular processes such as skeletal muscle development and bone metastasis.^557–562^ The phase separation-dependent crosstalk between Wnt and TGF-β/SMAD signaling is particularly significant in bone metastasis, where Wnt and TGF-β antagonize each other through specific molecular condensates, balancing bone formation and resorption.^280^ During myogenesis, the Hippo signaling effector TAZ has been shown to undergo phase separation, inhibiting skeletal myogenesis through interactions with SMAD7 and β-catenin.^563^ The Smad7-β-catenin complex plays a crucial role in mammalian myogenesis, with protein-protein interactions at muscle-specific promoters being essential for the repression of Smad7 and β-catenin function.^564^ In myogenic cells, TAZ exhibits phase separation properties, resulting in its accumulation in the nucleus, where it activates transcription complexes related to cell proliferation and growth while inhibiting differentiation by acting as a co-repressor of the myogenic transcription complex, which includes Smad-β-catenin. During myoblast differentiation, Hippo signaling is activated, leading to the phosphorylation of TAZ. This phosphorylation causes TAZ to relocate to the cytoplasm, trapping β-catenin in the nuclear envelope, thereby inhibiting the activity of Smad7 and β-catenin at myogenesis-related promoters such as Myod1 and Myog. This process induces myogenic differentiation, establishing TAZ as a critical determinant of myogenic differentiation.^563^ The phase separation-mediated crosstalk between these signaling pathways and their transcription factors has emerged as a fundamental mechanism in regulating cell identity, development, and disease pathology.

## Therapeutic strategies targeting biomolecular condensates

Traditional drug targets, such as specific enzymes or receptors, often present difficulties due to their undruggable nature, including issues like lack of suitable binding sites or high specificity requirements.^565–567^ One promising approach to overcoming the challenges of drug development is through targeting phase separation in cells. By focusing on phase separation, researchers can target the dynamic and collective interactions that drive the formation of biomolecular condensates, which are critical for various cellular processes and are increasingly recognized as being closely linked to the pathology of several diseases. Additionally, phase separation targeting drugs can address complex diseases by disrupting pathological condensates or restoring normal condensate function, providing a new avenue for developing drugs against previously undruggable targets. By influencing the assembly or disassembly of these condensates, small molecules can effectively alter the function of proteins that are otherwise difficult to target directly with conventional therapies. This approach allows for the regulation of complex protein networks and cellular processes that are critical in diseases such as cancer and neurodegenerative disorders. Moreover, targeting biomolecular condensates can provide a more refined control over cellular environments, potentially reducing off-target effects and improving therapeutic specificity.

### Small molecules and chemical probes modulating condensate formation

As mentioned earlier, there are various regulatory mechanisms of biomolecular condensates and small molecules can interact with phase-separating biomolecular condensates through various forces such as π-π interactions, cation-anion interactions, dipole-dipole interactions, hydrophobic interactions, and electrostatic interactions. These interactions can modulate the formation, stability, and dynamics of the condensates. Here are some key examples of how small molecules affect these interactions.

ATP plays a crucial role in modulating phase separation through its ability to influence electrostatic interactions within biomolecular condensates. ATP is a negatively charged molecule that can disrupt electrostatic interactions between positively charged regions of proteins, which are often essential for phase separation. By binding to these positively charged domains, ATP can prevent the formation of condensates or promote their dissolution.^568–570^ Conversely, ATP can also act as a hydrotrope, maintaining proteins in a soluble state and preventing unwanted aggregation.^571^ The balance of ATP concentration within the cell is, therefore, critical in regulating phase separation, as it can either stabilize or destabilize condensates depending on the cellular context and the specific proteins involved. This regulatory mechanism highlights the importance of ATP in cellular organization and its potential as a target for modulating phase separation-related processes in disease contexts.

Curcumin, a small natural molecule, interacts with α-Synuclein (α-Syn) during phase separation by binding to the hydrophobic regions of α-Syn, which are important for its aggregation. By doing so, curcumin decreases the fluidity of α-Syn inside the condensates, effectively delaying or inhibiting the transition to amyloid fibrils. Furthermore, curcumin can destabilize preformed α-Syn amyloid aggregates in the condensates.^572^

Epigallocatechin gallate (EGCG) also directly binds to hydrophobic protein sequences both by hydrophobic interactions and by hydrogen bonding.^573^ EGCG can modulate the phase separation behavior of α-Syn and Amyloid-β (Aβ) fibrils, maintaining it in a less aggregated, more soluble state. This action reduces the risk of forming the pathological aggregates associated with neurodegeneration.^574,575^

These probes are indispensable for gaining insights into condensate biophysics, including formation, stability, and responses to environmental cues. They facilitate the visualization of condensates in live cells, providing dynamic snapshots of their spatial organization and behavior, crucial for understanding their roles in cellular processes like signaling and transcriptional regulation.

Aβ peptides are associated with Alzheimer’s disease and are known to undergo LLPS as an early step in forming amyloid fibrils. These fibrils are formed through interactions that include π-π stacking among aromatic residues such as phenylalanine, tyrosine, and histidine within the Aβ sequence. Fluorescent dye Thioflavin-T which is gold standards for selectively staining and identifying amyloid fibrils binds to Aβ through π-π stacking not only serves as a fluorescent marker for amyloid fibrils but also can stabilize the aggregates, thereby influencing the assembly and physical properties of these condensates.^576,577^

In addition, another well-known condensate probe is 1,6-hexanediol. This compound is commonly used to disrupt LLPS condensates that are formed primarily through weak physical interactions, such as hydrogen bonding and π-π interactions.^29,70^ 1,6-hexanediol was originally noticed for its ability to disrupt FG repeat interactions between nucleoporins in the nuclear pore complex and interactions between RBPs in RNP granules^578,579^ but 1,6-hexanediol is widely used to study protein/RNA condensates/bodies.^580,581^

Traditional methods for studying and observing LLPS, such as FRAP, FRET (Fluorescence Resonance Energy Transfer), and fluorescence microscopy, have provided valuable insights but are often limited by temporal and material constraints. These techniques require significant time and resources to capture the dynamic processes of phase separation and often struggle with real-time observation and quantification. The development of Pyr-A, a dipyrene-based fluorescent probe, represents a significant advancement in the study of LLPS. Pyr-A is uniquely designed to detect and monitor the formation and dynamics of phase-separated biomolecules by exploiting its sensitivity to changes in environmental polarity and viscosity.^582^ This fluorescence-switching capability allows Pyr-A to provide real-time, ratiometric measurements of the physicochemical dynamics within condensates, offering a more efficient and direct method for researching phase separation dynamics. However, while Pyr-A excels as a diagnostic tool for studying LLPS, its role as a direct therapeutic agent is limited. In the broader context of condensate-modulating small molecules and chemical probes, Pyr-A can serve as an essential component of the toolkit for understanding the complex behaviors of biomolecular condensates. While Pyr-A currently requires counterstaining with specific markers to spatially define the targets within cells, future modifications could significantly enhance its functionality. By incorporating functionalized nuclear localization signal peptides or antisense sequences specific for target RNAs, it may become possible to precisely control the intracellular destination of Pyr-A.^582^

### Targeting condensate components for therapeutic intervention

Previously, we discussed how various signaling molecules in different signaling pathways are regulated through LLPS. These diverse signaling pathways are crucial for controlling human biological processes and can lead to various diseases when they function improperly. Therefore, these signaling molecules have been considered targets for disease therapeutics; however, proteins with undefined structures, transcription factors, super enhancers, RNAs, and PTMs were regarded as undruggable targets.^64^ However, emerging evidence suggests that these condensates possess druggable potential.^8,583^ We have discussed various mechanisms by which small molecules can exert their effects. In this part, we will explore how drugs or drug candidates can specifically act on different targets and diseases, examining their practical applications in therapeutic interventions.^584^

MED1 is a crucial component of the mediator complex, a multiprotein assembly essential for transcriptional regulation. It plays a particularly significant role in the regulation of genes located within SE regions, which are vital for driving high levels of transcription of key genes related to cell identity and various disease states, including cancer.^585^ The aromatic amino acids present in MED1 enhance the physical properties of biomolecular condensates, facilitating the concentration of other molecules.

Research has demonstrated that the IDR of MED1 can form phase-separated droplets in vitro when enriched with transcriptional units in nuclear extracts and it has been shown to form nuclear condensates in SE regions.^458^ In several cancers, such as prostate and breast cancer, robust transcription of genes driven by specific genomic regions designated as SEs results in mutations and overexpression that contribute to tumor growth, metastasis, and treatment resistance.^586,587^ Structural alterations in MED1 lead to quantitative changes that enhance the expression of specific genes when bound to estrogen receptors (ER) within the condensate.^588^

The development of MED1 inhibitors remains in its early stages, with no products yet reaching commercialization or active clinical trials. Efforts to create MED1-targeted therapeutics face significant challenges. MED1 is known to be integral not only to transcriptional regulation but also to cellular growth and survival.^458^ Therefore, the direct inhibition of MED1 could potentially have detrimental effects on normal cells. Additionally, the intricate signaling pathways that involve multiple proteins and genes raise concerns regarding the risk of eliciting incorrect signals, which may have unforeseen consequences on cellular function.^589^ Interestingly, cisplatin exhibits a preferential accumulation within the MED1 condensate. This localization indicates that cisplatin may enhance its interaction with DNA within these condensates, leading to significant disruptions in the transcriptional regulatory network.^590^ Consequently, the accumulation of cisplatin not only promotes more effective binding to DNA but also compromises the integrity of the transcriptional regulatory framework associated with the MED1 condensate. This phenomenon elucidates why cisplatin is effective across a range of cancers.^591^

JQ1, a potent inhibitor of Bromodomain-Containing Protein 4 (BRD4), has also been shown to preferentially localize within MED1 condensates, alongside other critical molecules involved in transcription regulation.^590^ The increased concentration of JQ1 within these condensates can disrupt transcriptional activities by selectively affecting the expression of oncogenes associated with SE regions where MED1 condensates reside.^592,593^ Thus, JQ1 demonstrates the potential for targeting cancer cells that depend on MED1 condensates.

The combined treatment with both drugs enhances the structural instability of the condensate, with JQ1 contributing to this instability and cisplatin facilitating DNA platination.^594^ This synergistic effect can selectively modulate the MED1 condensate. Although these two drugs operate through distinct mechanisms, they share the common characteristic of targeting MED1 condensates in cancer, offering a therapeutic intervention.^595^

EPI-001 and THZ1 represent innovative approaches in MED1-dependent therapeutic strategies for androgen-resistant cancer. EPI-001 targets the NTD of the androgen receptor (AR) and inhibits the interaction with MED1. The AR is a primary oncogenic driver in prostate cancer (PCa) and is characterized by extensive disordered regions.^596^ In androgen-dependent PCa cells, AR is co-recruited with MED1 condensates to SE regions, thereby promoting oncogenic transcriptional programs.^597^ Treatment with EPI-001 in PCa cells has been shown to reduce the formation of AR droplets, indicating its potential utility in therapies aimed at disrupting the AR-MED1 interaction in AR-driven cancers.^598^

THZ1, on the other hand, inhibits CDK7-mediated phosphorylation of MED1 at threonine 1457, a modification critical for MED1’s functional role in enhancing AR-driven transcription in PCa cells.^599^ By suppressing MED1 hyperphosphorylation, THZ1 disrupts the interaction between MED1 and AR, resulting in a significant decrease in the expression of AR target genes.^600^ This disruption leads to diminished formation of MED1 condensates, reduced transcriptional activation, and impaired proliferation of cancer cells.^601^

Collectively, these findings suggest that targeting the CDK7-MED1 axis with THZ1 offers a promising therapeutic strategy for treating PCa that has developed resistance to AR-targeted therapies.

Mutations in the p53 gene, such as R175H and Y220C, result in misfolding of the protein, leading to a loss of its tumor-suppressive function. These misfolded p53 proteins exhibit a propensity to form aggregates through phase separation.^602^ Such aggregates not only disrupt the normal function of p53 but also sequester other members of the p53 family, including p63 and p73, thereby further diminishing tumor-suppressive activities and promoting oncogenic processes.^603^ In cancers where these mutations are prevalent, such as breast and lung cancers, the accumulation of p53 aggregates has been associated with more aggressive disease progression and resistance to conventional therapies.^604,605^

Recent studies have investigated the compounds BAY 249716 and BAY 1892005 for their capacity to modulate the aggregation of mutant p53. These compounds have shown promise in dissolving aggregates formed by structurally destabilized p53 mutations like R175H and Y220C, potentially restoring some normal p53 functions and slowing cancer progression.^606^ However, in the case of mutations such as R248Q, which primarily impact p53’s DNA-binding ability rather than its structural integrity, BAY compounds may inadvertently promote further aggregation.^607^ This emphasizes the critical need for careful application, highlighting the significance of adapting treatment strategies to the specific p53 mutation identified in the tumor.^603,608^

The principal challenge resides in developing therapies that selectively target mutant forms of p53 without compromising the function of the wild-type protein or interfering with other crucial cellular processes.^609^ Further research is essential to optimize the specificity of BAY compounds and to elucidate their comprehensive effects across various p53 mutations.^610^ Future directions may involve the advancement of personalized medicine strategies that customize the use of these compounds according to the genetic profile of an individual’s cancer, thereby maximizing therapeutic efficacy while minimizing potential side effects.

Nuclear receptor-binding SET domain protein 2 (NSD-2) is a histone methyltransferase that regulates gene expression through the dimethylation of lysine 36 on histone 3 (H3K36me2).^611^ Steroid receptor coactivator-3 (SRC-3) is a transcriptional activator protein that has an important role in gene regulation and possesses an IDR.^612^ In multiple myeloma, NSD2 facilitates LLPS of SRC-3, thereby enhancing malignant oncological processes in cancer cells and contributing to treatment resistance.^613^ SI-2 is an inhibitor that impedes the function of SRC-3, disrupting the interaction between NSD2 and SRC-3, which in turn suppresses SRC-3-mediated LLPS.^614^ This leads to enhanced efficacy of anticancer drugs and presents a potential strategy for overcoming drug resistance. Consequently, SI-2 represents a promising therapeutic approach for the treatment of multiple myeloma.

Oxaliplatin, an anticancer agent, disrupts nucleolar function, which is critical for ribosome biogenesis. Nucleoli are formed through LLPS, a process driven by the intrinsic properties of nucleolar proteins. These IDRs facilitate phase separation by promoting multivalent interactions among proteins and RNA, resulting in the formation of distinct nucleolar sub-phases: the fibrillar center, dense fibrillar component, and granular component (GC). Each of these sub-phases has specific roles related to ribosome assembly.^615^ Oxaliplatin interferes with the LLPS process within nucleoli, particularly affecting key proteins such as fibrillarin (FBL) and nucleophosmin (NPM1), which are essential for maintaining nucleolar integrity. The disruption caused by oxaliplatin impairs the multivalent interactions necessary for LLPS, leading to the disintegration of the nucleolar structure. Consequently, the nucleolar assembly line responsible for ribosomal RNA (rRNA) synthesis is compromised, resulting in the inhibition of rRNA production.^616^ This disruption triggers cell cycle arrest and ultimately induces cell death. Oxaliplatin selectively targets nucleoli, making it especially effective against cancer cells that heavily depend on translation, such as colorectal cancer.^617^ This drug introduces a novel mechanism for cancer treatment by disrupting the organization of nucleolar proteins.

FUS is an RNA-binding protein that plays a critical role in gene transcription and RNA processing, forming condensates essential for cellular stress responses and organizational functions.^618^ However, under pathological conditions, these condensates can transition into solid aggregates, which are implicated in neurodegenerative diseases such as amyotrophic lateral sclerosis (ALS) and frontotemporal dementia (FTD).^619,620^

Mitoxantrone is an anti-cancer agent that interacts with DNA and disrupts the cell cycle, particularly by interfering with the condensate formation of RBPs such as TDP-43.^621,622^ TDP-43 is a crucial protein in nerve cells that binds to RNA and regulates transcription and splicing. It is known to form abnormal condensates in various neurological disorders, most notably ALS.^623,624^

Under stress conditions, TDP-43 forms condensates, a process vital for the cellular stress response. However, excessive condensate formation can destabilize RNA transcription and splicing, ultimately impairing neuronal function. In vitro studies have demonstrated that mitoxantrone reduces the co-localization of TDP-43 with G3BP1, which is essential for stress granule formation.^625^ This reduction in interaction helps maintain normal neuronal functions, such as neurite outgrowth. In essence, when mitoxantrone inhibits the abnormal condensation of TDP-43, it normalizes RNA transcription and splicing, thereby preserving neuronal function.^626^ TDP-43 has been implicated in neurodegenerative conditions, including ALS and FTD.^627^ Recently, various drugs and antibodies have been developed to target TDP-43, aiming to maintain its healthy function by preventing its aggregation and abnormal accumulation.^628^ Thus, mitoxantrone presents the potential to mitigate the progression of neurodegenerative diseases like ALS by affecting the formation of TDP-43 aggregates.

To further elucidate this beneficial effect on neurons, it is critical to conduct an in-depth analysis of the interactions between TDP-43 and other RBPs, as well as to clarify the overall mechanism of action of mitoxantrone.^629,630^

Acute myeloid leukemia (AML) is profoundly affected by mutations in the NPM1, particularly in its mutant form known as NPMc+. These mutations are present in a significant proportion of AML patients and are characterized by the mislocalization of the NPMc+ protein, which is typically found in the nucleolus, to the cytoplasm.^631,632^ This aberrant localization is critical for the pathogenesis of leukemogenesis. The mutations that result in changes in the C-terminal domain (CTD) of NPM1 disrupt its normal protein-protein interactions, thereby increasing the propensity for protein aggregation. Such aggregation is likely the consequence of dysregulated protein folding or inappropriate cellular localization, ultimately disrupting cellular function and contributing to the pathophysiology of the disease.^633^ In therapeutic development, avrainvillamide (AVA) has potential that promotes nuclear retention of NPMc+. By facilitating the nuclear condensation of NPMc+, AVA reduces the expression levels of CRM1 and FLT3, both of which are involved in the proliferation of leukemia cells.^634^ Consequently, by ensuring that NPMc+ remains localized in the nucleus, AVA represents a novel approach to alleviate associated NPM1 mutations.

Heat shock factor 1 (HSF1) functions as a pivotal transcription factor in regulating the cellular stress response by inducing the expression of heat shock proteins (HSPs), which are essential for proper protein folding and the maintenance of cellular homeostasis.^635,636^ Under non-stressful conditions, HSF1 remains inactive through its association with HSP90. However, upon exposure to stress, HSF1 dissociates from HSP90, becomes activated, and forms nuclear condensates known as nuclear stress bodies. These condensates play a critical role in the expression of chaperone genes.^637^

HSP90 is often overexpressed in various cancers, including breast and lung cancer, where it significantly contributes to the survival and proliferation of cancer cells by stabilizing oncogenic proteins.^638^ STA9090, an HSP90 inhibitor, exerts its anti-cancer effects by promoting the degradation of these oncogenic proteins, thereby disrupting their stability and leading to the condensation of HSF1. Additionally, MG132, a proteasome inhibitor that induces cellular stress by blocking protein degradation, also activates HSF1, resulting in the formation of nuclear condensates.^639^

However, the transient increase in chaperone gene expression through HSF1 condensate formation may primarily serve as a compensatory mechanism to protect cells, rather than directly inhibiting cancer proliferation. Notably, when HSF1 condensates transition to a gel-like state under sustained stress, this may reduce their transcriptional activity and promote apoptosis through complex interactions. Therefore, further investigation into the relationship between HSP90 and HSF1 condensates is warranted.^640^

By modulating or influencing condensates, these compounds hold significant promise for the development of novel therapeutics for related diseases. To achieve this, it is crucial to elucidate the mechanisms underlying condensate formation and their association with disease, necessitating further research into the processes involved.^583^ Additionally, given that the roles and regulatory strategies of condensates may differ across various diseases, a clear classification framework is essential.

### Condensate-modifying (c-mods) therapeutics

**Fig. 11:**
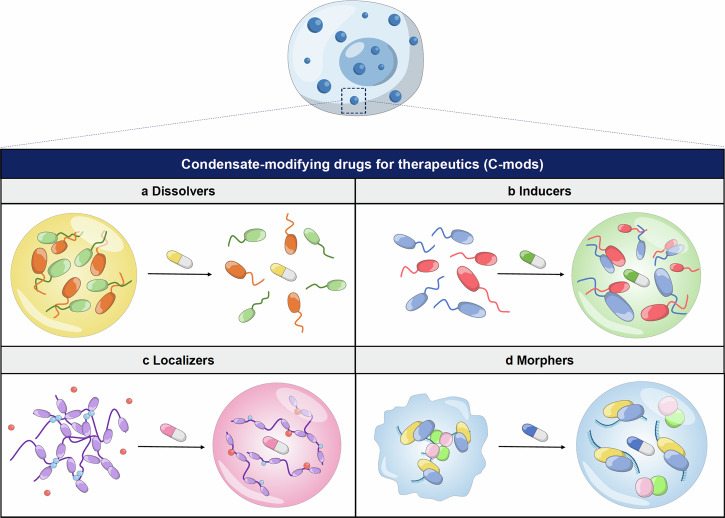
Therapeutic strategies condensate-modifying (C-mod) for therapeutics. **a** The dissolver c-mods disassemble or prevent the formation of pathological condensates, thereby alleviating disease-related aggregation. **b** The inducer c-mods promote the formation of condensates to sequester pathological factors, which can increase the rate of beneficial biochemical reactions. **c** The localizer c-mods correct or restore the spatial localization of specific molecules without significantly disrupting the overall integrity of the condensate. **d** The morpher c-mods alter the morphology and material properties of condensates by modifying the microenvironment, thereby affecting their function and potentially restoring normal cellular process

A range of condensate-modifying drugs (C-mods) is currently being identified, with efforts focused on categorizing them into solubilizers, inducers, localizers, and morphers.^583,641^ This classification, based on the specific mechanisms through which these compounds modify condensates, is important for understanding their modes of action, optimizing therapeutic strategies, and guiding the development of targeted treatments for various diseases. For instance, dissolvers are designed to dissolve condensates, destabilizing pathological aggregates (Fig. 11a). In neurodegenerative diseases such as ALS, proteins like TDP-43 can form stress granules that contribute to pathology.^642^ Solubilizers target these pathological condensates, promoting their disassembly or dissolution. For example, Mitoxantrone is an example of a C-mod solubilizer that has been shown to dissolve persistent TDP-43 condensates.^626^ Mitoxantrone can bind to nucleic acids and potentially interact with IDRs of proteins, such as TDP-43. This interaction can lead to the dissolution of SGs that contain TDP-43. By disrupting the interactions that stabilize these pathological condensates, mitoxantrone can help prevent or reverse the formation of toxic aggregates associated with neurodegenerative diseases like ALS and FTD.^626^

Inducer C-mods are known to promote the formation of beneficial condensates that enhance cellular functions (Fig. 11b). For instance, BI-3802 induces the aggregation of the oncogenic protein BCL6, resulting in its ubiquitination and subsequent degradation.^643^ Although BI-3802’s induction of a helical filament structure in BCL6 and its polymerization may not fit the traditional definition of biomolecular condensates, this mechanism is effective in disrupting harmful protein complexes. By facilitating the degradation of oncogenic proteins through the cell’s own machinery, this approach redirects cellular pathways toward therapeutic outcomes.^583^ These mechanisms can accelerate biochemical reactions through the formation of condensates and, via interconnected pathways, efficiently eliminate causative molecules, thereby contributing to the maintenance of cellular homeostasis.

Localizer C-mods adjust the spatial configuration of biomolecules within condensates to optimize their activity without disrupting overall structure (Fig. 11c). In the context of non-small cell lung cancer, Crizotinib has been shown to impact the behavior of EML4-ALK condensates by influencing their spatial organization. This modulation helps to prevent the aberrant activation of signaling pathways that contribute to drug resistance. By fine-tuning the arrangement of condensate components, Crizotinib enhances the effectiveness of treatment and helps to minimize potential side effects.^644^ This highlights the need for developing localizers that can inhibit the repositioning of condensate components, thereby overcoming the resistance mechanisms associated with Crizotinib. By strategically targeting these interactions, localizers may offer a promising avenue for enhancing therapeutic efficacy in drug-resistant lung cancer.

Morphers play a pivotal role in altering the conditions of a condensate, thereby influencing its function and molecular behavior (Fig. 11d). For instance, by modifying the material properties of RSV condensates, it is possible to inactivate transcription factors and inhibit viral replication. This strategy introduces a novel approach to adjusting the physical properties of condensates, such as viscosity and phase behavior, to restore their proper function.^645^ Despite these advancements, there remains a significant gap in our understanding of the underlying mechanisms, and we lack comprehensive baseline studies needed to effectively categorize compounds that interact with condensates. Continued research into these connections is essential. Nevertheless, we believe that advancing this strategy is crucial for achieving therapeutic success. It goes beyond merely eliminating or creating condensates; rather, it emphasizes attaining the desired pharmacological effect. A meticulous and systematic approach is vital for developing precise treatment strategies tailored to specific diseases, ensuring effective therapeutic outcomes. We summarized the small molecules regulated condensate and associated diseases in Table 8.

**Table 8:** Condensate and associated diseases regulated by small molecules

Disease	Small molecule	Condensate components	Modifying activity	C-mod	Ref.
Cancer	Cisplatin	MED1	Cisplatin accumulates in the MED1 condensate which leads to disruption of super enhancer-based transcriptional activity, resulting in increased structural instability	Dissolver	^575^
	JQ1		JQ1 inhibits BRD4 and increases the structural instability of the MED1 condensate.		^575^
	EPI-001		EPI-001 inhibits the androgen receptor and reduces the formation of AR droplets, interfering with the interaction between AR and MED1.		^582^
	THZ1		THZ1 inhibits hyperphosphorylation and disrupts the interaction between MED1 and the androgen receptor, resulting in reduced expression of AR target genes.		^586^
	BAY249716 / BAY1892005	p53	BAY 249716 and BAY 1892005 regulate the aggregation of modified p53, dissolving aggregates formed from structurally unstable p53 variants.		^591^
	SI-2	SRC3	SI-2 interferes with the interaction between NSD2 and SRC-3, inhibiting SRC-3-mediated phase separation of the liquid-liquid phase, a potential strategy to overcome drug resistance.		^598^
	Oxaliplatin	NPM1	Oxaliplatin interferes with the function of nucleoli, affecting NPM1, a key protein in the nucleus, ultimately leading to cell death.		^601^
ALS	Mitoxantrone	TDP-43	Mitoxantrone has the potential to reduce the progression of ALS by interfering with the formation of aggregates of the RNA-binding protein TDP-43.		^611^
AML	Avrainvillamide	NPM1	Avrainvillamide promotes the nuclear retention of NPMc+ and simultaneously decreases the expression levels of CRM1 and FLT, increasing nuclear localization.	Localizer	^619^
Stress	MG132	HSF1	MG132 induces cellular stress by blocking protein degradation, and this stress activates HSF1 to form nuclear condensates	Inducer	^624^
	STA9090		STA9090 causes cellular stress by promoting the degradation of oncogenic proteins, and this stress promotes the condensation of HSF1 to form nuclear condensates.		

*MED1* mediator complex subunit 1, *SRC3* Steroid receptor coactivator 3, *NPM1* nucleophosmin 1, *ALS* amyotrophic lateral sclerosis, *TDP-43* Transactive response DNA-binding protein 43, *AML* acute myeloid leukemia, *HSF1* heat shock factor 1

### Challenges and future directions in therapeutic targeting of condensates

Understanding the roles of the components that form biomolecular condensates and identifying small molecules that can modulate these structures is crucial for advancing our comprehension of cellular processes and developing novel therapeutic strategies. Translating these findings into effective treatments involves multiple considerations.^583,641^ However, advancements in computational and experimental methodologies have increasingly enhanced the feasibility of developing specific small molecules for therapeutic use.

One of the primary challenges in this area is distinguishing between physiological condensates, which are necessary for normal cellular function, and pathological condensates, which contribute to disease. To effectively target pathological condensates, it is essential to identify measurable biophysical properties that differentiate them from physiological ones.^29^ This distinction is vital for developing therapies that selectively disrupt harmful condensates while preserving those necessary for cellular homeostasis. Recent advances in High-throughput screening (HTS) and advanced imaging technologies have made this differentiation increasingly possible, marking a significant step toward more precise and effective therapeutic interventions.^646,647^ HTS platforms have increasingly incorporated bioinformatics tools to identify proteins involved in LLPS. By leveraging cutting-edge algorithms such as AlphaFold for protein structure prediction based on sequences, and specialized tools like FuzDrop and PhasePred for assessing phase separation potential, these platforms can rapidly sift through extensive protein datasets to identify candidates with LLPS characteristics. These candidate proteins are further validated through transcriptional data sourced from comprehensive databases such as TCGA, or by analyzing patient cohort samples and cellular models, confirming their roles in various disease contexts.^646^ Additionally, integrative approaches such as proteomics are utilized to identify LLPS proteins across the entire proteome. Techniques like density gradient ultracentrifugation coupled with quantitative mass spectrometry enable the detection of endogenous proteins that form biomolecular condensates under specific conditions of phase transition. This allows for the identification of proteins relevant in both physiological and pathological states, thereby expanding our understanding of LLPS mechanisms and uncovering potential therapeutic targets.^279^ Therefore, HTS-driven identification of LLPS proteins not only relies on the known properties of biomolecular condensates but also harnesses vast database resources for predictive accuracy. Subsequent experimental validation of these predictions offers profound insights into the intricate networks that regulate phase separation, paving the way for novel therapeutic strategies.

A particularly promising avenue in LLPS research is the development of small molecules that can modulate phase separation. These molecules hold the potential to either stabilize or dissolve condensates, depending on therapeutic needs.^648,649^ Achieving this requires a thorough understanding of the unique properties of condensates and the molecular interactions that govern their formation and stability. Currently, small molecules are classified based on their effects on condensates, but a disease-oriented classification system, categorizing small molecules based on their applicability to specific diseases, would better guide drug development.^583,584^

Molecular dynamics (MD) simulations are a powerful tool for studying biomolecular condensates at the atomic level. These simulations provide detailed insights into the complex molecular interactions that govern how condensates separate and aggregate. MD simulations are critical for elucidating how small molecules interact with condensates and identifying candidates that can effectively modulate these structures in the context of therapeutic development.^650,651^ For example, computational tools revealed that AIM4 formed a stable complex with specific regions of TDP-43 in studies targeting the protein TDP-43.^652^ With the availability of open-source programs such as GROMACS, LAMMPS, OpenMM, NAMD, and Desmond, researchers can choose the most appropriate tools based on system size, available computational resources, and specific simulation goals.^653^ The utility of MD simulations depends heavily on the integration of experimental data, which is critical for the ongoing validation and refinement of computational models.^654^ Therefore, advancing experimental techniques, such as high-resolution imaging and biophysical analysis, is essential for improving the predictive power of MD simulations and making them more suitable for therapeutic strategies.

Artificial intelligence (AI) is proving to be an invaluable tool for analyzing complex small molecule/protein interactions and predicting their effects on LLPS, thereby improving our ability to understand and target biomolecular condensates.^655^ Models such as Convolutional Atomic Neural Networks (CNN), Grid-based Convolutional Neural Networks (TGrid-CNN), and Graph Convolutional Networks (TGraph-GCN) can process structural data at the atomic level with remarkable precision, providing critical insights for drug development.^656–658^ These insights are critical to the design of new therapies that specifically target condensates. However, the accuracy of these AI predictions is highly dependent on the quality and quantity of experimental data used for model training and validation.^659^ Rigorous experimental validation is essential to effectively translate AI predictions into real applications.^660^ In this regard, in vitro models provide a controlled environment in which the interactions predicted by AI can be tested in a simplified biological context. To assess the therapeutic potential and safety of these interactions in the complex environment of living organisms, in vivo models are essential. Furthermore, the translation of preclinical findings to human applications requires careful attention to interspecies differences in experimental design and data interpretation. Ultimately, the establishment of clinical relevance of target condensates is critical to bridge the gap between basic research and successful clinical outcomes, thereby ensuring that AI-driven discoveries are translated into effective therapeutic interventions.

Collaboration among biologists, chemists, data scientists, and clinicians is critical to advancing the field of targeted condensates. This interdisciplinary approach is essential to address the complexity of condensate dynamics involving intricate biological processes, elaborate chemical modifications, and advanced data analysis techniques. By utilizing the unique expertise of each field, we can develop more effective therapeutic strategies, improve the accuracy of predictive models, and facilitate the successful translation of research into safe and effective clinical applications. Such collaborative efforts are essential to effectively address the challenges associated with targeting biomolecular condensates to treat disease.

## References

[1] Zhu, L. & Brangwynne, C. P. Nuclear bodies: the emerging biophysics of nucleasmic phases. *Curr. Opin. Cell Biol.* **34**, 23–30 (2015). 25942753 10.1016/j.ceb.2015.04.003 PMC5562147

[2] Banani, S. F. et al. Compositional control of phase-separated cellular bodies. *Cell* **166**, 651–663 (2016). 27374333 10.1016/j.cell.2016.06.010 PMC4967043

[3] Feric, M. et al. Coexisting liquid phases underlie nucleolar subcompartments. *Cell* **165**, 1686–1697 (2016). 27212236 10.1016/j.cell.2016.04.047 PMC5127388

[4] Hirose, T., Ninomiya, K., Nakagawa, S. & Yamazaki, T. A guide to membraneless organelles and their various roles in gene regulation. *Nat. Rev. Mol. Cell Biol.* **24**, 288–304 (2023). 36424481 10.1038/s41580-022-00558-8

[5] Banani, S. F., Lee, H. O., Hyman, A. A. & Rosen, M. K. Biomolecular condensates: organizers of cellular biochemistry. *Nat. Rev. Mol. Cell Biol.* **18**, 285–298 (2017). 28225081 10.1038/nrm.2017.7 PMC7434221

[6] Lin, Y., Protter, D. S., Rosen, M. K. & Parker, R. Formation and maturation of phase-separated liquid droplets by RNA-binding proteins. *Mol. Cell* **60**, 208–219 (2015). 26412307 10.1016/j.molcel.2015.08.018 PMC4609299

[7] Alberti, S. & Hyman, A. A. Biomolecular condensates at the nexus of cellular stress, protein aggregation disease and ageing. *Nat. Rev. Mol. Cell Biol.* **22**, 196–213 (2021). 33510441 10.1038/s41580-020-00326-6

[8] Niu, X. et al. Biomolecular condensates: formation mechanisms, biological functions, and therapeutic targets. *MedComm* **4**, e223 (2023). 36875159 10.1002/mco2.223 PMC9974629

[9] Zhou, C. The molecular and functional interaction between membrane-bound organelles and membrane-less condensates. *Front. Cell Dev. Biol.* **10**, 896305 (2022). 35547815 10.3389/fcell.2022.896305 PMC9081682

[10] Zhao, Y. G. & Zhang, H. Phase separation in membrane biology: the interplay between membrane-bound organelles and membraneless condensates. *Dev. Cell* **55**, 30–44 (2020). 32726575 10.1016/j.devcel.2020.06.033

[11] Gomes, E. & Shorter, J. The molecular language of membraneless organelles. *J. Biol. Chem.* **294**, 7115–7127 (2019). 30045872 10.1074/jbc.TM118.001192 PMC6509512

[12] Wilson, E. B. The structure of protoplasm. *Science* **10**, 33–45 (1899). 17829686 10.1126/science.10.237.33

[13] Brangwynne, C. P. et al. Germline P granules are liquid droplets that localize by controlled dissolution/condensation. *Science* **324**, 1729–1732 (2009). 19460965 10.1126/science.1172046

[14] Brangwynne, C. P., Mitchison, T. J. & Hyman, A. A. Active liquid-like behavior of nucleoli determines their size and shape in Xenopus laevis oocytes. *Proc. Natl Acad. Sci. USA* **108**, 4334–4339 (2011). 21368180 10.1073/pnas.1017150108 PMC3060270

[15] Kato, M. et al. Cell-free formation of RNA granules: low complexity sequence domains form dynamic fibers within hydrogels. *Cell* **149**, 753–767 (2012). 22579281 10.1016/j.cell.2012.04.017 PMC6347373

[16] Henis, Y. I., Rotblat, B. & Kloog, Y. FRAP beam-size analysis to measure palmitoylation-dependent membrane association dynamics and microdomain partitioning of Ras proteins. *Methods* **40**, 183–190 (2006). 17012031 10.1016/j.ymeth.2006.02.003

[17] Molliex, A. et al. Phase separation by low complexity domains promotes stress granule assembly and drives pathological fibrillization. *Cell* **163**, 123–133 (2015). 26406374 10.1016/j.cell.2015.09.015 PMC5149108

[18] Nott, T. J. et al. Phase transition of a disordered Nuage protein generates environmentally responsive membraneless organelles. *Mol. Cell* **57**, 936–947 (2015). 25747659 10.1016/j.molcel.2015.01.013 PMC4352761

[19] Lu, Y. et al. Phase separation of TAZ compartmentalizes the transcription machinery to promote gene expression. *Nat. Cell Biol.* **22**, 453–464 (2020). 32203417 10.1038/s41556-020-0485-0 PMC11044910

[20] Shao, Y. et al. A chaperone-like function of FUS ensures TAZ condensate dynamics and transcriptional activation. *Nat. Cell Biol.* **26**, 86–99 (2024). 38172614 10.1038/s41556-023-01309-3

[21] Shin, Y. et al. Spatiotemporal control of intracellular phase transitions using light-activated optoDroplets. *Cell* **168**, 159–171.e114 (2017). 28041848 10.1016/j.cell.2016.11.054 PMC5562165

[22] Rademacher, A., Erdel, F., Weinmann, R. & Rippe, K. Assessing the phase separation propensity of proteins in living cells through optodroplet formation. *Methods Mol. Biol.* **2563**, 395–411 (2023). 36227485 10.1007/978-1-0716-2663-4_20

[23] Paci, G. & Lemke, E. A. Shining a light on phase separation in the cell. *Cell* **168**, 11–13 (2017). 28086083 10.1016/j.cell.2016.12.018

[24] Taslimi, A. et al. An optimized optogenetic clustering tool for probing protein interaction and function. *Nat. Commun.* **5**, 4925 (2014). 25233328 10.1038/ncomms5925 PMC4170572

[25] Khamo, J. S., Krishnamurthy, V. V., Sharum, S. R., Mondal, P. & Zhang, K. Applications of optobiology in intact cells and multicellular organisms. *J. Mol. Biol.* **429**, 2999–3017 (2017). 28882542 10.1016/j.jmb.2017.08.015

[26] Kim, C. & Shin, Y. An optogenetic toolkit for the control of phase separation in living cells. *Methods Mol. Biol.* **2563**, 383–394 (2023). 36227484 10.1007/978-1-0716-2663-4_19

[27] Abyzov, A. S. & Schmelzer, J. W. Nucleation versus spinodal decomposition in confined binary solutions. *J. Chem. Phys.* **127**, 114504 (2007). 17887854 10.1063/1.2774989

[28] Shimobayashi, S. F., Ronceray, P., Sanders, D. W., Haataja, M. P. & Brangwynne, C. P. Nucleation landscape of biomolecular condensates. *Nature* **599**, 503–506 (2021). 34552246 10.1038/s41586-021-03905-5

[29] Alberti, S., Gladfelter, A. & Mittag, T. Considerations and challenges in studying liquid-liquid phase separation and biomolecular condensates. *Cell* **176**, 419–434 (2019). 30682370 10.1016/j.cell.2018.12.035 PMC6445271

[30] Gibson, B. A. et al. Organization of chromatin by intrinsic and regulated phase separation. *Cell* **179**, 470–484.e421 (2019). 31543265 10.1016/j.cell.2019.08.037 PMC6778041

[31] Wang, L. et al. Histone modifications regulate chromatin compartmentalization by contributing to a phase separation mechanism. *Mol. Cell* **76**, 646–659.e646 (2019). 31543422 10.1016/j.molcel.2019.08.019

[32] Shvedunova, M. & Akhtar, A. Modulation of cellular processes by histone and non-histone protein acetylation. *Nat. Rev. Mol. Cell Biol.* **23**, 329–349 (2022). 35042977 10.1038/s41580-021-00441-y

[33] Levone, B. R. et al. FUS-dependent liquid-liquid phase separation is important for DNA repair initiation. *J. Cell Biol.* **220**, e202008030 (2021). 33704371 10.1083/jcb.202008030 PMC7953258

[34] Oshidari, R. et al. DNA repair by Rad52 liquid droplets. *Nat. Commun.* **11**, 695 (2020). 32019927 10.1038/s41467-020-14546-z PMC7000754

[35] Wang, Y. L. et al. Liquid-liquid phase separation in DNA double-strand breaks repair. *Cell Death Dis.* **14**, 746 (2023). 37968256 10.1038/s41419-023-06267-0 PMC10651886

[36] Tong, X. et al. Liquid-liquid phase separation in tumor biology. *Signal Transduct. Target. Ther.* **7**, 221 (2022). 35803926 10.1038/s41392-022-01076-x PMC9270353

[37] Xiao, Q., McAtee, C. K. & Su, X. Phase separation in immune signalling. *Nat. Rev. Immunol.* **22**, 188–199 (2022). 34230650 10.1038/s41577-021-00572-5 PMC9674404

[38] Zbinden, A., Pérez-Berlanga, M., De Rossi, P. & Polymenidou, M. Phase separation and neurodegenerative diseases: a disturbance in the force. *Dev. Cell* **55**, 45–68 (2020). 33049211 10.1016/j.devcel.2020.09.014

[39] Taniue, K. & Akimitsu, N. Aberrant phase separation and cancer. *FEBS J.* **289**, 17–39 (2022). 33583140 10.1111/febs.15765

[40] Mehta, S. & Zhang, J. Liquid-liquid phase separation drives cellular function and dysfunction in cancer. *Nat. Rev. Cancer* **22**, 239–252 (2022). 35149762 10.1038/s41568-022-00444-7 PMC10036213

[41] Boija, A., Klein, I. A. & Young, R. A. Biomolecular condensates and cancer. *Cancer Cell* **39**, 174–192 (2021). 33417833 10.1016/j.ccell.2020.12.003 PMC8721577

[42] Cai, D., Liu, Z. & Lippincott-Schwartz, J. Biomolecular condensates and their links to cancer progression. *Trends Biochem. Sci.* **46**, 535–549 (2021). 33579564 10.1016/j.tibs.2021.01.002

[43] Jiang, S., Fagman, J. B., Chen, C., Alberti, S. & Liu, B. Protein phase separation and its role in tumorigenesis. *Elife* **9**, e60264 (2020). 33138914 10.7554/eLife.60264 PMC7609067

[44] Ambadipudi, S., Biernat, J., Riedel, D., Mandelkow, E. & Zweckstetter, M. Liquid-liquid phase separation of the microtubule-binding repeats of the Alzheimer-related protein Tau. *Nat. Commun.* **8**, 275 (2017). 28819146 10.1038/s41467-017-00480-0 PMC5561136

[45] Mukherjee, S. et al. Liquid-liquid phase separation of α-synuclein: a new mechanistic insight for α-synuclein aggregation associated with Parkinson’s disease pathogenesis. *J. Mol. Biol.* **435**, 167713 (2023). 35787838 10.1016/j.jmb.2022.167713

[46] Hurtle, B. T., Xie, L. & Donnelly, C. J. Disrupting pathologic phase transitions in neurodegeneration. *J. Clin. Investig.* **133**, e168549 (2023). 37395272 10.1172/JCI168549 PMC10313377

[47] Wang, B. et al. Liquid-liquid phase separation in human health and diseases. *Signal Transduct. Target. Ther.* **6**, 290 (2021). 34334791 10.1038/s41392-021-00678-1 PMC8326283

[48] Vernon, R. M. et al. Pi-Pi contacts are an overlooked protein feature relevant to phase separation. *Elife* **7**, e31486 (2018). 29424691 10.7554/eLife.31486 PMC5847340

[49] Sesé-Sansa, E., Liao, G. J., Levis, D., Pagonabarraga, I. & Klapp, S. H. L. Impact of dipole-dipole interactions on motility-induced phase separation. *Soft Matter* **18**, 5388–5401 (2022). 35797661 10.1039/d2sm00385f

[50] Dutagaci, B. et al. Charge-driven condensation of RNA and proteins suggests broad role of phase separation in cytoplasmic environments. *Elife* **10**, e64004 (2021). 33496264 10.7554/eLife.64004 PMC7877912

[51] Lin, Y. H., Forman-Kay, J. D. & Chan, H. S. Theories for sequence-dependent phase behaviors of biomolecular condensates. *Biochemistry* **57**, 2499–2508 (2018). 29509422 10.1021/acs.biochem.8b00058

[52] Ibrahim, A. Y. et al. Intrinsically disordered regions that drive phase separation form a robustly distinct protein class. *J. Biol. Chem.* **299**, 102801 (2023). 36528065 10.1016/j.jbc.2022.102801 PMC9860499

[53] De Sancho, D. Phase separation in amino acid mixtures is governed by composition. *Biophys. J.* **121**, 4119–4127 (2022). 36181270 10.1016/j.bpj.2022.09.031 PMC9675019

[54] Lin, Y., Currie, S. L. & Rosen, M. K. Intrinsically disordered sequences enable modulation of protein phase separation through distributed tyrosine motifs. *J. Biol. Chem.* **292**, 19110–19120 (2017). 28924037 10.1074/jbc.M117.800466 PMC5704491

[55] Ranganathan, S. & Shakhnovich, E. I. Dynamic metastable long-living droplets formed by sticker-spacer proteins. *Elife* **9**, e56159 (2020). 32484438 10.7554/eLife.56159 PMC7360371

[56] Wang, J. et al. A molecular grammar governing the driving forces for phase separation of prion-like RNA binding proteins. *Cell* **174**, 688–699.e616 (2018). 29961577 10.1016/j.cell.2018.06.006 PMC6063760

[57] Boyd-Shiwarski, C. R. et al. WNK kinases sense molecular crowding and rescue cell volume via phase separation. *Cell* **185**, 4488–4506.e4420 (2022). 36318922 10.1016/j.cell.2022.09.042 PMC9699283

[58] Yang, Y., Jones, H. B., Dao, T. P. & Castañeda, C. A. Single amino acid substitutions in stickers, but not spacers, substantially alter UBQLN2 phase transitions and dense phase material properties. *J. Phys. Chem. B* **123**, 3618–3629 (2019). 30925840 10.1021/acs.jpcb.9b01024

[59] Alshareedah, I., Moosa, M. M., Pham, M., Potoyan, D. A. & Banerjee, P. R. Programmable viscoelasticity in protein-RNA condensates with disordered sticker-spacer polypeptides. *Nat. Commun.* **12**, 6620 (2021). 34785657 10.1038/s41467-021-26733-7 PMC8595643

[60] Ginell, G. M. & Holehouse, A. S. An introduction to the stickers-and-spacers framework as applied to biomolecular condensates. *Methods Mol. Biol.* **2563**, 95–116 (2023). 36227469 10.1007/978-1-0716-2663-4_4

[61] Xiong, Q., Chen, Z. & Ge, F. Proteomic analysis of post translational modifications in cyanobacteria. *J. Proteom.* **134**, 57–64 (2016). 10.1016/j.jprot.2015.07.037 26254007

[62] Peng, T., Das, T., Ding, K. & Hang, H. C. Functional analysis of protein post-translational modifications using genetic codon expansion. *Protein Sci.* **32**, e4618 (2023). 36883310 10.1002/pro.4618 PMC10031814

[63] Sonenberg, N. & Hinnebusch, A. G. Regulation of translation initiation in eukaryotes: mechanisms and biological targets. *Cell* **136**, 731–745 (2009). 19239892 10.1016/j.cell.2009.01.042 PMC3610329

[64] Li, J. et al. Post-translational modifications in liquid-liquid phase separation: a comprehensive review. *Mol. Biomed.* **3**, 13 (2022). 35543798 10.1186/s43556-022-00075-2 PMC9092326

[65] Wang, A. et al. A single N-terminal phosphomimic disrupts TDP-43 polymerization, phase separation, and RNA splicing. *EMBO J.* **37**, e97452 (2018). 29438978 10.15252/embj.201797452 PMC5830921

[66] Jin, F. & Gräter, F. How multisite phosphorylation impacts the conformations of intrinsically disordered proteins. *PLoS Comput. Biol.* **17**, e1008939 (2021). 33945530 10.1371/journal.pcbi.1008939 PMC8148376

[67] Monahan, Z. et al. Phosphorylation of the FUS low-complexity domain disrupts phase separation, aggregation, and toxicity. *EMBO J.* **36**, 2951–2967 (2017). 28790177 10.15252/embj.201696394 PMC5641905

[68] Brady, J. P. et al. Structural and hydrodynamic properties of an intrinsically disordered region of a germ cell-specific protein on phase separation. *Proc. Natl Acad. Sci. USA* **114**, E8194–e8203 (2017). 28894006 10.1073/pnas.1706197114 PMC5625912

[69] Hofweber, M. et al. Phase separation of FUS is suppressed by its nuclear import receptor and arginine methylation. *Cell* **173**, 706–719.e713 (2018). 29677514 10.1016/j.cell.2018.03.004

[70] Qamar, S. et al. FUS phase separation is modulated by a molecular chaperone and methylation of arginine cation-π interactions. *Cell* **173**, 720–734.e715 (2018). 29677515 10.1016/j.cell.2018.03.056 PMC5927716

[71] Ryan, V. H. et al. Mechanistic view of hnRNPA2 low-complexity domain structure, interactions, and phase separation altered by mutation and arginine methylation. *Mol. Cell* **69**, 465–479.e467 (2018). 29358076 10.1016/j.molcel.2017.12.022 PMC5801700

[72] Matsumoto, K. et al. PRMT1 is required for RAP55 to localize to processing bodies. *RNA Biol.* **9**, 610–623 (2012). 22614839 10.4161/rna.19527

[73] Paik, W. K. & Kim, S. Protein methylation: chemical, enzymological, and biological significance. *Adv. Enzymol. Relat. Areas Mol. Biol.* **42**, 227–286 (1975). 1093364 10.1002/9780470122877.ch5

[74] Arribas-Layton, M., Dennis, J., Bennett, E. J., Damgaard, C. K. & Lykke-Andersen, J. The C-terminal RGG domain of human Lsm4 promotes processing body formation stimulated by arginine dimethylation. *Mol. Cell. Biol.* **36**, 2226–2235 (2016). 27247266 10.1128/MCB.01102-15 PMC4985932

[75] Bouchard, J. J. et al. Cancer mutations of the tumor suppressor SPOP disrupt the formation of active, phase-separated compartments. *Mol. Cell* **72**, 19–36.e18 (2018). 30244836 10.1016/j.molcel.2018.08.027 PMC6179159

[76] Kwon, J. E. et al. BTB domain-containing speckle-type POZ protein (SPOP) serves as an adaptor of Daxx for ubiquitination by Cul3-based ubiquitin ligase. *J. Biol. Chem.* **281**, 12664–12672 (2006). 16524876 10.1074/jbc.M600204200

[77] Hernández-Muñoz, I. et al. Stable X chromosome inactivation involves the PRC1 Polycomb complex and requires histone MACROH2A1 and the CULLIN3/SPOP ubiquitin E3 ligase. *Proc. Natl Acad. Sci. USA* **102**, 7635–7640 (2005). 15897469 10.1073/pnas.0408918102 PMC1140410

[78] Liu, Y., Feng, W., Wang, Y. & Wu, B. Crosstalk between protein post-translational modifications and phase separation. *Cell Commun. Signal.* **22**, 110 (2024). 38347544 10.1186/s12964-023-01380-1 PMC10860296

[79] Banerjee, P. R., Milin, A. N., Moosa, M. M., Onuchic, P. L. & Deniz, A. A. Reentrant phase transition drives dynamic substructure formation in ribonucleoprotein droplets. *Angew. Chem. Int. Ed. Engl.* **56**, 11354–11359 (2017). 28556382 10.1002/anie.201703191 PMC5647147

[80] Lin, Y. & Fang, X. Phase separation in RNA biology. *J. Genet. Genom.* **48**, 872–880 (2021). 10.1016/j.jgg.2021.07.012 34371110

[81] Maharana, S. et al. RNA buffers the phase separation behavior of prion-like RNA binding proteins. *Science* **360**, 918–921 (2018). 29650702 10.1126/science.aar7366 PMC6091854

[82] Langdon, E. M. et al. mRNA structure determines specificity of a polyQ-driven phase separation. *Science* **360**, 922–927 (2018). 29650703 10.1126/science.aar7432 PMC6192030

[83] Zhang, Y. et al. G-quadruplex structures trigger RNA phase separation. *Nucleic Acids Res* **47**, 11746–11754 (2019). 31722410 10.1093/nar/gkz978 PMC7145655

[84] Wiedner, H. J. & Giudice, J. It’s not just a phase: function and characteristics of RNA-binding proteins in phase separation. *Nat. Struct. Mol. Biol.* **28**, 465–473 (2021). 34099940 10.1038/s41594-021-00601-w PMC8787349

[85] Poudyal, R. R., Sieg, J. P., Portz, B., Keating, C. D. & Bevilacqua, P. C. RNA sequence and structure control assembly and function of RNA condensates. *RNA* **27**, 1589–1601 (2021). 34551999 10.1261/rna.078875.121 PMC8594466

[86] Luo, J. et al. LncRNAs: architectural scaffolds or more potential roles in phase separation. *Front. Genet.* **12**, 626234 (2021). 33868368 10.3389/fgene.2021.626234 PMC8044363

[87] Somasundaram, K., Gupta, B., Jain, N. & Jana, S. LncRNAs divide and rule: the master regulators of phase separation. *Front. Genet.* **13**, 930792 (2022). 36035193 10.3389/fgene.2022.930792 PMC9399341

[88] Hirose, T. et al. NEAT1 long noncoding RNA regulates transcription via protein sequestration within subnuclear bodies. *Mol. Biol. Cell* **25**, 169–183 (2014). 24173718 10.1091/mbc.E13-09-0558 PMC3873887

[89] Markaki, Y. et al. Xist nucleates local protein gradients to propagate silencing across the X chromosome. *Cell* **184**, 6174–6192.e6132 (2021). 34813726 10.1016/j.cell.2021.10.022 PMC8671326

[90] Hentze, M. W., Castello, A., Schwarzl, T. & Preiss, T. A brave new world of RNA-binding proteins. *Nat. Rev. Mol. Cell Biol.* **19**, 327–341 (2018). 29339797 10.1038/nrm.2017.130

[91] Li, W., Deng, X. & Chen, J. RNA-binding proteins in regulating mRNA stability and translation: roles and mechanisms in cancer. *Semin. Cancer Biol.* **86**, 664–677 (2022). 35381329 10.1016/j.semcancer.2022.03.025 PMC9526761

[92] Gotor, N. L. et al. RNA-binding and prion domains: the Yin and Yang of phase separation. *Nucleic Acids Res* **48**, 9491–9504 (2020). 32857852 10.1093/nar/gkaa681 PMC7515694

[93] Malinovska, L., Kroschwald, S. & Alberti, S. Protein disorder, prion propensities, and self-organizing macromolecular collectives. *Biochim. Biophys. Acta* **1834**, 918–931 (2013). 23328411 10.1016/j.bbapap.2013.01.003

[94] Martin, E. W. et al. Valence and patterning of aromatic residues determine the phase behavior of prion-like domains. *Science* **367**, 694–699 (2020). 32029630 10.1126/science.aaw8653 PMC7297187

[95] March, Z. M., King, O. D. & Shorter, J. Prion-like domains as epigenetic regulators, scaffolds for subcellular organization, and drivers of neurodegenerative disease. *Brain Res* **1647**, 9–18 (2016). 26996412 10.1016/j.brainres.2016.02.037 PMC5003744

[96] Chong, P. A., Vernon, R. M. & Forman-Kay, J. D. RGG/RG motif regions in RNA binding and phase separation. *J. Mol. Biol.* **430**, 4650–4665 (2018). 29913160 10.1016/j.jmb.2018.06.014

[97] Schuster, B. S. et al. Controllable protein phase separation and modular recruitment to form responsive membraneless organelles. *Nat. Commun.* **9**, 2985 (2018). 30061688 10.1038/s41467-018-05403-1 PMC6065366

[98] Maris, C., Dominguez, C. & Allain, F. H. The RNA recognition motif, a plastic RNA-binding platform to regulate post-transcriptional gene expression. *FEBS J.* **272**, 2118–2131 (2005). 15853797 10.1111/j.1742-4658.2005.04653.x

[99] Mohanty, P. et al. Principles governing the phase separation of multidomain proteins. *Biochemistry* **61**, 2443–2455 (2022). 35802394 10.1021/acs.biochem.2c00210 PMC9669140

[100] Guillén-Boixet, J. et al. RNA-induced conformational switching and clustering of G3BP drive stress granule assembly by condensation. *Cell* **181**, 346–361.e317 (2020). 32302572 10.1016/j.cell.2020.03.049 PMC7181197

[101] Yamanishi, K., Kumano, W., Terawaki, S. I., Higuchi, Y. & Shibata, N. Head-to-tail complex of Dishevelled and Axin-DIX domains: expression, purification, crystallographic studies and packing analysis. *Protein Pept. Lett.* **26**, 792–797 (2019). 31618172 10.2174/0929866526666190425152721

[102] Yamanishi, K. et al. A direct heterotypic interaction between the DIX domains of Dishevelled and Axin mediates signaling to β-catenin. *Sci. Signal.* **12**, eaaw5505 (2019). 31822591 10.1126/scisignal.aaw5505 PMC6923138

[103] Fiedler, M., Mendoza-Topaz, C., Rutherford, T. J., Mieszczanek, J. & Bienz, M. Dishevelled interacts with the DIX domain polymerization interface of Axin to interfere with its function in down-regulating β-catenin. *Proc. Natl Acad. Sci. USA* **108**, 1937–1942 (2011). 21245303 10.1073/pnas.1017063108 PMC3033301

[104] Lamark, T. et al. Interaction codes within the family of mammalian Phox and Bem1p domain-containing proteins. *J. Biol. Chem.* **278**, 34568–34581 (2003). 12813044 10.1074/jbc.M303221200

[105] Wilson, M. I., Gill, D. J., Perisic, O., Quinn, M. T. & Williams, R. L. PB1 domain-mediated heterodimerization in NADPH oxidase and signaling complexes of atypical protein kinase C with Par6 and p62. *Mol. Cell* **12**, 39–50 (2003). 12887891 10.1016/s1097-2765(03)00246-6

[106] Mariotti, L. et al. Tankyrase requires SAM domain-dependent polymerization to support Wnt-β-catenin signaling. *Mol. Cell* **63**, 498–513 (2016). 27494558 10.1016/j.molcel.2016.06.019 PMC4980433

[107] Blackledge, N. P. & Klose, R. J. The molecular principles of gene regulation by Polycomb repressive complexes. *Nat. Rev. Mol. Cell Biol.* **22**, 815–833 (2021). 34400841 10.1038/s41580-021-00398-y PMC7612013

[108] Isono, K. et al. SAM domain polymerization links subnuclear clustering of PRC1 to gene silencing. *Dev. Cell* **26**, 565–577 (2013). 24091011 10.1016/j.devcel.2013.08.016

[109] Fiedler, M. et al. Head-to-tail polymerization by VEL proteins underpins cold-induced Polycomb silencing in flowering control. *Cell Rep.* **41**, 111607 (2022). 36351412 10.1016/j.celrep.2022.111607 PMC7614096

[110] Bienz, M. Head-to-tail polymerization in the assembly of biomolecular condensates. *Cell* **182**, 799–811 (2020). 32822572 10.1016/j.cell.2020.07.037

[111] Eisenberg, D. & Jucker, M. The amyloid state of proteins in human diseases. *Cell* **148**, 1188–1203 (2012). 22424229 10.1016/j.cell.2012.02.022 PMC3353745

[112] Sawaya, M. R. et al. Atomic structures of amyloid cross-beta spines reveal varied steric zippers. *Nature* **447**, 453–457 (2007). 17468747 10.1038/nature05695

[113] Li, J. et al. The RIP1/RIP3 necrosome forms a functional amyloid signaling complex required for programmed necrosis. *Cell* **150**, 339–350 (2012). 22817896 10.1016/j.cell.2012.06.019 PMC3664196

[114] Bienz, M. Signalosome assembly by domains undergoing dynamic head-to-tail polymerization. *Trends Biochem. Sci.* **39**, 487–495 (2014). 25239056 10.1016/j.tibs.2014.08.006

[115] Wu, H. & Fuxreiter, M. The structure and dynamics of higher-order assemblies: amyloids, signalosomes, and granules. *Cell* **165**, 1055–1066 (2016). 27203110 10.1016/j.cell.2016.05.004 PMC4878688

[116] Coussens, N. P. et al. Multipoint binding of the SLP-76 SH2 domain to ADAP is critical for oligomerization of SLP-76 signaling complexes in stimulated T cells. *Mol. Cell Biol.* **33**, 4140–4151 (2013). 23979596 10.1128/MCB.00410-13 PMC3811887

[117] Naskar, A., Nayak, A., Salaikumaran, M. R., Vishal, S. S. & Gopal, P. P. Phase separation and pathologic transitions of RNP condensates in neurons: implications for amyotrophic lateral sclerosis, frontotemporal dementia and other neurodegenerative disorders. *Front. Mol. Neurosci.* **16**, 1242925 (2023). 37720552 10.3389/fnmol.2023.1242925 PMC10502346

[118] Protter, D. S. W. et al. Intrinsically disordered regions can contribute promiscuous interactions to RNP granule assembly. *Cell Rep.* **22**, 1401–1412 (2018). 29425497 10.1016/j.celrep.2018.01.036 PMC5824733

[119] Frey, S., Richter, R. P. & Görlich, D. FG-rich repeats of nuclear pore proteins form a three-dimensional meshwork with hydrogel-like properties. *Science* **314**, 815–817 (2006). 17082456 10.1126/science.1132516

[120] Nag, N., Sasidharan, S., Uversky, V. N., Saudagar, P. & Tripathi, T. Phase separation of FG-nucleoporins in nuclear pore complexes. *Biochim. Biophys. Acta Mol. Cell Res.* **1869**, 119205 (2022). 34995711 10.1016/j.bbamcr.2021.119205

[121] Zilman, A. Aggregation, phase separation and spatial morphologies of the assemblies of FG nucleoporins. *J. Mol. Biol.* **430**, 4730–4740 (2018). 30017917 10.1016/j.jmb.2018.07.011

[122] Dormann, D. FG-nucleoporins caught in the act of liquid-liquid phase separation. *J. Cell Biol.* **219**, e201910211 (2020). 31834369 10.1083/jcb.201910211 PMC7039191

[123] Chu, W. T. & Wang, J. Thermodynamic and sequential characteristics of phase separation and droplet formation for an intrinsically disordered region/protein ensemble. *PLoS Comput. Biol.* **17**, e1008672 (2021). 33684117 10.1371/journal.pcbi.1008672 PMC7939360

[124] Riback, J. A. et al. Stress-triggered phase separation is an adaptive, evolutionarily tuned response. *Cell* **168**, 1028–1040.e1019 (2017). 28283059 10.1016/j.cell.2017.02.027 PMC5401687

[125] Weber, S. C. & Brangwynne, C. P. Inverse size scaling of the nucleolus by a concentration-dependent phase transition. *Curr. Biol.* **25**, 641–646 (2015). 25702583 10.1016/j.cub.2015.01.012 PMC4348177

[126] Berry, J., Weber, S. C., Vaidya, N., Haataja, M. & Brangwynne, C. P. RNA transcription modulates phase transition-driven nuclear body assembly. *Proc. Natl Acad. Sci. USA* **112**, E5237–E5245 (2015). 26351690 10.1073/pnas.1509317112 PMC4586886

[127] Cai, D. et al. Phase separation of YAP reorganizes genome topology for long-term YAP target gene expression. *Nat. Cell Biol.* **21**, 1578–1589 (2019). 31792379 10.1038/s41556-019-0433-z PMC8259329

[128] Liu, Z. et al. Par complex cluster formation mediated by phase separation. *Nat. Commun.* **11**, 2266 (2020). 32385244 10.1038/s41467-020-16135-6 PMC7211019

[129] Wegmann, S. et al. Tau protein liquid-liquid phase separation can initiate tau aggregation. *EMBO J.* **37**, e98049 (2018). 29472250 10.15252/embj.201798049 PMC5881631

[130] Ray, S. et al. α-Synuclein aggregation nucleates through liquid-liquid phase separation. *Nat. Chem.* **12**, 705–716 (2020). 32514159 10.1038/s41557-020-0465-9

[131] Navalkar, A. et al. Prion-like p53 amyloids in cancer. *Biochemistry* **59**, 146–155 (2020). 31603660 10.1021/acs.biochem.9b00796

[132] Levengood, J. D., Peterson, J., Tolbert, B. S. & Roche, J. Thermodynamic stability of hnRNP A1 low complexity domain revealed by high-pressure NMR. *Proteins* **89**, 781–791 (2021). 33550645 10.1002/prot.26058 PMC9122033

[133] Félix, S. S., Laurents, D. V., Oroz, J. & Cabrita, E. J. Fused in sarcoma undergoes cold denaturation: implications for phase separation. *Protein Sci.* **32**, e4521 (2023). 36453011 10.1002/pro.4521 PMC9793971

[134] Iserman, C. et al. Genomic RNA elements drive phase separation of the SARS-CoV-2 nucleocapsid. *Mol. Cell* **80**, 1078–1091.e1076 (2020). 33290746 10.1016/j.molcel.2020.11.041 PMC7691212

[135] Jung, J. H. et al. A prion-like domain in ELF3 functions as a thermosensor in Arabidopsis. *Nature* **585**, 256–260 (2020). 32848244 10.1038/s41586-020-2644-7

[136] Martin, E. W. & Mittag, T. Relationship of sequence and phase separation in protein low-complexity regions. *Biochemistry* **57**, 2478–2487 (2018). 29517898 10.1021/acs.biochem.8b00008 PMC6476794

[137] Dignon, G. L., Zheng, W., Kim, Y. C. & Mittal, J. Temperature-controlled liquid-liquid phase separation of disordered proteins. *ACS Cent. Sci.* **5**, 821–830 (2019). 31139718 10.1021/acscentsci.9b00102 PMC6535772

[138] Adame-Arana, O., Weber, C. A., Zaburdaev, V., Prost, J. & Jülicher, F. Liquid phase separation controlled by pH. Biophys. *J* **119**, 1590–1605 (2020). 10.1016/j.bpj.2020.07.044 PMC7642337 33010236

[139] Díez Pérez, T. et al. Isolation of nucleic acids using liquid-liquid phase separation of pH-sensitive elastin-like polypeptides. *Sci. Rep.* **14**, 10157 (2024). 38698072 10.1038/s41598-024-60648-9 PMC11065875

[140] Baidya, L. & Reddy, G. pH induced switch in the conformational ensemble of intrinsically disordered protein prothymosin-α and its implications for amyloid fibril formation. *J. Phys. Chem. Lett.* **13**, 9589–9598 (2022). 36206480 10.1021/acs.jpclett.2c01972

[141] Santos, J. et al. pH-dependent aggregation in intrinsically disordered proteins is determined by charge and lipophilicity. *Cells* **9**, 145 (2020). 31936201 10.3390/cells9010145 PMC7017033

[142] Huihui, J., Firman, T. & Ghosh, K. Modulating charge patterning and ionic strength as a strategy to induce conformational changes in intrinsically disordered proteins. *J. Chem. Phys.* **149**, 085101 (2018). 30193467 10.1063/1.5037727

[143] Dias, C. L. & Chan, H. S. Pressure-dependent properties of elementary hydrophobic interactions: ramifications for activation properties of protein folding. *J. Phys. Chem. B* **118**, 7488–7509 (2014). 24933471 10.1021/jp501935f

[144] Cinar, H., Cinar, S., Chan, H. S. & Winter, R. Pressure-induced dissolution and reentrant formation of condensed, liquid-liquid phase-separated elastomeric α-elastin. *Chemistry* **24**, 8286–8291 (2018). 29738068 10.1002/chem.201801643

[145] Majumder, S. & Jain, A. Osmotic stress triggers phase separation. *Mol. Cell* **79**, 876–877 (2020). 32946761 10.1016/j.molcel.2020.09.001

[146] Mukherjee, S. & Schäfer, L. V. Thermodynamic forces from protein and water govern condensate formation of an intrinsically disordered protein domain. *Nat. Commun.* **14**, 5892 (2023). 37735186 10.1038/s41467-023-41586-y PMC10514047

[147] Cinar, H. et al. Temperature, hydrostatic pressure, and osmolyte effects on liquid-liquid phase separation in protein condensates: physical chemistry and biological implications. *Chemistry* **25**, 13049–13069 (2019). 31237369 10.1002/chem.201902210

[148] Tapon, N. et al. salvador Promotes both cell cycle exit and apoptosis in Drosophila and is mutated in human cancer cell lines. *Cell* **110**, 467–478 (2002). 12202036 10.1016/s0092-8674(02)00824-3

[149] Dong, J. et al. Elucidation of a universal size-control mechanism in Drosophila and mammals. *Cell* **130**, 1120–1133 (2007). 17889654 10.1016/j.cell.2007.07.019 PMC2666353

[150] Xu, T., Wang, W., Zhang, S., Stewart, R. A. & Yu, W. Identifying tumor suppressors in genetic mosaics: the Drosophila lats gene encodes a putative protein kinase. *Development* **121**, 1053–1063 (1995). 7743921 10.1242/dev.121.4.1053

[151] Barolo, S. & Posakony, J. W. Three habits of highly effective signaling pathways: principles of transcriptional control by developmental cell signaling. *Genes Dev.* **16**, 1167–1181 (2002). 12023297 10.1101/gad.976502

[152] Harvey, K. F., Zhang, X. & Thomas, D. M. The Hippo pathway and human cancer. *Nat. Rev. Cancer* **13**, 246–257 (2013). 23467301 10.1038/nrc3458

[153] Zheng, Y. & Pan, D. The Hippo signaling pathway in development and disease. *Dev. Cell* **50**, 264–282 (2019). 31386861 10.1016/j.devcel.2019.06.003 PMC6748048

[154] Huang, J., Wu, S., Barrera, J., Matthews, K. & Pan, D. The Hippo signaling pathway coordinately regulates cell proliferation and apoptosis by inactivating Yorkie, the Drosophila Homolog of YAP. *Cell* **122**, 421–434 (2005). 16096061 10.1016/j.cell.2005.06.007

[155] Kanai, F. et al. TAZ: a novel transcriptional co-activator regulated by interactions with 14-3-3 and PDZ domain proteins. *EMBO J.* **19**, 6778–6791 (2000). 11118213 10.1093/emboj/19.24.6778 PMC305881

[156] Hong, W. & Guan, K. L. The YAP and TAZ transcription co-activators: key downstream effectors of the mammalian Hippo pathway. *Semin Cell Dev. Biol.* **23**, 785–793 (2012). 22659496 10.1016/j.semcdb.2012.05.004 PMC3459069

[157] Piccolo, S., Dupont, S. & Cordenonsi, M. The biology of YAP/TAZ: hippo signaling and beyond. *Physiol. Rev.* **94**, 1287–1312 (2014). 25287865 10.1152/physrev.00005.2014

[158] Hansen, C. G., Moroishi, T. & Guan, K. L. YAP and TAZ: a nexus for Hippo signaling and beyond. *Trends Cell Biol.* **25**, 499–513 (2015). 26045258 10.1016/j.tcb.2015.05.002 PMC4554827

[159] Wu, S., Huang, J., Dong, J. & Pan, D. Hippo encodes a Ste-20 family protein kinase that restricts cell proliferation and promotes apoptosis in conjunction with salvador and warts. *Cell* **114**, 445–456 (2003). 12941273 10.1016/s0092-8674(03)00549-x

[160] Udan, R. S., Kango-Singh, M., Nolo, R., Tao, C. & Halder, G. Hippo promotes proliferation arrest and apoptosis in the Salvador/Warts pathway. *Nat. Cell Biol.* **5**, 914–920 (2003). 14502294 10.1038/ncb1050

[161] Chan, E. H. et al. The Ste20-like kinase Mst2 activates the human large tumor suppressor kinase Lats1. *Oncogene* **24**, 2076–2086 (2005). 15688006 10.1038/sj.onc.1208445

[162] Callus, B. A., Verhagen, A. M. & Vaux, D. L. Association of mammalian sterile twenty kinases, Mst1 and Mst2, with hSalvador via C-terminal coiled-coil domains, leads to its stabilization and phosphorylation. *FEBS J.* **273**, 4264–4276 (2006). 16930133 10.1111/j.1742-4658.2006.05427.x

[163] Praskova, M., Xia, F. & Avruch, J. MOBKL1A/MOBKL1B phosphorylation by MST1 and MST2 inhibits cell proliferation. *Curr. Biol.* **18**, 311–321 (2008). 18328708 10.1016/j.cub.2008.02.006 PMC4682548

[164] Liu, C. Y. et al. The hippo tumor pathway promotes TAZ degradation by phosphorylating a phosphodegron and recruiting the SCF{beta}-TrCP E3 ligase. *J. Biol. Chem.* **285**, 37159–37169 (2010). 20858893 10.1074/jbc.M110.152942 PMC2988322

[165] Zhao, B., Li, L., Tumaneng, K., Wang, C. Y. & Guan, K. L. A coordinated phosphorylation by Lats and CK1 regulates YAP stability through SCF(beta-TRCP). *Genes Dev.* **24**, 72–85 (2010). 20048001 10.1101/gad.1843810 PMC2802193

[166] Huang, W. et al. The N-terminal phosphodegron targets TAZ/WWTR1 protein for SCFβ-TrCP-dependent degradation in response to phosphatidylinositol 3-kinase inhibition. *J. Biol. Chem.* **287**, 26245–26253 (2012). 22692215 10.1074/jbc.M112.382036 PMC3406709

[167] Hamaratoglu, F. et al. The tumour-suppressor genes NF2/Merlin and Expanded act through Hippo signalling to regulate cell proliferation and apoptosis. *Nat. Cell Biol.* **8**, 27–36 (2006). 16341207 10.1038/ncb1339

[168] Yu, J. et al. Kibra functions as a tumor suppressor protein that regulates Hippo signaling in conjunction with Merlin and Expanded. *Dev. Cell* **18**, 288–299 (2010). 20159598 10.1016/j.devcel.2009.12.012 PMC2858562

[169] Genevet, A., Wehr, M. C., Brain, R., Thompson, B. J. & Tapon, N. Kibra is a regulator of the Salvador/Warts/Hippo signaling network. *Dev. Cell* **18**, 300–308 (2010). 20159599 10.1016/j.devcel.2009.12.011 PMC2845807

[170] Baumgartner, R., Poernbacher, I., Buser, N., Hafen, E. & Stocker, H. The WW domain protein Kibra acts upstream of Hippo in Drosophila. *Dev. Cell* **18**, 309–316 (2010). 20159600 10.1016/j.devcel.2009.12.013

[171] Varelas, X. et al. The Crumbs complex couples cell density sensing to Hippo-dependent control of the TGF-β-SMAD pathway. *Dev. Cell* **19**, 831–844 (2010). 21145499 10.1016/j.devcel.2010.11.012

[172] Zheng, Y. et al. Homeostatic control of Hpo/MST kinase activity through autophosphorylation-dependent recruitment of the STRIPAK PP2A phosphatase complex. *Cell Rep.* **21**, 3612–3623 (2017). 29262338 10.1016/j.celrep.2017.11.076 PMC5741103

[173] Tang, Y. et al. Architecture, substructures, and dynamic assembly of STRIPAK complexes in Hippo signaling. *Cell Discov.* **5**, 3 (2019). 30622739 10.1038/s41421-018-0077-3 PMC6323126

[174] Liu, B. et al. Toll receptor-mediated Hippo signaling controls innate immunity in Drosophila. *Cell* **164**, 406–419 (2016). 26824654 10.1016/j.cell.2015.12.029 PMC4733248

[175] Bae, S. J. et al. SAV1 promotes Hippo kinase activation through antagonizing the PP2A phosphatase STRIPAK. *Elife* **6**, e30278 (2017). 29063833 10.7554/eLife.30278 PMC5663475

[176] Meng, Z., Moroishi, T. & Guan, K. L. Mechanisms of Hippo pathway regulation. *Genes Dev.* **30**, 1–17 (2016). 26728553 10.1101/gad.274027.115 PMC4701972

[177] Wang, L. et al. Multiphase coalescence mediates Hippo pathway activation. *Cell* **185**, 4376–4393.e4318 (2022). 36318920 10.1016/j.cell.2022.09.036 PMC9669202

[178] Moon, S. et al. Phosphorylation by NLK inhibits YAP-14-3-3-interactions and induces its nuclear localization. *EMBO Rep.* **18**, 61–71 (2017). 27979972 10.15252/embr.201642683 PMC5210122

[179] Garcia, K., Gingras, A. C., Harvey, K. F. & Tanas, M. R. TAZ/YAP fusion proteins: mechanistic insights and therapeutic opportunities. Trends. *Cancer* **8**, 1033–1045 (2022). 10.1016/j.trecan.2022.08.002 PMC9671862 36096997

[180] Szulzewsky, F., Holland, E. C. & Vasioukhin, V. YAP1 and its fusion proteins in cancer initiation, progression and therapeutic resistance. *Dev. Biol.* **475**, 205–221 (2021). 33428889 10.1016/j.ydbio.2020.12.018 PMC8107117

[181] Tuna, M., Amos, C. I. & Mills, G. B. Molecular mechanisms and pathobiology of oncogenic fusion transcripts in epithelial tumors. *Oncotarget* **10**, 2095–2111 (2019). 31007851 10.18632/oncotarget.26777 PMC6459343

[182] Pajtler, K. W. et al. YAP1 subgroup supratentorial ependymoma requires TEAD and nuclear factor I-mediated transcriptional programmes for tumorigenesis. *Nat. Commun.* **10**, 3914 (2019). 31477715 10.1038/s41467-019-11884-5 PMC6718408

[183] Szulzewsky, F. et al. Comparison of tumor-associated YAP1 fusions identifies a recurrent set of functions critical for oncogenesis. *Genes Dev.* **34**, 1051–1064 (2020). 32675324 10.1101/gad.338681.120 PMC7397849

[184] Pollack, I. F., Agnihotri, S. & Broniscer, A. Childhood brain tumors: current management, biological insights, and future directions. *J. Neurosurg. Pediatr.* **23**, 261–273 (2019). 30835699 10.3171/2018.10.PEDS18377 PMC6823600

[185] Takadera, M. et al. Phenotypic characterization with somatic genome editing and gene transfer reveals the diverse oncogenicity of ependymoma fusion genes. *Acta Neuropathol. Commun.* **8**, 203 (2020). 33228790 10.1186/s40478-020-01080-8 PMC7684901

[186] Hu, X. et al. Nuclear condensates of YAP fusion proteins alter transcription to drive ependymoma tumourigenesis. *Nat. Cell Biol.* **25**, 323–336 (2023). 36732631 10.1038/s41556-022-01069-6

[187] Zhao, Z. et al. Comprehensive RNA-seq transcriptomic profiling in the malignant progression of gliomas. *Sci. Data* **4**, 170024 (2017). 28291232 10.1038/sdata.2017.24 PMC5349247

[188] Wang, X. et al. Targeting the splicing factor NONO inhibits GBM progression through GPX1 intron retention. *Theranostics* **12**, 5451–5469 (2022). 35910786 10.7150/thno.72248 PMC9330516

[189] Wei, Y. et al. Paraspeckle protein NONO promotes TAZ phase separation in the nucleus to drive the oncogenic transcriptional program. *Adv. Sci.* **8**, e2102653 (2021). 10.1002/advs.202102653 PMC8693076 34716691

[190] Zhou, Y. et al. Up-regulation of ITCH is associated with down-regulation of LATS1 during tumorigenesis and progression of cervical squamous cell carcinoma. *Clin. Investig. Med.* **37**, E384–E394 (2014). 25618271 10.25011/cim.v37i6.22243

[191] Morinaga, N. et al. Molecular analysis of the h-warts/LATS1 gene in human breast cancer. *Int. J. Oncol.* **17**, 1125–1129 (2000). 11078797 10.3892/ijo.17.6.1125

[192] Li, H. et al. Deregulation of Hippo kinase signalling in human hepatic malignancies. *Liver Int* **32**, 38–47 (2012). 22098159 10.1111/j.1478-3231.2011.02646.x PMC4175712

[193] Bridges, M. C., Daulagala, A. C. & Kourtidis, A. LNCcation: lncRNA localization and function. *J. Cell Biol.* **220**, e202009045 (2021). 33464299 10.1083/jcb.202009045 PMC7816648

[194] Wang, M., Xu, T., Feng, W., Liu, J. & Wang, Z. Advances in understanding the LncRNA-mediated regulation of the Hippo pathway in cancer. *Onco Targets Ther.* **14**, 2397–2415 (2021). 33854336 10.2147/OTT.S283157 PMC8039192

[195] Li, R. H. et al. A phosphatidic acid-binding lncRNA SNHG9 facilitates LATS1 liquid-liquid phase separation to promote oncogenic YAP signaling. *Cell Res* **31**, 1088–1105 (2021). 34267352 10.1038/s41422-021-00530-9 PMC8486796

[196] Qin, M. et al. LATS2 condensates organize signalosomes for Hippo pathway signal transduction. *Nat. Chem. Biol.* **20**, 710–720 (2024). 38200110 10.1038/s41589-023-01516-x

[197] Zhou, D. et al. Mst1 and Mst2 maintain hepatocyte quiescence and suppress hepatocellular carcinoma development through inactivation of the Yap1 oncogene. *Cancer Cell* **16**, 425–438 (2009). 19878874 10.1016/j.ccr.2009.09.026 PMC3023165

[198] Song, H. et al. Mammalian Mst1 and Mst2 kinases play essential roles in organ size control and tumor suppression. *Proc. Natl Acad. Sci. USA* **107**, 1431–1436 (2010). 20080598 10.1073/pnas.0911409107 PMC2824397

[199] Liu, Q. et al. Glycogen accumulation and phase separation drives liver tumor initiation. *Cell* **184**, 5559–5576.e5519 (2021). 34678143 10.1016/j.cell.2021.10.001

[200] Ikonomidis, I., Makavos, G. & Lekakis, J. Arterial stiffness and coronary artery disease. *Curr. Opin. Cardiol.* **30**, 422–431 (2015). 26049393 10.1097/HCO.0000000000000179

[201] Garnier, A. S. & Briet, M. Arterial stiffness and chronic kidney disease. *Pulse* **3**, 229–241 (2016). 27195244 10.1159/000443616 PMC4865068

[202] Tang, B. et al. Relationship between Arterial Stiffness and Renal Function Determined by Chronic Kidney Disease Epidemiology Collaboration (CKD-EPI) and Modification of Diet in Renal Disease (MDRD) Equations in a Chinese Cohort Undergoing Health Examination. *Biomed. Res Int.* **2022**, 8218053 (2022). 35321070 10.1155/2022/8218053 PMC8938063

[203] Sági, B. et al. Relationship between arterial stiffness, left ventricular diastolic function, and renal function in chronic kidney disease. *BMC Nephrol.* **24**, 261 (2023). 37661275 10.1186/s12882-023-03308-w PMC10476356

[204] Ngai, D. et al. DDR1 (Discoidin Domain Receptor-1)-RhoA (Ras Homolog Family Member A) axis senses matrix stiffness to promote vascular calcification. *Arterioscler Thromb. Vasc. Biol.* **40**, 1763–1776 (2020). 32493168 10.1161/ATVBAHA.120.314697 PMC7310304

[205] Ngai, D., Mohabeer, A. L., Mao, A., Lino, M. & Bendeck, M. P. Stiffness-responsive feedback autoregulation of DDR1 expression is mediated by a DDR1-YAP/TAZ axis. *Matrix Biol.* **110**, 129–140 (2022). 35562016 10.1016/j.matbio.2022.05.004

[206] Liu, J. et al. Liquid-liquid phase separation of DDR1 counteracts the hippo pathway to orchestrate arterial stiffening. *Circ. Res.* **132**, 87–105 (2023). 36475898 10.1161/CIRCRESAHA.122.322113

[207] Jia, Z. et al. A novel NF2 splicing mutant causes neurofibromatosis type 2 via liquid-liquid phase separation with large tumor suppressor and Hippo pathway. *iScience* **25**, 105275 (2022). 36300003 10.1016/j.isci.2022.105275 PMC9589172

[208] Zhang, N. et al. Molecular alterations of the NF2 gene in hepatocellular carcinoma and intrahepatic cholangiocarcinoma. *Oncol. Rep.* **38**, 3650–3658 (2017). 29130106 10.3892/or.2017.6055

[209] Alonso, M. E. et al. Analysis of the NF2 gene in oligodendrogliomas and ependymomas. *Cancer Genet. Cytogenet.* **134**, 1–5 (2002). 11996787 10.1016/s0165-4608(01)00591-x

[210] Schroeder, R. D., Angelo, L. S. & Kurzrock, R. NF2/merlin in hereditary neurofibromatosis 2 versus cancer: biologic mechanisms and clinical associations. *Oncotarget* **5**, 67–77 (2014). 24393766 10.18632/oncotarget.1557 PMC3960189

[211] Meng, F. et al. Induced phase separation of mutant NF2 imprisons the cGAS-STING machinery to abrogate antitumor immunity. *Mol. Cell* **81**, 4147–4164.e4147 (2021). 34453890 10.1016/j.molcel.2021.07.040

[212] Beatty, G. L. & Gladney, W. L. Immune escape mechanisms as a guide for cancer immunotherapy. *Clin. Cancer Res.* **21**, 687–692 (2015). 25501578 10.1158/1078-0432.CCR-14-1860 PMC4334715

[213] Jiang, Y., Chen, M., Nie, H. & Yuan, Y. PD-1 and PD-L1 in cancer immunotherapy: clinical implications and future considerations. *Hum. Vaccin. Immunother.* **15**, 1111–1122 (2019). 30888929 10.1080/21645515.2019.1571892 PMC6605868

[214] Buchbinder, E. I. & Desai, A. CTLA-4 and PD-1 pathways: similarities, differences, and implications of their inhibition. *Am. J. Clin. Oncol.* **39**, 98–106 (2016). 26558876 10.1097/COC.0000000000000239 PMC4892769

[215] Iwai, Y. et al. Involvement of PD-L1 on tumor cells in the escape from host immune system and tumor immunotherapy by PD-L1 blockade. *Proc. Natl Acad. Sci. USA* **99**, 12293–12297 (2002). 12218188 10.1073/pnas.192461099 PMC129438

[216] Dermani, F. K., Samadi, P., Rahmani, G., Kohlan, A. K. & Najafi, R. PD-1/PD-L1 immune checkpoint: potential target for cancer therapy. *J. Cell Physiol.* **234**, 1313–1325 (2019). 30191996 10.1002/jcp.27172

[217] Seidel, J. A., Otsuka, A. & Kabashima, K. Anti-PD-1 and Anti-CTLA-4 therapies in cancer: mechanisms of action, efficacy, and limitations. *Front. Oncol.* **8**, 86 (2018). 29644214 10.3389/fonc.2018.00086 PMC5883082

[218] Yu, M. et al. Interferon-γ induces tumor resistance to anti-PD-1 immunotherapy by promoting YAP phase separation. *Mol. Cell* **81**, 1216–1230.e1219 (2021). 33606996 10.1016/j.molcel.2021.01.010

[219] Ablasser, A. et al. cGAS produces a 2’-5’-linked cyclic dinucleotide second messenger that activates STING. *Nature* **498**, 380–384 (2013). 23722158 10.1038/nature12306 PMC4143541

[220] Diner, E. J. et al. The innate immune DNA sensor cGAS produces a noncanonical cyclic dinucleotide that activates human STING. *Cell Rep.* **3**, 1355–1361 (2013). 23707065 10.1016/j.celrep.2013.05.009 PMC3706192

[221] Gao, P. et al. Cyclic [G(2’,5’)pA(3’,5’)p] is the metazoan second messenger produced by DNA-activated cyclic GMP-AMP synthase. *Cell* **153**, 1094–1107 (2013). 23647843 10.1016/j.cell.2013.04.046 PMC4382009

[222] Zhang, X. et al. Cyclic GMP-AMP containing mixed phosphodiester linkages is an endogenous high-affinity ligand for STING. *Mol. Cell* **51**, 226–235 (2013). 23747010 10.1016/j.molcel.2013.05.022 PMC3808999

[223] Sun, L., Wu, J., Du, F., Chen, X. & Chen, Z. J. Cyclic GMP-AMP synthase is a cytosolic DNA sensor that activates the type I interferon pathway. *Science* **339**, 786–791 (2013). 23258413 10.1126/science.1232458 PMC3863629

[224] Wu, J. et al. Cyclic GMP-AMP is an endogenous second messenger in innate immune signaling by cytosolic DNA. *Science* **339**, 826–830 (2013). 23258412 10.1126/science.1229963 PMC3855410

[225] Chen, Q., Sun, L. & Chen, Z. J. Regulation and function of the cGAS-STING pathway of cytosolic DNA sensing. *Nat. Immunol.* **17**, 1142–1149 (2016). 27648547 10.1038/ni.3558

[226] Burdette, D. L. et al. STING is a direct innate immune sensor of cyclic di-GMP. *Nature* **478**, 515–518 (2011). 21947006 10.1038/nature10429 PMC3203314

[227] Wu, J., Dobbs, N., Yang, K. & Yan, N. Interferon-independent activities of mammalian STING mediate antiviral response and tumor immune evasion. *Immunity* **53**, 115–126.e115 (2020). 32640258 10.1016/j.immuni.2020.06.009 PMC7365768

[228] Cohen, D. et al. Cyclic GMP-AMP signalling protects bacteria against viral infection. *Nature* **574**, 691–695 (2019). 31533127 10.1038/s41586-019-1605-5

[229] Ishikawa, H. & Barber, G. N. STING is an endoplasmic reticulum adaptor that facilitates innate immune signalling. *Nature* **455**, 674–678 (2008). 18724357 10.1038/nature07317 PMC2804933

[230] Abe, T. et al. STING recognition of cytoplasmic DNA instigates cellular defense. *Mol. Cell* **50**, 5–15 (2013). 23478444 10.1016/j.molcel.2013.01.039 PMC3881179

[231] Ishikawa, H., Ma, Z. & Barber, G. N. STING regulates intracellular DNA-mediated, type I interferon-dependent innate immunity. *Nature* **461**, 788–792 (2009). 19776740 10.1038/nature08476 PMC4664154

[232] Barber, G. N. STING-dependent cytosolic DNA sensing pathways. *Trends Immunol.* **35**, 88–93 (2014). 24309426 10.1016/j.it.2013.10.010

[233] Liu, Y. & Pu, F. Updated roles of cGAS-STING signaling in autoimmune diseases. *Front Immunol.* **14**, 1254915 (2023). 37781360 10.3389/fimmu.2023.1254915 PMC10538533

[234] Skopelja-Gardner, S., An, J. & Elkon, K. B. Role of the cGAS-STING pathway in systemic and organ-specific diseases. *Nat. Rev. Nephrol.* **18**, 558–572 (2022). 35732833 10.1038/s41581-022-00589-6 PMC9214686

[235] Hu, Y. et al. Emerging role of the cGAS-STING signaling pathway in autoimmune diseases: Biologic function, mechanisms and clinical prospection. *Autoimmun. Rev.* **21**, 103155 (2022). 35902046 10.1016/j.autrev.2022.103155

[236] Khoo, L. T. & Chen, L. Y. Role of the cGAS-STING pathway in cancer development and oncotherapeutic approaches. *EMBO Rep.* **19**, e46935 (2018). 30446584 10.15252/embr.201846935 PMC6280650

[237] Ahn, J., Konno, H. & Barber, G. N. Diverse roles of STING-dependent signaling on the development of cancer. *Oncogene* **34**, 5302–5308 (2015). 25639870 10.1038/onc.2014.457 PMC4998969

[238] Decout, A., Katz, J. D., Venkatraman, S. & Ablasser, A. The cGAS-STING pathway as a therapeutic target in inflammatory diseases. *Nat. Rev. Immunol.* **21**, 548–569 (2021). 33833439 10.1038/s41577-021-00524-z PMC8029610

[239] Li, Q. & Gao, P. Phase separation in cGAS-STING signaling. *Front. Med.* **17**, 855–866 (2023). 37906339 10.1007/s11684-023-1026-6

[240] Liu, C. et al. Phase separation in cGAS-STING signaling: cytosolic DNA sensing and regulatory functions. *Chembiochem* **24**, e202300147 (2023). 37041126 10.1002/cbic.202300147

[241] Cao, D., Han, X., Fan, X., Xu, R. M. & Zhang, X. Structural basis for nucleosome-mediated inhibition of cGAS activity. *Cell Res* **30**, 1088–1097 (2020). 33051594 10.1038/s41422-020-00422-4 PMC7784699

[242] Du, M. & Chen, Z. J. DNA-induced liquid phase condensation of cGAS activates innate immune signaling. *Science* **361**, 704–709 (2018). 29976794 10.1126/science.aat1022 PMC9417938

[243] Yao, Y., Wang, W. & Chen, C. Mechanisms of phase-separation-mediated cGAS activation revealed by dcFCCS. *PNAS Nexus* **1**, pgac109 (2022). 36741445 10.1093/pnasnexus/pgac109 PMC9896928

[244] Zhou, W., Mohr, L., Maciejowski, J. & Kranzusch, P. J. cGAS phase separation inhibits TREX1-mediated DNA degradation and enhances cytosolic DNA sensing. *Mol. Cell* **81**, 739–755.e737 (2021). 33606975 10.1016/j.molcel.2021.01.024 PMC7899126

[245] Guey, B. et al. BAF restricts cGAS on nuclear DNA to prevent innate immune activation. *Science* **369**, 823–828 (2020). 32792394 10.1126/science.aaw6421

[246] Liu, Z. S. et al. G3BP1 promotes DNA binding and activation of cGAS. *Nat. Immunol.* **20**, 18–28 (2019). 30510222 10.1038/s41590-018-0262-4 PMC8276115

[247] Zhao, M. et al. The stress granule protein G3BP1 promotes pre-condensation of cGAS to allow rapid responses to DNA. *EMBO Rep.* **23**, e53166 (2022). 34779554 10.15252/embr.202153166 PMC8728604

[248] Zhang, J. et al. ZYG11B potentiates the antiviral innate immune response by enhancing cGAS-DNA binding and condensation. *Cell Rep.* **42**, 112278 (2023). 36933219 10.1016/j.celrep.2023.112278

[249] Tao, X. et al. Ku proteins promote DNA binding and condensation of cyclic GMP-AMP synthase. *Cell Rep.* **40**, 111310 (2022). 36070696 10.1016/j.celrep.2022.111310

[250] Shi, C. et al. USP15 promotes cGAS activation through deubiquitylation and liquid condensation. *Nucleic Acids Res* **50**, 11093–11108 (2022). 36243958 10.1093/nar/gkac823 PMC9638925

[251] Gu, H. et al. PCBP2 maintains antiviral signaling homeostasis by regulating cGAS enzymatic activity via antagonizing its condensation. *Nat. Commun.* **13**, 1564 (2022). 35322803 10.1038/s41467-022-29266-9 PMC8943206

[252] Li, Y. et al. SIRT2 negatively regulates the cGAS-STING pathway by deacetylating G3BP1. *EMBO Rep.* **24**, e57500 (2023). 37870259 10.15252/embr.202357500 PMC10702829

[253] Yu, X. et al. The STING phase-separator suppresses innate immune signalling. *Nat. Cell Biol.* **23**, 330–340 (2021). 33833429 10.1038/s41556-021-00659-0

[254] Xu, G. et al. Viral tegument proteins restrict cGAS-DNA phase separation to mediate immune evasion. *Mol. Cell* **81**, 2823–2837.e2829 (2021). 34015248 10.1016/j.molcel.2021.05.002

[255] Bhowmik, D. et al. Cooperative DNA binding mediated by KicGAS/ORF52 oligomerization allows inhibition of DNA-induced phase separation and activation of cGAS. *Nucleic Acids Res* **49**, 9389–9403 (2021). 34387695 10.1093/nar/gkab689 PMC8450086

[256] Zhou, Z. et al. Sensing of cytoplasmic chromatin by cGAS activates innate immune response in SARS-CoV-2 infection. *Signal Transduct. Target. Ther.* **6**, 382 (2021). 34732709 10.1038/s41392-021-00800-3 PMC8564796

[257] Neufeldt, C. J. et al. SARS-CoV-2 infection induces a pro-inflammatory cytokine response through cGAS-STING and NF-κB. *Commun. Biol.* **5**, 45 (2022). 35022513 10.1038/s42003-021-02983-5 PMC8755718

[258] Cai, S. et al. Phase-separated nucleocapsid protein of SARS-CoV-2 suppresses cGAS-DNA recognition by disrupting cGAS-G3BP1 complex. *Signal Transduct. Target Ther.* **8**, 170 (2023). 37100798 10.1038/s41392-023-01420-9 PMC10131525

[259] Domizio, J. D. et al. The cGAS-STING pathway drives type I IFN immunopathology in COVID-19. *Nature* **603**, 145–151 (2022). 35045565 10.1038/s41586-022-04421-w PMC8891013

[260] Zheng, Y. & Gao, C. Phase separation: the robust modulator of innate antiviral signaling and SARS-CoV-2 infection. *Pathogens* **12**, 243 (2023). 36839515 10.3390/pathogens12020243 PMC9962166

[261] Wang, L. et al. Oleic acid dissolves cGAS-DNA phase separation to inhibit immune surveillance. *Adv. Sci.* **10**, e2206820 (2023). 10.1002/advs.202206820 PMC10190586 36950761

[262] Weyand, C. M. & Goronzy, J. J. Aging of the immune system. mechanisms and therapeutic targets. *Ann. Am. Thorac. Soc.* **13**, S422–s428 (2016). 28005419 10.1513/AnnalsATS.201602-095AW PMC5291468

[263] Sadighi Akha, A. A. Aging and the immune system: an overview. *J. Immunol. Methods* **463**, 21–26 (2018). 30114401 10.1016/j.jim.2018.08.005

[264] Minois, N., Carmona-Gutierrez, D. & Madeo, F. Polyamines in aging and disease. *Aging* **3**, 716–732 (2011). 21869457 10.18632/aging.100361 PMC3184975

[265] Wang, L. et al. Spermine enhances antiviral and anticancer responses by stabilizing DNA binding with the DNA sensor cGAS. *Immunity* **56**, 272–288.e277 (2023). 36724787 10.1016/j.immuni.2023.01.001

[266] Qin, Z. et al. Deactylation by SIRT1 enables liquid-liquid phase separation of IRF3/IRF7 in innate antiviral immunity. *Nat. Immunol.* **23**, 1193–1207 (2022). 35879450 10.1038/s41590-022-01269-0

[267] Wang, X. et al. Development of cyclopeptide inhibitors of cGAS targeting protein-DNA interaction and phase separation. *Nat. Commun.* **14**, 6132 (2023). 37783727 10.1038/s41467-023-41892-5 PMC10545747

[268] Millan, F. A., Denhez, F., Kondaiah, P. & Akhurst, R. J. Embryonic gene expression patterns of TGF beta 1, beta 2 and beta 3 suggest different developmental functions in vivo. *Development* **111**, 131–143 (1991). 1707784 10.1242/dev.111.1.131

[269] Pelton, R. W., Saxena, B., Jones, M., Moses, H. L. & Gold, L. I. Immunohistochemical localization of TGF beta 1, TGF beta 2, and TGF beta 3 in the mouse embryo: expression patterns suggest multiple roles during embryonic development. *J. Cell Biol.* **115**, 1091–1105 (1991). 1955457 10.1083/jcb.115.4.1091 PMC2289937

[270] Xu, X. et al. Transforming growth factor-β in stem cells and tissue homeostasis. *Bone Res* **6**, 2 (2018). 29423331 10.1038/s41413-017-0005-4 PMC5802812

[271] Shi, X. et al. TGF-β signaling in the tumor metabolic microenvironment and targeted therapies. *J. Hematol. Oncol.* **15**, 135 (2022). 36115986 10.1186/s13045-022-01349-6 PMC9482317

[272] Chakravarthy, A., Khan, L., Bensler, N. P., Bose, P. & De Carvalho, D. D. TGF-β-associated extracellular matrix genes link cancer-associated fibroblasts to immune evasion and immunotherapy failure. *Nat. Commun.* **9**, 4692 (2018). 30410077 10.1038/s41467-018-06654-8 PMC6224529

[273] Su, J. et al. TGF-β orchestrates fibrogenic and developmental EMTs via the RAS effector RREB1. *Nature* **577**, 566–571 (2020). 31915377 10.1038/s41586-019-1897-5 PMC7450666

[274] Zhao, H., Wei, J. & Sun, J. Roles of TGF-β signaling pathway in tumor microenvirionment and cancer therapy. *Int Immunopharmacol.* **89**, 107101 (2020). 33099067 10.1016/j.intimp.2020.107101

[275] Peng, D., Fu, M., Wang, M., Wei, Y. & Wei, X. Targeting TGF-β signal transduction for fibrosis and cancer therapy. *Mol. Cancer* **21**, 104 (2022). 35461253 10.1186/s12943-022-01569-x PMC9033932

[276] Deng, Z. et al. TGF-β signaling in health, disease, and therapeutics. *Signal Transduct. Target. Ther.* **9**, 61 (2024). 38514615 10.1038/s41392-024-01764-w PMC10958066

[277] Li, J. et al. Smad2/3/4 complex could undergo liquid liquid phase separation and induce apoptosis through TAT in hepatocellular carcinoma. *Cancer Cell Int* **24**, 176 (2024). 38769521 10.1186/s12935-024-03353-x PMC11106862

[278] Gordon, P. M., Hamid, F., Makeyev, E. V. & Houart, C. A conserved role for the ALS-linked splicing factor SFPQ in repression of pathogenic cryptic last exons. *Nat. Commun.* **12**, 1918 (2021). 33771997 10.1038/s41467-021-22098-z PMC7997972

[279] Li, P. et al. High-throughput and proteome-wide discovery of endogenous biomolecular condensates. *Nat. Chem.* **16**, 1101–1112 (2024). 38499848 10.1038/s41557-024-01485-1

[280] Esposito, M. et al. TGF-β-induced DACT1 biomolecular condensates repress Wnt signalling to promote bone metastasis. *Nat. Cell Biol.* **23**, 257–267 (2021). 33723425 10.1038/s41556-021-00641-w PMC7970447

[281] Attisano, L. & Wrana, J. L. Signal integration in TGF-β, WNT, and Hippo pathways. *F1000Prime Rep.* **5**, 17 (2013). 23755364 10.12703/P5-17 PMC3672943

[282] DiDonato, J. A., Mercurio, F. & Karin, M. NF-κB and the link between inflammation and cancer. *Immunol. Rev.* **246**, 379–400 (2012). 22435567 10.1111/j.1600-065X.2012.01099.x

[283] Liu, T., Zhang, L., Joo, D. & Sun, S. C. NF-κB signaling in inflammation. *Signal Transduct. Target. Ther.* **2**, 17023 (2017). 29158945 10.1038/sigtrans.2017.23 PMC5661633

[284] Giuliani, C., Bucci, I. & Napolitano, G. The role of the transcription factor nuclear factor-Kappa B in thyroid autoimmunity and cancer. *Front. Endocrinol.* **9**, 471 (2018). 10.3389/fendo.2018.00471 PMC6110821 30186235

[285] Oeckinghaus, A., Hayden, M. S. & Ghosh, S. Crosstalk in NF-κB signaling pathways. *Nat. Immunol.* **12**, 695–708 (2011). 21772278 10.1038/ni.2065

[286] Dorrington, M. G. & Fraser, I. D. C. NF-κB signaling in macrophages: dynamics, crosstalk, and signal integration. *Front Immunol.* **10**, 705 (2019). 31024544 10.3389/fimmu.2019.00705 PMC6465568

[287] Israël, A. The IKK complex, a central regulator of NF-kappaB activation. *Cold Spring Harb. Perspect. Biol.* **2**, a000158 (2010). 20300203 10.1101/cshperspect.a000158 PMC2829958

[288] Trares, K., Ackermann, J. & Koch, I. The canonical and non-canonical NF-κB pathways and their crosstalk: a comparative study based on Petri nets. *Biosystems* **211**, 104564 (2022). 34688841 10.1016/j.biosystems.2021.104564

[289] Rothwarf, D. M., Zandi, E., Natoli, G. & Karin, M. IKK-gamma is an essential regulatory subunit of the IkappaB kinase complex. *Nature* **395**, 297–300 (1998). 9751060 10.1038/26261

[290] Mauro, C. et al. ABIN-1 binds to NEMO/IKKgamma and co-operates with A20 in inhibiting NF-kappaB. *J. Biol. Chem.* **281**, 18482–18488 (2006). 16684768 10.1074/jbc.M601502200

[291] Sun, S. C. The non-canonical NF-κB pathway in immunity and inflammation. *Nat. Rev. Immunol.* **17**, 545–558 (2017). 28580957 10.1038/nri.2017.52 PMC5753586

[292] Sun, S. C. Non-canonical NF-κB signaling pathway. *Cell Res* **21**, 71–85 (2011). 21173796 10.1038/cr.2010.177 PMC3193406

[293] Huang, N., Dong, H. & Shao, B. Phase separation in immune regulation and immune-related diseases. *J. Mol. Med* **100**, 1427–1440 (2022). 36085373 10.1007/s00109-022-02253-9 PMC9462646

[294] Du, M., Ea, C. K., Fang, Y. & Chen, Z. J. Liquid phase separation of NEMO induced by polyubiquitin chains activates NF-κB. *Mol. Cell* **82**, 2415–2426.e2415 (2022). 35477005 10.1016/j.molcel.2022.03.037 PMC9402427

[295] Li, Y. et al. Sufu limits sepsis-induced lung inflammation via regulating phase separation of TRAF6. *Theranostics* **13**, 3761–3780 (2023). 37441604 10.7150/thno.83676 PMC10334838

[296] Yamaoka, S. et al. Complementation cloning of NEMO, a component of the IkappaB kinase complex essential for NF-kappaB activation. *Cell* **93**, 1231–1240 (1998). 9657155 10.1016/s0092-8674(00)81466-x

[297] Schmidt-Supprian, M. et al. NEMO/IKK gamma-deficient mice model incontinentia pigmenti. *Mol. Cell* **5**, 981–992 (2000). 10911992 10.1016/s1097-2765(00)80263-4

[298] DiRusso, C. J. et al. A conserved core region of the scaffold NEMO is essential for signal-induced conformational change and liquid-liquid phase separation. *J. Biol. Chem.* **299**, 105396 (2023). 37890781 10.1016/j.jbc.2023.105396 PMC10694592

[299] Zhou, Z. et al. Heightened Innate Immune Responses in the Respiratory Tract of COVID-19 Patients. *Cell Host Microbe* **27**, 883–890.e882 (2020). 32407669 10.1016/j.chom.2020.04.017 PMC7196896

[300] Lee, J. S. et al. Immunophenotyping of COVID-19 and influenza highlights the role of type I interferons in development of severe COVID-19. *Sci. Immunol.* **5**, eabd1554 (2020). 32651212 10.1126/sciimmunol.abd1554 PMC7402635

[301] Liao, M. et al. Single-cell landscape of bronchoalveolar immune cells in patients with COVID-19. *Nat. Med.* **26**, 842–844 (2020). 32398875 10.1038/s41591-020-0901-9

[302] Zhu, L. et al. Single-cell sequencing of peripheral mononuclear cells reveals distinct immune response landscapes of COVID-19 and influenza patients. *Immunity* **53**, 685–696.e683 (2020). 32783921 10.1016/j.immuni.2020.07.009 PMC7368915

[303] Gudowska-Sawczuk, M. & Mroczko, B. The role of nuclear factor Kappa B (NF-κB) in development and treatment of COVID-19: review. *Int. J. Mol. Sci.* **23**, 5283 (2022). 35563673 10.3390/ijms23095283 PMC9101079

[304] Nie, Y. et al. SARS-CoV-2 ORF3a positively regulates NF-κB activity by enhancing IKKβ-NEMO interaction. *Virus Res* **328**, 199086 (2023). 36894068 10.1016/j.virusres.2023.199086 PMC10009424

[305] Yang, S. et al. Molecular mechanisms and cellular functions of liquid-liquid phase separation during antiviral immune responses. *Front. Immunol.* **14**, 1162211 (2023). 37251408 10.3389/fimmu.2023.1162211 PMC10210139

[306] Zhou, Q. et al. The role of SARS-CoV-2-mediated NF-κB activation in COVID-19 patients. *Hypertens. Res.* **47**, 375–384 (2024). 37872376 10.1038/s41440-023-01460-2 PMC10838770

[307] Jobe, F., Simpson, J., Hawes, P., Guzman, E. & Bailey, D. Respiratory syncytial virus sequesters NF-κB subunit p65 to cytoplasmic inclusion bodies to inhibit innate immune signaling. *J. Virol.* **94**, 10–1128 (2020). 10.1128/JVI.01380-20 PMC7592213 32878896

[308] Seif, F. et al. The role of JAK-STAT signaling pathway and its regulators in the fate of T helper cells. *Cell Commun. Signal.* **15**, 23 (2017). 28637459 10.1186/s12964-017-0177-y PMC5480189

[309] Xin, P. et al. The role of JAK/STAT signaling pathway and its inhibitors in diseases. *Int. Immunopharmacol.* **80**, 106210 (2020). 31972425 10.1016/j.intimp.2020.106210

[310] Darnell, J. E. Jr STATs and gene regulation. *Science* **277**, 1630–1635 (1997). 9287210 10.1126/science.277.5332.1630

[311] Clark, J. D., Flanagan, M. E. & Telliez, J. B. Discovery and development of Janus kinase (JAK) inhibitors for inflammatory diseases. *J. Med. Chem.* **57**, 5023–5038 (2014). 24417533 10.1021/jm401490p

[312] Bousoik, E. & Montazeri Aliabadi, H. “Do we know jack” about JAK? A closer look at JAK/STAT signaling pathway. *Front. Oncol.* **8**, 287 (2018). 30109213 10.3389/fonc.2018.00287 PMC6079274

[313] Hu, X., Li, J., Fu, M., Zhao, X. & Wang, W. The JAK/STAT signaling pathway: from bench to clinic. *Signal Transduct. Target. Ther.* **6**, 402 (2021). 34824210 10.1038/s41392-021-00791-1 PMC8617206

[314] Agashe, R. P., Lippman, S. M. & Kurzrock, R. JAK: not just another kinase. *Mol. Cancer Ther.* **21**, 1757–1764 (2022). 36252553 10.1158/1535-7163.MCT-22-0323 PMC10441554

[315] Banerjee, S., Biehl, A., Gadina, M., Hasni, S. & Schwartz, D. M. JAK-STAT signaling as a target for inflammatory and autoimmune diseases: current and future prospects. *Drugs* **77**, 521–546 (2017). 28255960 10.1007/s40265-017-0701-9 PMC7102286

[316] Villarino, A. V., Kanno, Y. & O’Shea, J. J. Mechanisms and consequences of Jak-STAT signaling in the immune system. *Nat. Immunol.* **18**, 374–384 (2017). 28323260 10.1038/ni.3691 PMC11565648

[317] Ivashkiv, L. B. & Donlin, L. T. Regulation of type I interferon responses. *Nat. Rev. Immunol.* **14**, 36–49 (2014). 24362405 10.1038/nri3581 PMC4084561

[318] O’Shea, J. J. & Plenge, R. JAK and STAT signaling molecules in immunoregulation and immune-mediated disease. *Immunity* **36**, 542–550 (2012). 22520847 10.1016/j.immuni.2012.03.014 PMC3499974

[319] O’Shea, J. J. et al. The JAK-STAT pathway: impact on human disease and therapeutic intervention. *Annu. Rev. Med.* **66**, 311–328 (2015). 25587654 10.1146/annurev-med-051113-024537 PMC5634336

[320] Dallagi, A. et al. The activating effect of IFN-γ on monocytes/macrophages is regulated by the LIF-trophoblast-IL-10 axis via Stat1 inhibition and Stat3 activation. *Cell Mol. Immunol.* **12**, 326–341 (2015). 25027966 10.1038/cmi.2014.50 PMC4654315

[321] Shapouri-Moghaddam, A. et al. Macrophage plasticity, polarization, and function in health and disease. *J. Cell Physiol.* **233**, 6425–6440 (2018). 29319160 10.1002/jcp.26429 PMC13482560

[322] Liu, Y. C., Zou, X. B., Chai, Y. F. & Yao, Y. M. Macrophage polarization in inflammatory diseases. *Int. J. Biol. Sci.* **10**, 520–529 (2014). 24910531 10.7150/ijbs.8879 PMC4046879

[323] Funes, S. C., Rios, M., Escobar-Vera, J. & Kalergis, A. M. Implications of macrophage polarization in autoimmunity. *Immunology* **154**, 186–195 (2018). 29455468 10.1111/imm.12910 PMC5980179

[324] Wang, L. X., Zhang, S. X., Wu, H. J., Rong, X. L. & Guo, J. M2b macrophage polarization and its roles in diseases. *J. Leukoc. Biol.* **106**, 345–358 (2019). 30576000 10.1002/JLB.3RU1018-378RR PMC7379745

[325] Wang, C. et al. Macrophage polarization and its role in liver disease. *Front. Immunol.* **12**, 803037 (2021). 34970275 10.3389/fimmu.2021.803037 PMC8712501

[326] Marini, R. P. et al. Characterization of hemolytic Escherichia coli strains in ferrets: recognition of candidate virulence factor CNF1. *J. Clin. Microbiol.* **42**, 5904–5908 (2004). 15583337 10.1128/JCM.42.12.5904-5908.2004 PMC535218

[327] Reitzer, L. & Zimmern, P. Rapid growth and metabolism of uropathogenic Escherichia coli in relation to urine composition. *Clin. Microbiol. Rev.* **33**, e00101–19 (2019). 31619395 10.1128/CMR.00101-19 PMC6927312

[328] Du, M. et al. Processive dynamics of the usher assembly platform during uropathogenic Escherichia coli P pilus biogenesis. *Nat. Commun.* **12**, 5207 (2021). 34471127 10.1038/s41467-021-25522-6 PMC8410936

[329] Ho, M., Mettouchi, A., Wilson, B. A. & Lemichez, E. CNF1-like deamidase domains: common Lego bricks among cancer-promoting immunomodulatory bacterial virulence factors. *Pathog. Dis.* **76**, fty045 (2018). 29733372 10.1093/femspd/fty045 PMC6693396

[330] Sun, X. et al. Interactions of bacterial toxin CNF1 and host JAK1/2 driven by liquid-liquid phase separation enhance macrophage polarization. *mBio* **13**, e0114722 (2022). 35766380 10.1128/mbio.01147-22 PMC9426534

[331] Xia, T. et al. Advances in the role of STAT3 in macrophage polarization. *Front. Immunol.* **14**, 1160719 (2023). 37081874 10.3389/fimmu.2023.1160719 PMC10110879

[332] López-Palacios, T. P. & Andersen, J. L. Kinase regulation by liquid-liquid phase separation. *Trends Cell Biol.* **33**, 649–666 (2023). 36528418 10.1016/j.tcb.2022.11.009 PMC10267292

[333] Zhang, W. & Liu, H. T. MAPK signal pathways in the regulation of cell proliferation in mammalian cells. *Cell Res* **12**, 9–18 (2002). 11942415 10.1038/sj.cr.7290105

[334] Thomas, G. M. & Huganir, R. L. MAPK cascade signalling and synaptic plasticity. *Nat. Rev. Neurosci.* **5**, 173–183 (2004). 14976517 10.1038/nrn1346

[335] Blüthgen, N. & Legewie, S. Systems analysis of MAPK signal transduction. *Essays Biochem* **45**, 95–107 (2008). 18793126 10.1042/BSE0450095

[336] Pagès, G. et al. Mitogen-activated protein kinases p42mapk and p44mapk are required for fibroblast proliferation. *Proc. Natl Acad. Sci. USA* **90**, 8319–8323 (1993). 8397401 10.1073/pnas.90.18.8319 PMC47347

[337] Meloche, S. & Pouysségur, J. The ERK1/2 mitogen-activated protein kinase pathway as a master regulator of the G1- to S-phase transition. *Oncogene* **26**, 3227–3239 (2007). 17496918 10.1038/sj.onc.1210414

[338] Sun, Y. et al. Signaling pathway of MAPK/ERK in cell proliferation, differentiation, migration, senescence and apoptosis. *J. Recept. Signal Transduct. Res* **35**, 600–604 (2015). 26096166 10.3109/10799893.2015.1030412

[339] Zarich, N. et al. Grb2 is a negative modulator of the intrinsic Ras-GEF activity of hSos1. *Mol. Biol. Cell* **17**, 3591–3597 (2006). 16760435 10.1091/mbc.E05-12-1104 PMC1525251

[340] Yan, J., Roy, S., Apolloni, A., Lane, A. & Hancock, J. F. Ras isoforms vary in their ability to activate Raf-1 and phosphoinositide 3-kinase. *J. Biol. Chem.* **273**, 24052–24056 (1998). 9727023 10.1074/jbc.273.37.24052

[341] Avruch, J. et al. Ras activation of the Raf kinase: tyrosine kinase recruitment of the MAP kinase cascade. *Recent Prog. Horm. Res.* **56**, 127–155 (2001). 11237210 10.1210/rp.56.1.127

[342] Widmann, C., Gibson, S., Jarpe, M. B. & Johnson, G. L. Mitogen-activated protein kinase: conservation of a three-kinase module from yeast to human. *Physiol. Rev.* **79**, 143–180 (1999). 9922370 10.1152/physrev.1999.79.1.143

[343] Stokoe, D., Macdonald, S. G., Cadwallader, K., Symons, M. & Hancock, J. F. Activation of Raf as a result of recruitment to the plasma membrane. *Science* **264**, 1463–1467 (1994). 7811320 10.1126/science.7811320

[344] Alessi, D. R. et al. Identification of the sites in MAP kinase kinase-1 phosphorylated by p74raf-1. *EMBO J.* **13**, 1610–1619 (1994). 8157000 10.1002/j.1460-2075.1994.tb06424.x PMC394991

[345] Pouysségur, J., Volmat, V. & Lenormand, P. Fidelity and spatio-temporal control in MAP kinase (ERKs) signalling. *Biochem. Pharm.* **64**, 755–763 (2002). 12213567 10.1016/s0006-2952(02)01135-8

[346] Pierce, K. L., Luttrell, L. M. & Lefkowitz, R. J. New mechanisms in heptahelical receptor signaling to mitogen activated protein kinase cascades. *Oncogene* **20**, 1532–1539 (2001). 11313899 10.1038/sj.onc.1204184

[347] Orton, R. J. et al. Computational modelling of the receptor-tyrosine-kinase-activated MAPK pathway. *Biochem J.* **392**, 249–261 (2005). 16293107 10.1042/BJ20050908 PMC1316260

[348] Guo, Y. J. et al. ERK/MAPK signalling pathway and tumorigenesis. *Exp. Ther. Med.* **19**, 1997–2007 (2020). 32104259 10.3892/etm.2020.8454 PMC7027163

[349] Kim, E. K. & Choi, E. J. Pathological roles of MAPK signaling pathways in human diseases. *Biochim. Biophys. Acta* **1802**, 396–405 (2010). 20079433 10.1016/j.bbadis.2009.12.009

[350] Du, W. et al. The mechanism of MAPK signal transduction pathway involved with electroacupuncture treatment for different diseases. *Evid. Based Complement Altern. Med.* **2019**, 8138017 (2019). 10.1155/2019/8138017 PMC6699341 31467579

[351] Bahar, M. E., Kim, H. J. & Kim, D. R. Targeting the RAS/RAF/MAPK pathway for cancer therapy: from mechanism to clinical studies. *Signal Transduct. Target. Ther.* **8**, 455 (2023). 38105263 10.1038/s41392-023-01705-z PMC10725898

[352] Gorelik, G. & Richardson, B. Key role of ERK pathway signaling in lupus. *Autoimmunity* **43**, 17–22 (2010). 19961364 10.3109/08916930903374832 PMC2819407

[353] Lucas, R. M., Luo, L. & Stow, J. L. ERK1/2 in immune signalling. *Biochem Soc. Trans.* **50**, 1341–1352 (2022). 36281999 10.1042/BST20220271 PMC9704528

[354] D’Souza, W. N., Chang, C. F., Fischer, A. M., Li, M. & Hedrick, S. M. The Erk2 MAPK regulates CD8 T cell proliferation and survival. *J. Immunol.* **181**, 7617–7629 (2008). 19017950 10.4049/jimmunol.181.11.7617 PMC2847891

[355] Matsuda, S. et al. Negative feedback loop in T-cell activation through MAPK-catalyzed threonine phosphorylation of LAT. *EMBO J.* **23**, 2577–2585 (2004). 15192708 10.1038/sj.emboj.7600268 PMC449778

[356] Damasio, M. P. et al. Extracellular signal-regulated kinase (ERK) pathway control of CD8+ T cell differentiation. *Biochem J.* **478**, 79–98 (2021). 33305809 10.1042/BCJ20200661 PMC7813476

[357] Hwang, J. R., Byeon, Y., Kim, D. & Park, S. G. Recent insights of T cell receptor-mediated signaling pathways for T cell activation and development. *Exp. Mol. Med.* **52**, 750–761 (2020). 32439954 10.1038/s12276-020-0435-8 PMC7272404

[358] Bunnell, S. C. et al. T cell receptor ligation induces the formation of dynamically regulated signaling assemblies. *J. Cell Biol.* **158**, 1263–1275 (2002). 12356870 10.1083/jcb.200203043 PMC2173229

[359] Chakraborty, A. K. & Weiss, A. Insights into the initiation of TCR signaling. *Nat. Immunol.* **15**, 798–807 (2014). 25137454 10.1038/ni.2940 PMC5226627

[360] Courtney, A. H., Lo, W. L. & Weiss, A. TCR signaling: mechanisms of initiation and propagation. *Trends Biochem Sci.* **43**, 108–123 (2018). 29269020 10.1016/j.tibs.2017.11.008 PMC5801066

[361] Zhang, W., Sloan-Lancaster, J., Kitchen, J., Trible, R. P. & Samelson, L. E. LAT: the ZAP-70 tyrosine kinase substrate that links T cell receptor to cellular activation. *Cell* **92**, 83–92 (1998). 9489702 10.1016/s0092-8674(00)80901-0

[362] Abraham, R. T. & Weiss, A. Jurkat T cells and development of the T-cell receptor signalling paradigm. *Nat. Rev. Immunol.* **4**, 301–308 (2004). 15057788 10.1038/nri1330

[363] Su, X. et al. Phase separation of signaling molecules promotes T cell receptor signal transduction. *Science* **352**, 595–599 (2016). 27056844 10.1126/science.aad9964 PMC4892427

[364] Zeng, L., Palaia, I., Šarić, A. & Su, X. PLCγ1 promotes phase separation of T cell signaling components. *J. Cell Biol.* **220**, e202009154 (2021). 33929486 10.1083/jcb.202009154 PMC8094118

[365] Neel, B. G., Gu, H. & Pao, L. The ‘Shp’ing news: SH2 domain-containing tyrosine phosphatases in cell signaling. *Trends Biochem. Sci.* **28**, 284–293 (2003). 12826400 10.1016/S0968-0004(03)00091-4

[366] Ahmed, T. A. et al. SHP2 drives adaptive resistance to ERK signaling inhibition in molecularly defined subsets of ERK-dependent tumors. *Cell Rep.* **26**, 65–78.e65 (2019). 30605687 10.1016/j.celrep.2018.12.013 PMC6396678

[367] Chang, C. J., Lin, C. F., Chen, B. C., Lin, P. Y. & Chen, C. L. SHP2: the protein tyrosine phosphatase involved in chronic pulmonary inflammation and fibrosis. *IUBMB Life* **74**, 131–142 (2022). 34590785 10.1002/iub.2559

[368] Tajan, M., de Rocca Serra, A., Valet, P., Edouard, T. & Yart, A. SHP2 sails from physiology to pathology. *Eur. J. Med. Genet.* **58**, 509–525 (2015). 26341048 10.1016/j.ejmg.2015.08.005

[369] Bowen, M. E. et al. Loss-of-function mutations in PTPN11 cause metachondromatosis, but not Ollier disease or Maffucci syndrome. *PLoS Genet* **7**, e1002050 (2011). 21533187 10.1371/journal.pgen.1002050 PMC3077396

[370] Digilio, M. C. et al. Grouping of multiple-lentigines/LEOPARD and Noonan syndromes on the PTPN11 gene. *Am. J. Hum. Genet.* **71**, 389–394 (2002). 12058348 10.1086/341528 PMC379170

[371] Tartaglia, M. & Gelb, B. D. Noonan syndrome and related disorders: genetics and pathogenesis. Annu. Rev. *Genom. Hum. Genet.* **6**, 45–68 (2005). 10.1146/annurev.genom.6.080604.162305 16124853

[372] Athota, J. P. et al. Molecular and clinical studies in 107 Noonan syndrome affected individuals with PTPN11 mutations. *BMC Med. Genet.* **21**, 50 (2020). 32164556 10.1186/s12881-020-0986-5 PMC7068896

[373] Hof, P., Pluskey, S., Dhe-Paganon, S., Eck, M. J. & Shoelson, S. E. Crystal structure of the tyrosine phosphatase SHP-2. *Cell* **92**, 441–450 (1998). 9491886 10.1016/s0092-8674(00)80938-1

[374] Marsh-Armstrong, B., Fajnzylber, J. M., Korntner, S., Plaman, B. A. & Bishop, A. C. The allosteric site on SHP2’s protein tyrosine phosphatase domain is targetable with druglike small molecules. *ACS Omega* **3**, 15763–15770 (2018). 30533581 10.1021/acsomega.8b02200 PMC6275946

[375] Zhu, G. et al. Phase separation of disease-associated SHP2 mutants underlies MAPK hyperactivation. *Cell* **183**, 490–502.e418 (2020). 33002410 10.1016/j.cell.2020.09.002 PMC7572904

[376] Nusse, R. & Clevers, H. Wnt/β-catenin signaling, disease, and emerging therapeutic modalities. *Cell* **169**, 985–999 (2017). 28575679 10.1016/j.cell.2017.05.016

[377] Clevers, H. & Nusse, R. Wnt/β-catenin signaling and disease. *Cell* **149**, 1192–1205 (2012). 22682243 10.1016/j.cell.2012.05.012

[378] Duchartre, Y., Kim, Y. M. & Kahn, M. The Wnt signaling pathway in cancer. *Crit. Rev. Oncol. Hematol.* **99**, 141–149 (2016). 26775730 10.1016/j.critrevonc.2015.12.005 PMC5853106

[379] Li, D., Sun, J. & Zhong, T. P. Wnt Signaling in Heart Development and Regeneration. *Curr. Cardiol. Rep.* **24**, 1425–1438 (2022). 35925512 10.1007/s11886-022-01756-8

[380] Steinhart, Z. & Angers, S. Wnt signaling in development and tissue homeostasis. *Development* **145**, dev146589 (2018). 29884654 10.1242/dev.146589

[381] Perugorria, M. J. et al. Wnt-β-catenin signalling in liver development, health and disease. *Nat. Rev. Gastroenterol. Hepatol.* **16**, 121–136 (2019). 30451972 10.1038/s41575-018-0075-9

[382] Baron, R. & Kneissel, M. WNT signaling in bone homeostasis and disease: from human mutations to treatments. *Nat. Med.* **19**, 179–192 (2013). 23389618 10.1038/nm.3074

[383] Niehrs, C. The complex world of WNT receptor signalling. *Nat. Rev. Mol. Cell Biol.* **13**, 767–779 (2012). 23151663 10.1038/nrm3470

[384] Malbon, C. C. & Wang, H. Y. Dishevelled: a mobile scaffold catalyzing development. *Curr. Top. Dev. Biol.* **72**, 153–166 (2006). 16564334 10.1016/S0070-2153(05)72002-0

[385] MacDonald, B. T., Tamai, K. & He, X. Wnt/beta-catenin signaling: components, mechanisms, and diseases. *Dev. Cell* **17**, 9–26 (2009). 19619488 10.1016/j.devcel.2009.06.016 PMC2861485

[386] Angers, S. & Moon, R. T. Proximal events in Wnt signal transduction. *Nat. Rev. Mol. Cell Biol.* **10**, 468–477 (2009). 19536106 10.1038/nrm2717

[387] Tamai, K. et al. A mechanism for Wnt coreceptor activation. *Mol. Cell* **13**, 149–156 (2004). 14731402 10.1016/s1097-2765(03)00484-2

[388] Zeng, X. et al. A dual-kinase mechanism for Wnt co-receptor phosphorylation and activation. *Nature* **438**, 873–877 (2005). 16341017 10.1038/nature04185 PMC2100418

[389] Lee, K. et al. Receptor heterodimerization as a novel mechanism for the regulation of Wnt/β-catenin signaling. *J. Cell Sci.* **127**, 4857–4869 (2014). 25271056 10.1242/jcs.149302 PMC4231303

[390] Lee, E., Salic, A., Krüger, R., Heinrich, R. & Kirschner, M. W. The roles of APC and Axin derived from experimental and theoretical analysis of the Wnt pathway. *PLoS Biol.* **1**, E10 (2003). 14551908 10.1371/journal.pbio.0000010 PMC212691

[391] Waaler, J. et al. A novel tankyrase inhibitor decreases canonical Wnt signaling in colon carcinoma cells and reduces tumor growth in conditional APC mutant mice. *Cancer Res* **72**, 2822–2832 (2012). 22440753 10.1158/0008-5472.CAN-11-3336

[392] Luo, W. & Lin, S. C. Axin: a master scaffold for multiple signaling pathways. *Neurosignals* **13**, 99–113 (2004). 15067197 10.1159/000076563

[393] Ikeda, S. et al. Axin, a negative regulator of the Wnt signaling pathway, forms a complex with GSK-3beta and beta-catenin and promotes GSK-3beta-dependent phosphorylation of beta-catenin. *EMBO J.* **17**, 1371–1384 (1998). 9482734 10.1093/emboj/17.5.1371 PMC1170485

[394] Jernigan, K. K. et al. Gbetagamma activates GSK3 to promote LRP6-mediated beta-catenin transcriptional activity. *Sci. Signal.* **3**, ra37 (2010). 20460648 10.1126/scisignal.2000647 PMC3088111

[395] Wu, D. & Pan, W. GSK3: a multifaceted kinase in Wnt signaling. *Trends Biochem. Sci.* **35**, 161–168 (2010). 19884009 10.1016/j.tibs.2009.10.002 PMC2834833

[396] Taelman, V. F. et al. Wnt signaling requires sequestration of glycogen synthase kinase 3 inside multivesicular endosomes. *Cell* **143**, 1136–1148 (2010). 21183076 10.1016/j.cell.2010.11.034 PMC3022472

[397] Tanneberger, K. et al. Amer1/WTX couples Wnt-induced formation of PtdIns(4,5)P2 to LRP6 phosphorylation. *EMBO J.* **30**, 1433–1443 (2011). 21304492 10.1038/emboj.2011.28 PMC3102280

[398] Hart, M. et al. The F-box protein beta-TrCP associates with phosphorylated beta-catenin and regulates its activity in the cell. *Curr. Biol.* **9**, 207–210 (1999). 10074433 10.1016/s0960-9822(99)80091-8

[399] Latres, E., Chiaur, D. S. & Pagano, M. The human F box protein beta-Trcp associates with the Cul1/Skp1 complex and regulates the stability of beta-catenin. *Oncogene* **18**, 849–854 (1999). 10023660 10.1038/sj.onc.1202653

[400] Sakamoto, I. et al. A novel beta-catenin-binding protein inhibits beta-catenin-dependent Tcf activation and axis formation. *J. Biol. Chem.* **275**, 32871–32878 (2000). 10921920 10.1074/jbc.M004089200

[401] Cavallo, R. A. et al. Drosophila Tcf and Groucho interact to repress Wingless signalling activity. *Nature* **395**, 604–608 (1998). 9783586 10.1038/26982

[402] Nong, J. et al. Phase separation of Axin organizes the β-catenin destruction complex. *J. Cell Biol.* **220**, e202012112 (2021). 33651074 10.1083/jcb.202012112 PMC7931644

[403] Schaefer, K. N. & Peifer, M. Wnt/Beta-catenin signaling regulation and a role for biomolecular condensates. *Dev. Cell* **48**, 429–444 (2019). 30782412 10.1016/j.devcel.2019.01.025 PMC6386181

[404] Shi, Q., Kang, K. & Chen, Y. G. Liquid-liquid phase separation drives the β-catenin destruction complex formation. *Bioessays* **43**, e2100138 (2021). 34418117 10.1002/bies.202100138

[405] Pronobis, M. I., Deuitch, N., Posham, V., Mimori-Kiyosue, Y. & Peifer, M. Reconstituting regulation of the canonical Wnt pathway by engineering a minimal β-catenin destruction machine. *Mol. Biol. Cell* **28**, 41–53 (2017). 27852897 10.1091/mbc.E16-07-0557 PMC5221518

[406] Kunttas-Tatli, E., Roberts, D. M. & McCartney, B. M. Self-association of the APC tumor suppressor is required for the assembly, stability, and activity of the Wnt signaling destruction complex. *Mol. Biol. Cell* **25**, 3424–3436 (2014). 25208568 10.1091/mbc.E14-04-0885 PMC4214788

[407] Schwarz-Romond, T., Metcalfe, C. & Bienz, M. Dynamic recruitment of axin by Dishevelled protein assemblies. *J. Cell Sci.* **120**, 2402–2412 (2007). 17606995 10.1242/jcs.002956

[408] Faux, M. C. et al. Recruitment of adenomatous polyposis coli and beta-catenin to axin-puncta. *Oncogene* **27**, 5808–5820 (2008). 18591934 10.1038/onc.2008.205

[409] Zhang, Y. et al. RNF146 is a poly(ADP-ribose)-directed E3 ligase that regulates axin degradation and Wnt signalling. *Nat. Cell Biol.* **13**, 623–629 (2011). 21478859 10.1038/ncb2222

[410] Huang, S. M. et al. Tankyrase inhibition stabilizes axin and antagonizes Wnt signalling. *Nature* **461**, 614–620 (2009). 19759537 10.1038/nature08356

[411] Kim, D. Y. et al. Tankyrase-selective inhibitor STP1002 shows preclinical antitumour efficacy without on-target toxicity in the gastrointestinal tract. *Eur. J. Cancer* **173**, 41–51 (2022). 35849876 10.1016/j.ejca.2022.06.031

[412] Klement, K., Brückner, M. & Bernkopf, D. B. Phosphorylation of axin within biomolecular condensates counteracts its tankyrase-mediated degradation. *J. Cell Sci.* **136**, jcs261214 (2023). 37721093 10.1242/jcs.261214 PMC10652037

[413] Zhang, D. et al. APC mutations disrupt β-catenin destruction complex condensates organized by Axin phase separation. *Cell Mol. Life Sci.* **81**, 57 (2024). 38279052 10.1007/s00018-023-05068-0 PMC10817841

[414] Wang, Y. et al. USP10 strikes down β-catenin by dual-wielding deubiquitinase activity and phase separation potential. *Cell Chem. Biol.* **30**, 1436–1452.e1410 (2023). 37611590 10.1016/j.chembiol.2023.07.016

[415] Mendoza-Topaz, C., Mieszczanek, J. & Bienz, M. The Adenomatous polyposis coli tumour suppressor is essential for Axin complex assembly and function and opposes Axin’s interaction with Dishevelled. *Open Biol.* **1**, 110013 (2011). 22645652 10.1098/rsob.110013 PMC3352083

[416] Roberts, D. M. et al. Deconstructing the ßcatenin destruction complex: mechanistic roles for the tumor suppressor APC in regulating Wnt signaling. *Mol. Biol. Cell* **22**, 1845–1863 (2011). 21471006 10.1091/mbc.E10-11-0871 PMC3103401

[417] Li, T. M. et al. Multivalent tumor suppressor adenomatous polyposis coli promotes Axin biomolecular condensate formation and efficient β-catenin degradation. *Sci. Rep.* **10**, 17425 (2020). 33060621 10.1038/s41598-020-74080-2 PMC7562749

[418] Bressler, S. G., Mitrany, A., Wenger, A., Näthke, I. & Friedler, A. The oligomerization domains of the APC protein mediate liquid-liquid phase separation that is phosphorylation controlled. *Int. J. Mol. Sci.* **24**, 6478 (2023). 37047451 10.3390/ijms24076478 PMC10095272

[419] Wehrli, M. et al. arrow encodes an LDL-receptor-related protein essential for Wingless signalling. *Nature* **407**, 527–530 (2000). 11029006 10.1038/35035110

[420] Tamai, K. et al. LDL-receptor-related proteins in Wnt signal transduction. *Nature* **407**, 530–535 (2000). 11029007 10.1038/35035117

[421] Pinson, K. I., Brennan, J., Monkley, S., Avery, B. J. & Skarnes, W. C. An LDL-receptor-related protein mediates Wnt signalling in mice. *Nature* **407**, 535–538 (2000). 11029008 10.1038/35035124

[422] Wodarz, A. & Nusse, R. Mechanisms of Wnt signaling in development. *Annu. Rev. Cell Dev. Biol.* **14**, 59–88 (1998). 9891778 10.1146/annurev.cellbio.14.1.59

[423] Janda, C. Y., Waghray, D., Levin, A. M., Thomas, C. & Garcia, K. C. Structural basis of Wnt recognition by Frizzled. *Science* **337**, 59–64 (2012). 22653731 10.1126/science.1222879 PMC3577348

[424] Voronkov, A. E., Baskin, I. I., Palyulin, V. A. & Zefirov, N. S. Molecular modeling of the complex between the xWNT8 protein and the CRD domain of the mFZD8 receptor. *Dokl. Biochem. Biophys.* **412**, 8–11 (2007). 17506343 10.1134/s1607672907010036

[425] DeBruine, Z. J. et al. Wnt5a promotes Frizzled-4 signalosome assembly by stabilizing cysteine-rich domain dimerization. *Genes Dev.* **31**, 916–926 (2017). 28546512 10.1101/gad.298331.117 PMC5458758

[426] Hirai, H., Matoba, K., Mihara, E., Arimori, T. & Takagi, J. Crystal structure of a mammalian Wnt-frizzled complex. *Nat. Struct. Mol. Biol.* **26**, 372–379 (2019). 31036956 10.1038/s41594-019-0216-z

[427] Davidson, G. et al. Casein kinase 1 gamma couples Wnt receptor activation to cytoplasmic signal transduction. *Nature* **438**, 867–872 (2005). 16341016 10.1038/nature04170

[428] He, X., Semenov, M., Tamai, K. & Zeng, X. LDL receptor-related proteins 5 and 6 in Wnt/beta-catenin signaling: arrows point the way. *Development* **131**, 1663–1677 (2004). 15084453 10.1242/dev.01117

[429] Feng, Q. & Gao, N. Keeping Wnt signalosome in check by vesicular traffic. *J. Cell Physiol.* **230**, 1170–1180 (2015). 25336320 10.1002/jcp.24853 PMC4433473

[430] Bilic, J. et al. Wnt induces LRP6 signalosomes and promotes dishevelled-dependent LRP6 phosphorylation. *Science* **316**, 1619–1622 (2007). 17569865 10.1126/science.1137065

[431] Pan, W. et al. Wnt3a-mediated formation of phosphatidylinositol 4,5-bisphosphate regulates LRP6 phosphorylation. *Science* **321**, 1350–1353 (2008). 18772438 10.1126/science.1160741 PMC2532521

[432] Li, Y. et al. Volumetric compression induces intracellular crowding to control intestinal organoid growth via Wnt/β-catenin signaling. *Cell Stem Cell* **28**, 63–78.e67 (2021). 33053374 10.1016/j.stem.2020.09.012 PMC7796961

[433] Boutros, M. & Mlodzik, M. Dishevelled: at the crossroads of divergent intracellular signaling pathways. *Mech. Dev.* **83**, 27–37 (1999). 10507837 10.1016/s0925-4773(99)00046-5

[434] Kang, K., Shi, Q., Wang, X. & Chen, Y. G. Dishevelled phase separation promotes Wnt signalosome assembly and destruction complex disassembly. *J. Cell Biol.* **221**, e202205069 (2022). 36342472 10.1083/jcb.202205069 PMC9811998

[435] Philippi, M. et al. Biofunctional Nanodot Arrays in Living Cells Uncover Synergistic Co-Condensation of Wnt Signalodroplets. *Small* **18**, e2203723 (2022). 36266931 10.1002/smll.202203723

[436] Gammons, M. V., Renko, M., Johnson, C. M., Rutherford, T. J. & Bienz, M. Wnt signalosome assembly by DEP domain swapping of Dishevelled. *Mol. Cell* **64**, 92–104 (2016). 27692984 10.1016/j.molcel.2016.08.026 PMC5065529

[437] Fagotto, F. et al. Domains of axin involved in protein-protein interactions, Wnt pathway inhibition, and intracellular localization. *J. Cell Biol.* **145**, 741–756 (1999). 10330403 10.1083/jcb.145.4.741 PMC2133179

[438] Wallingford, J. B. & Habas, R. The developmental biology of Dishevelled: an enigmatic protein governing cell fate and cell polarity. *Development* **132**, 4421–4436 (2005). 16192308 10.1242/dev.02068

[439] Ponting, C. P., Phillips, C., Davies, K. E. & Blake, D. J. PDZ domains: targeting signalling molecules to sub-membranous sites. *Bioessays* **19**, 469–479 (1997). 9204764 10.1002/bies.950190606

[440] Pan, W. J. et al. Characterization of function of three domains in dishevelled-1: DEP domain is responsible for membrane translocation of dishevelled-1. *Cell Res* **14**, 324–330 (2004). 15353129 10.1038/sj.cr.7290232

[441] Tauriello, D. V. et al. Wnt/β-catenin signaling requires interaction of the Dishevelled DEP domain and C terminus with a discontinuous motif in Frizzled. *Proc. Natl Acad. Sci. USA* **109**, E812–E820 (2012). 22411803 10.1073/pnas.1114802109 PMC3325702

[442] Mund, T. et al. Disinhibition of the HECT E3 ubiquitin ligase WWP2 by polymerized Dishevelled. *Open Biol.* **5**, 150185 (2015). 26701932 10.1098/rsob.150185 PMC4703060

[443] Behrens, J. et al. Functional interaction of beta-catenin with the transcription factor LEF-1. *Nature* **382**, 638–642 (1996). 8757136 10.1038/382638a0

[444] Huber, O. et al. Nuclear localization of beta-catenin by interaction with transcription factor LEF-1. *Mech. Dev.* **59**, 3–10 (1996). 8892228 10.1016/0925-4773(96)00597-7

[445] Zhao, B. et al. LEF1 enhances β-catenin transactivation through IDR-dependent liquid-liquid phase separation. *Life Sci. Alliance* **6**, e202302118 (2023). 37657935 10.26508/lsa.202302118 PMC10474303

[446] Cordle, J. et al. A conserved face of the Jagged/Serrate DSL domain is involved in Notch trans-activation and cis-inhibition. *Nat. Struct. Mol. Biol.* **15**, 849–857 (2008). 18660822 10.1038/nsmb.1457 PMC2669539

[447] Yuan, X. et al. Notch signaling: an emerging therapeutic target for cancer treatment. *Cancer Lett.* **369**, 20–27 (2015). 26341688 10.1016/j.canlet.2015.07.048

[448] Capaccione, K. M. & Pine, S. R. The Notch signaling pathway as a mediator of tumor survival. *Carcinogenesis* **34**, 1420–1430 (2013). 23585460 10.1093/carcin/bgt127 PMC3697894

[449] Groot, A. J. & Vooijs, M. A. The role of Adams in Notch signaling. *Adv. Exp. Med. Biol.* **727**, 15–36 (2012). 22399336 10.1007/978-1-4614-0899-4_2 PMC4050497

[450] Shih Ie, M. & Wang, T. L. Notch signaling, gamma-secretase inhibitors, and cancer therapy. *Cancer Res* **67**, 1879–1882 (2007). 17332312 10.1158/0008-5472.CAN-06-3958

[451] Kitagawa, M. et al. A human protein with sequence similarity to Drosophila mastermind coordinates the nuclear form of Notch and a CSL protein to build a transcriptional activator complex on target promoters. *Mol. Cell Biol.* **21**, 4337–4346 (2001). 11390662 10.1128/MCB.21.13.4337-4346.2001 PMC87094

[452] Jarriault, S. et al. Signalling downstream of activated mammalian Notch. *Nature* **377**, 355–358 (1995). 7566092 10.1038/377355a0

[453] Penton, A. L., Leonard, L. D. & Spinner, N. B. Notch signaling in human development and disease. *Semin. Cell Dev. Biol.* **23**, 450–457 (2012). 22306179 10.1016/j.semcdb.2012.01.010 PMC3638987

[454] Sachan, N., Sharma, V., Mutsuddi, M. & Mukherjee, A. Notch signalling: multifaceted role in development and disease. *FEBS J.* **291**, 3030–3059 (2023). 37166442 10.1111/febs.16815

[455] Li, X. et al. The Notch signaling pathway: a potential target for cancer immunotherapy. *J. Hematol. Oncol.* **16**, 45 (2023). 37131214 10.1186/s13045-023-01439-z PMC10155406

[456] Meurette, O. & Mehlen, P. Notch signaling in the tumor microenvironment. *Cancer Cell* **34**, 536–548 (2018). 30146333 10.1016/j.ccell.2018.07.009

[457] Zhou, B. et al. Notch signaling pathway: architecture, disease, and therapeutics. *Signal Transduct. Target. Ther.* **7**, 95 (2022). 35332121 10.1038/s41392-022-00934-y PMC8948217

[458] Sabari, B. R. et al. Coactivator condensation at super-enhancers links phase separation and gene control. *Science* **361**, eaar3958 (2018). 29930091 10.1126/science.aar3958 PMC6092193

[459] Trojanowski, J. et al. Transcription activation is enhanced by multivalent interactions independent of phase separation. *Mol. Cell* **82**, 1878–1893.e1810 (2022). 35537448 10.1016/j.molcel.2022.04.017

[460] Ahn, J. H. et al. Phase separation drives aberrant chromatin looping and cancer development. *Nature* **595**, 591–595 (2021). 34163069 10.1038/s41586-021-03662-5 PMC8647409

[461] Hnisz, D., Shrinivas, K., Young, R. A., Chakraborty, A. K. & Sharp, P. A. A phase separation model for transcriptional control. *Cell* **169**, 13–23 (2017). 28340338 10.1016/j.cell.2017.02.007 PMC5432200

[462] Foran, G. et al. Notch1 Phase Separation Coupled Percolation facilitates target gene expression and enhancer looping. *Sci. Rep.* **14**, 21912 (2024). 39300145 10.1038/s41598-024-71634-6 PMC11413390

[463] Chen, J. & Zhang, M. The Par3/Par6/aPKC complex and epithelial cell polarity. *Exp. Cell Res.* **319**, 1357–1364 (2013). 23535009 10.1016/j.yexcr.2013.03.021

[464] Lin, D. et al. A mammalian PAR-3-PAR-6 complex implicated in Cdc42/Rac1 and aPKC signalling and cell polarity. *Nat. Cell Biol.* **2**, 540–547 (2000). 10934475 10.1038/35019582

[465] Macara, I. G. Par proteins: partners in polarization. *Curr. Biol.* **14**, R160–R162 (2004). 15027470

[466] Suzuki, A. et al. Atypical protein kinase C is involved in the evolutionarily conserved par protein complex and plays a critical role in establishing epithelia-specific junctional structures. *J. Cell Biol.* **152**, 1183–1196 (2001). 11257119 10.1083/jcb.152.6.1183 PMC2199212

[467] Horikoshi, Y. et al. Interaction between PAR-3 and the aPKC-PAR-6 complex is indispensable for apical domain development of epithelial cells. *J. Cell Sci.* **122**, 1595–1606 (2009). 19401335 10.1242/jcs.043174

[468] Dho, S. E., French, M. B., Woods, S. A. & McGlade, C. J. Characterization of four mammalian numb protein isoforms. Identification of cytoplasmic and membrane-associated variants of the phosphotyrosine binding domain. *J. Biol. Chem.* **274**, 33097–33104 (1999). 10551880 10.1074/jbc.274.46.33097

[469] Belle, V. A., McDermott, N., Meunier, A. & Marignol, L. NUMB inhibition of NOTCH signalling as a therapeutic target in prostate cancer. *Nat. Rev. Urol.* **11**, 499–507 (2014). 25134838 10.1038/nrurol.2014.195 PMC5240474

[470] Lu, B., Rothenberg, M., Jan, L. Y. & Jan, Y. N. Partner of Numb colocalizes with Numb during mitosis and directs Numb asymmetric localization in Drosophila neural and muscle progenitors. *Cell* **95**, 225–235 (1998). 9790529 10.1016/s0092-8674(00)81753-5

[471] Wang, H., Ouyang, Y., Somers, W. G., Chia, W. & Lu, B. Polo inhibits progenitor self-renewal and regulates Numb asymmetry by phosphorylating Pon. *Nature* **449**, 96–100 (2007). 17805297 10.1038/nature06056 PMC3047501

[472] Kelsom, C. & Lu, W. Uncovering the link between malfunctions in Drosophila neuroblast asymmetric cell division and tumorigenesis. *Cell Biosci.* **2**, 38 (2012). 23151376 10.1186/2045-3701-2-38 PMC3524031

[473] Rhyu, M. S., Jan, L. Y. & Jan, Y. N. Asymmetric distribution of numb protein during division of the sensory organ precursor cell confers distinct fates to daughter cells. *Cell* **76**, 477–491 (1994). 8313469 10.1016/0092-8674(94)90112-0

[474] Shan, Z. et al. Basal condensation of Numb and Pon complex via phase transition during Drosophila neuroblast asymmetric division. *Nat. Commun.* **9**, 737 (2018). 29467404 10.1038/s41467-018-03077-3 PMC5821850

[475] Xu, J. et al. Ccdc85c-Par3 condensates couple cell polarity with Notch to control neural progenitor proliferation. *Cell Rep.* **42**, 112677 (2023). 37352102 10.1016/j.celrep.2023.112677

[476] Polak, P. & Hall, M. N. mTOR and the control of whole body metabolism. *Curr. Opin. Cell Biol.* **21**, 209–218 (2009). 19261457 10.1016/j.ceb.2009.01.024

[477] Laplante, M. & Sabatini, D. M. mTOR signaling in growth control and disease. *Cell* **149**, 274–293 (2012). 22500797 10.1016/j.cell.2012.03.017 PMC3331679

[478] Guertin, D. A. & Sabatini, D. M. Defining the role of mTOR in cancer. *Cancer Cell* **12**, 9–22 (2007). 17613433 10.1016/j.ccr.2007.05.008

[479] Peterson, T. R. et al. DEPTOR is an mTOR inhibitor frequently overexpressed in multiple myeloma cells and required for their survival. *Cell* **137**, 873–886 (2009). 19446321 10.1016/j.cell.2009.03.046 PMC2758791

[480] Kim, D. H. et al. mTOR interacts with raptor to form a nutrient-sensitive complex that signals to the cell growth machinery. *Cell* **110**, 163–175 (2002). 12150925 10.1016/s0092-8674(02)00808-5

[481] Zoncu, R., Efeyan, A. & Sabatini, D. M. mTOR: from growth signal integration to cancer, diabetes and ageing. *Nat. Rev. Mol. Cell Biol.* **12**, 21–35 (2011). 21157483 10.1038/nrm3025 PMC3390257

[482] Saxton, R. A. & Sabatini, D. M. mTOR signaling in growth, metabolism, and disease. *Cell* **168**, 960–976 (2017). 28283069 10.1016/j.cell.2017.02.004 PMC5394987

[483] Szwed, A., Kim, E. & Jacinto, E. Regulation and metabolic functions of mTORC1 and mTORC2. *Physiol. Rev.* **101**, 1371–1426 (2021). 33599151 10.1152/physrev.00026.2020 PMC8424549

[484] Das, A. & Reis, F. mTOR signaling: new insights into cancer, cardiovascular diseases, diabetes and aging. *Int. J. Mol. Sci.* **24**, 13628 (2023). 37686434 10.3390/ijms241713628 PMC10487471

[485] Hung, C. M., Garcia-Haro, L., Sparks, C. A. & Guertin, D. A. mTOR-dependent cell survival mechanisms. *Cold Spring Harb. Perspect. Biol.* **4**, a008771 (2012). 23124837 10.1101/cshperspect.a008771 PMC3504431

[486] Boutouja, F., Stiehm, C. M. & Platta, H. W. mTOR: a cellular regulator interface in health and disease. *Cells* **8**, 18 (2019). 30609721 10.3390/cells8010018 PMC6356367

[487] Oshiro, N. et al. The proline-rich Akt substrate of 40 kDa (PRAS40) is a physiological substrate of mammalian target of rapamycin complex 1. *J. Biol. Chem.* **282**, 20329–20339 (2007). 17517883 10.1074/jbc.M702636200 PMC3199301

[488] Sancak, Y. et al. PRAS40 is an insulin-regulated inhibitor of the mTORC1 protein kinase. *Mol. Cell* **25**, 903–915 (2007). 17386266 10.1016/j.molcel.2007.03.003

[489] Jacinto, E. et al. Mammalian TOR complex 2 controls the actin cytoskeleton and is rapamycin insensitive. *Nat. Cell Biol.* **6**, 1122–1128 (2004). 15467718 10.1038/ncb1183

[490] Huang, W. et al. mTORC2 controls actin polymerization required for consolidation of long-term memory. *Nat. Neurosci.* **16**, 441–448 (2013). 23455608 10.1038/nn.3351 PMC3615448

[491] He, Y. et al. Mammalian target of rapamycin and Rictor control neutrophil chemotaxis by regulating Rac/Cdc42 activity and the actin cytoskeleton. *Mol. Biol. Cell* **24**, 3369–3380 (2013). 24006489 10.1091/mbc.E13-07-0405 PMC3814157

[492] Sato, T. et al. Mammalian target of rapamycin (mTOR) complex 2 regulates filamin A-dependent focal adhesion dynamics and cell migration. *Genes Cells* **21**, 579–593 (2016). 27059097 10.1111/gtc.12366

[493] Wullschleger, S., Loewith, R., Oppliger, W. & Hall, M. N. Molecular organization of target of rapamycin complex 2. *J. Biol. Chem.* **280**, 30697–30704 (2005). 16002396 10.1074/jbc.M505553200

[494] Sarbassov, D. D. et al. Rictor, a novel binding partner of mTOR, defines a rapamycin-insensitive and raptor-independent pathway that regulates the cytoskeleton. *Curr. Biol.* **14**, 1296–1302 (2004). 15268862 10.1016/j.cub.2004.06.054

[495] Lamming, D. W. et al. Rapamycin-induced insulin resistance is mediated by mTORC2 loss and uncoupled from longevity. *Science* **335**, 1638–1643 (2012). 22461615 10.1126/science.1215135 PMC3324089

[496] Dibble, C. C. & Cantley, L. C. Regulation of mTORC1 by PI3K signaling. *Trends Cell Biol.* **25**, 545–555 (2015). 26159692 10.1016/j.tcb.2015.06.002 PMC4734635

[497] Menon, S. et al. Spatial control of the TSC complex integrates insulin and nutrient regulation of mTORC1 at the lysosome. *Cell* **156**, 771–785 (2014). 24529379 10.1016/j.cell.2013.11.049 PMC4030681

[498] Dibble, C. C. et al. TBC1D7 is a third subunit of the TSC1-TSC2 complex upstream of mTORC1. *Mol. Cell* **47**, 535–546 (2012). 22795129 10.1016/j.molcel.2012.06.009 PMC3693578

[499] Manning, B. D., Tee, A. R., Logsdon, M. N., Blenis, J. & Cantley, L. C. Identification of the tuberous sclerosis complex-2 tumor suppressor gene product tuberin as a target of the phosphoinositide 3-kinase/akt pathway. *Mol. Cell* **10**, 151–162 (2002). 12150915 10.1016/s1097-2765(02)00568-3

[500] Lynch, C. J., Fox, H. L., Vary, T. C., Jefferson, L. S. & Kimball, S. R. Regulation of amino acid-sensitive TOR signaling by leucine analogues in adipocytes. *J. Cell Biochem.* **77**, 234–251 (2000). 10723090 10.1002/(sici)1097-4644(20000501)77:2<234::aid-jcb7>3.0.co;2-i

[501] Nover, L., Scharf, K. D. & Neumann, D. Cytoplasmic heat shock granules are formed from precursor particles and are associated with a specific set of mRNAs. *Mol. Cell Biol.* **9**, 1298–1308 (1989). 2725500 10.1128/mcb.9.3.1298-1308.1989 PMC362722

[502] Mollet, S. et al. Translationally repressed mRNA transiently cycles through stress granules during stress. *Mol. Biol. Cell* **19**, 4469–4479 (2008). 18632980 10.1091/mbc.E08-05-0499 PMC2555929

[503] Berchtold, D., Battich, N. & Pelkmans, L. A systems-level study reveals regulators of membrane-less organelles in human cells. *Mol. Cell* **72**, 1035–1049.e1035 (2018). 30503769 10.1016/j.molcel.2018.10.036

[504] Gilks, N. et al. Stress granule assembly is mediated by prion-like aggregation of TIA-1. *Mol. Biol. Cell* **15**, 5383–5398 (2004). 15371533 10.1091/mbc.E04-08-0715 PMC532018

[505] Khong, A. et al. The stress granule transcriptome reveals principles of mRNA accumulation in stress granules. *Mol. Cell* **68**, 808–820.e805 (2017). 29129640 10.1016/j.molcel.2017.10.015 PMC5728175

[506] Khong, A. & Parker, R. mRNP architecture in translating and stress conditions reveals an ordered pathway of mRNP compaction. *J. Cell Biol.* **217**, 4124–4140 (2018). 30322972 10.1083/jcb.201806183 PMC6279387

[507] Aulas, A. et al. Stress-specific differences in assembly and composition of stress granules and related foci. *J. Cell Sci.* **130**, 927–937 (2017). 28096475 10.1242/jcs.199240 PMC5358336

[508] Thedieck, K. et al. Inhibition of mTORC1 by astrin and stress granules prevents apoptosis in cancer cells. *Cell* **154**, 859–874 (2013). 23953116 10.1016/j.cell.2013.07.031

[509] Cadena Sandoval, M. et al. mTORC1 crosstalk with stress granules in aging and age-related diseases. *Front. Aging* **2**, 761333 (2021). 35822040 10.3389/fragi.2021.761333 PMC9261333

[510] Kedersha, N. L., Gupta, M., Li, W., Miller, I. & Anderson, P. RNA-binding proteins TIA-1 and TIAR link the phosphorylation of eIF-2 alpha to the assembly of mammalian stress granules. *J. Cell Biol.* **147**, 1431–1442 (1999). 10613902 10.1083/jcb.147.7.1431 PMC2174242

[511] Sidrauski, C., McGeachy, A. M., Ingolia, N. T. & Walter, P. The small molecule ISRIB reverses the effects of eIF2α phosphorylation on translation and stress granule assembly. *Elife* **4**, e05033 (2015). 25719440 10.7554/eLife.05033 PMC4341466

[512] Marmor-Kollet, H. et al. Spatiotemporal proteomic analysis of stress granule disassembly using APEX reveals regulation by SUMOylation and links to ALS pathogenesis. *Mol. Cell* **80**, 876–891.e876 (2020). 33217318 10.1016/j.molcel.2020.10.032 PMC7816607

[513] Stabb, S. D., Cox, D. L. & Harber, J. L. Gender-related therapist attributions in couples therapy: a preliminary multiple case study investigation. *J. Marital Fam. Ther.* **23**, 335–346 (1997). 9373831 10.1111/j.1752-0606.1997.tb01041.x

[514] Dewey, C. M. et al. TDP-43 is directed to stress granules by sorbitol, a novel physiological osmotic and oxidative stressor. *Mol. Cell Biol.* **31**, 1098–1108 (2011). 21173160 10.1128/MCB.01279-10 PMC3067820

[515] Gardner, L. B. Hypoxic inhibition of nonsense-mediated RNA decay regulates gene expression and the integrated stress response. *Mol. Cell Biol.* **28**, 3729–3741 (2008). 18362164 10.1128/MCB.02284-07 PMC2423288

[516] Sfakianos, A. P. et al. The mTOR-S6 kinase pathway promotes stress granule assembly. *Cell Death Differ.* **25**, 1766–1780 (2018). 29523872 10.1038/s41418-018-0076-9 PMC6004310

[517] Kosmas, K. et al. TSC2 interacts with HDLBP/Vigilin and regulates stress granule formation. *Mol. Cancer Res.* **19**, 1389–1397 (2021). 33888601 10.1158/1541-7786.MCR-20-1046

[518] Raught, B. et al. Phosphorylation of eucaryotic translation initiation factor 4B Ser422 is modulated by S6 kinases. *EMBO J.* **23**, 1761–1769 (2004). 15071500 10.1038/sj.emboj.7600193 PMC394247

[519] Rehbein, U. et al. The TSC complex-mTORC1 axis: from lysosomes to stress granules and back. *Front Cell Dev. Biol.* **9**, 751892 (2021). 34778262 10.3389/fcell.2021.751892 PMC8586448

[520] Wippich, F. et al. Dual specificity kinase DYRK3 couples stress granule condensation/dissolution to mTORC1 signaling. *Cell* **152**, 791–805 (2013). 23415227 10.1016/j.cell.2013.01.033

[521] Flegal, K. M., Kruszon-Moran, D., Carroll, M. D., Fryar, C. D. & Ogden, C. L. Trends in obesity among adults in the United States, 2005 to 2014. *JAMA* **315**, 2284–2291 (2016). 27272580 10.1001/jama.2016.6458 PMC11197437

[522] Afshin, A. et al. Health effects of overweight and obesity in 195 countries over 25 years. *N. Engl. J. Med.* **377**, 13–27 (2017). 28604169 10.1056/NEJMoa1614362 PMC5477817

[523] Ogden, C. L. et al. Trends in obesity prevalence among children and adolescents in the United States, 1988-1994 through 2013-2014. *JAMA* **315**, 2292–2299 (2016). 27272581 10.1001/jama.2016.6361 PMC6361521

[524] Petrelli, F. et al. Association of obesity with survival outcomes in patients with cancer: a systematic review and meta-analysis. *JAMA Netw. Open* **4**, e213520 (2021). 33779745 10.1001/jamanetworkopen.2021.3520 PMC8008284

[525] Furukawa, S. et al. Increased oxidative stress in obesity and its impact on metabolic syndrome. *J. Clin. Investig.* **114**, 1752–1761 (2004). 15599400 10.1172/JCI21625 PMC535065

[526] Seo, B. R. et al. Obesity-dependent changes in interstitial ECM mechanics promote breast tumorigenesis. *Sci. Transl. Med.* **7**, 301ra130 (2015). 26290412 10.1126/scitranslmed.3010467 PMC4837896

[527] Kawasaki, N., Asada, R., Saito, A., Kanemoto, S. & Imaizumi, K. Obesity-induced endoplasmic reticulum stress causes chronic inflammation in adipose tissue. *Sci. Rep.* **2**, 799 (2012). 23150771 10.1038/srep00799 PMC3495279

[528] Fonteneau, G. et al. Stress granules determine the development of obesity-associated pancreatic cancer. *Cancer Discov.* **12**, 1984–2005 (2022). 35674408 10.1158/2159-8290.CD-21-1672 PMC9357213

[529] Bergeron-Sandoval, L. P., Safaee, N. & Michnick, S. W. Mechanisms and consequences of macromolecular phase separation. *Cell* **165**, 1067–1079 (2016). 27203111 10.1016/j.cell.2016.05.026

[530] Holehouse, A. S. & Kragelund, B. B. The molecular basis for cellular function of intrinsically disordered protein regions. *Nat. Rev. Mol. Cell Biol.* **25**, 187–211 (2024). 37957331 10.1038/s41580-023-00673-0 PMC11459374

[531] Gallo, C. M., Wang, J. T., Motegi, F. & Seydoux, G. Cytoplasmic partitioning of P granule components is not required to specify the germline in C. elegans. *Science* **330**, 1685–1689 (2010). 21127218 10.1126/science.1193697 PMC3072820

[532] Yang, P. & Zhang, H. You are what you eat: multifaceted functions of autophagy during C. elegans development. *Cell Res* **24**, 80–91 (2014). 24296782 10.1038/cr.2013.154 PMC3879703

[533] Zhang, Y. et al. SEPA-1 mediates the specific recognition and degradation of P granule components by autophagy in C. elegans. *Cell* **136**, 308–321 (2009). 19167332 10.1016/j.cell.2008.12.022

[534] Zhang, G., Wang, Z., Du, Z. & Zhang, H. mTOR regulates phase separation of PGL granules to modulate their autophagic degradation. *Cell* **174**, 1492–1506.e1422 (2018). 30173914 10.1016/j.cell.2018.08.006

[535] Zhou, H. X., Rivas, G. & Minton, A. P. Macromolecular crowding and confinement: biochemical, biophysical, and potential physiological consequences. *Annu. Rev. Biophys.* **37**, 375–397 (2008). 18573087 10.1146/annurev.biophys.37.032807.125817 PMC2826134

[536] Woodruff, J. B. et al. The centrosome is a selective condensate that nucleates microtubules by concentrating tubulin. *Cell* **169**, 1066–1077.e1010 (2017). 28575670 10.1016/j.cell.2017.05.028

[537] Delarue, M. et al. mTORC1 controls phase separation and the biophysical properties of the cytoplasm by tuning crowding. *Cell* **174**, 338–349.e320 (2018). 29937223 10.1016/j.cell.2018.05.042 PMC10080728

[538] Matera, A. G. & Wang, Z. A day in the life of the spliceosome. *Nat. Rev. Mol. Cell Biol.* **15**, 108–121 (2014). 24452469 10.1038/nrm3742 PMC4060434

[539] Shi, Y. Mechanistic insights into precursor messenger RNA splicing by the spliceosome. *Nat. Rev. Mol. Cell Biol.* **18**, 655–670 (2017). 28951565 10.1038/nrm.2017.86

[540] Will, C. L. & Lührmann, R. Spliceosomal UsnRNP biogenesis, structure and function. *Curr. Opin. Cell Biol.* **13**, 290–301 (2001). 11343899 10.1016/s0955-0674(00)00211-8

[541] Fischer, U., Englbrecht, C. & Chari, A. Biogenesis of spliceosomal small nuclear ribonucleoproteins. *Wiley Interdiscip. Rev. RNA* **2**, 718–731 (2011). 21823231 10.1002/wrna.87

[542] Meister, G. & Fischer, U. Assisted RNP assembly: SMN and PRMT5 complexes cooperate in the formation of spliceosomal UsnRNPs. *EMBO J.* **21**, 5853–5863 (2002). 12411503 10.1093/emboj/cdf585 PMC131082

[543] Pellizzoni, L., Yong, J. & Dreyfuss, G. Essential role for the SMN complex in the specificity of snRNP assembly. *Science* **298**, 1775–1779 (2002). 12459587 10.1126/science.1074962

[544] Pánek, J. et al. The SMN complex drives structural changes in human snRNAs to enable snRNP assembly. *Nat. Commun.* **14**, 6580 (2023). 37852981 10.1038/s41467-023-42324-0 PMC10584915

[545] Sleeman, J. E. & Lamond, A. I. Newly assembled snRNPs associate with coiled bodies before speckles, suggesting a nuclear snRNP maturation pathway. *Curr. Biol.* **9**, 1065–1074 (1999). 10531003 10.1016/s0960-9822(99)80475-8

[546] Stanĕk, D. & Neugebauer, K. M. Detection of snRNP assembly intermediates in Cajal bodies by fluorescence resonance energy transfer. *J. Cell Biol.* **166**, 1015–1025 (2004). 15452143 10.1083/jcb.200405160 PMC2172029

[547] Shin, Y. & Brangwynne, C. P. Liquid phase condensation in cell physiology and disease. *Science* **357**, eaaf4382 (2017). 28935776 10.1126/science.aaf4382

[548] Giudice, J. & Jiang, H. Splicing regulation through biomolecular condensates and membraneless organelles. *Nat. Rev. Mol. Cell Biol.* **25**, 686–700 (2024). 10.1038/s41580-024-00739-7 PMC11843573 38773325

[549] Schilling, M. et al. TOR signaling regulates liquid phase separation of the SMN complex governing snRNP biogenesis. *Cell Rep.* **35**, 109277 (2021). 34161763 10.1016/j.celrep.2021.109277

[550] Hnisz, D. et al. Super-enhancers in the control of cell identity and disease. *Cell* **155**, 934–947 (2013). 24119843 10.1016/j.cell.2013.09.053 PMC3841062

[551] Lee, T. I. & Young, R. A. Transcriptional regulation and its misregulation in disease. *Cell* **152**, 1237–1251 (2013). 23498934 10.1016/j.cell.2013.02.014 PMC3640494

[552] Whyte, W. A. et al. Master transcription factors and mediator establish super-enhancers at key cell identity genes. *Cell* **153**, 307–319 (2013). 23582322 10.1016/j.cell.2013.03.035 PMC3653129

[553] Weintraub, H. et al. Activation of muscle-specific genes in pigment, nerve, fat, liver, and fibroblast cell lines by forced expression of MyoD. *Proc. Natl Acad. Sci. USA* **86**, 5434–5438 (1989). 2748593 10.1073/pnas.86.14.5434 PMC297637

[554] David, C. J. & Massagué, J. Contextual determinants of TGFβ action in development, immunity and cancer. *Nat. Rev. Mol. Cell Biol.* **19**, 419–435 (2018). 29643418 10.1038/s41580-018-0007-0 PMC7457231

[555] Rawlings, J. S., Rosler, K. M. & Harrison, D. A. The JAK/STAT signaling pathway. *J. Cell Sci.* **117**, 1281–1283 (2004). 15020666 10.1242/jcs.00963

[556] Zamudio, A. V. et al. Mediator condensates localize signaling factors to key cell identity genes. *Mol. Cell* **76**, 753–766.e756 (2019). 31563432 10.1016/j.molcel.2019.08.016 PMC6898777

[557] Ferrigno, O. et al. Yes-associated protein (YAP65) interacts with Smad7 and potentiates its inhibitory activity against TGF-beta/Smad signaling. *Oncogene* **21**, 4879–4884 (2002). 12118366 10.1038/sj.onc.1205623

[558] Alarcón, C. et al. Nuclear CDKs drive Smad transcriptional activation and turnover in BMP and TGF-beta pathways. *Cell* **139**, 757–769 (2009). 19914168 10.1016/j.cell.2009.09.035 PMC2818353

[559] Pagiatakis, C., Sun, D., Tobin, S. W., Miyake, T. & McDermott, J. C. TGFβ-TAZ/SRF signalling regulates vascular smooth muscle cell differentiation. *FEBS J.* **284**, 1644–1656 (2017). 28342289 10.1111/febs.14070

[560] Huraskin, D. et al. Wnt/β-catenin signaling via Axin2 is required for myogenesis and, together with YAP/Taz and Tead1, active in IIa/IIx muscle fibers. *Development* **143**, 3128–3142 (2016). 27578179 10.1242/dev.139907

[561] Azzolin, L. et al. YAP/TAZ incorporation in the β-catenin destruction complex orchestrates the Wnt response. *Cell* **158**, 157–170 (2014). 24976009 10.1016/j.cell.2014.06.013

[562] Azzolin, L. et al. Role of TAZ as mediator of Wnt signaling. *Cell* **151**, 1443–1456 (2012). 23245942 10.1016/j.cell.2012.11.027

[563] Tripathi, S., Miyake, T., Kelebeev, J. & McDermott, J. C. TAZ exhibits phase separation properties and interacts with Smad7 and β-catenin to repress skeletal myogenesis. *J. Cell Sci.* **135**, jcs259097 (2022). 34859820 10.1242/jcs.259097

[564] Tripathi, S., Miyake, T. & McDermott, J. C. Smad7:β-catenin complex regulates myogenic gene transcription. *Cell Death Dis.* **10**, 387 (2019). 31097718 10.1038/s41419-019-1615-0 PMC6522533

[565] Xie, X. et al. Recent advances in targeting the “undruggable” proteins: from drug discovery to clinical trials. *Signal Transduct. Target. Ther.* **8**, 335 (2023). 37669923 10.1038/s41392-023-01589-z PMC10480221

[566] Zhang, G., Zhang, J., Gao, Y., Li, Y. & Li, Y. Strategies for targeting undruggable targets. *Expert Opin. Drug Discov.* **17**, 55–69 (2022). 34455870 10.1080/17460441.2021.1969359

[567] Dang, C. V., Reddy, E. P., Shokat, K. M. & Soucek, L. Drugging the ‘undruggable’ cancer targets. *Nat. Rev. Cancer* **17**, 502–508 (2017). 28643779 10.1038/nrc.2017.36 PMC5945194

[568] Kang, J., Lim, L., Lu, Y. & Song, J. A unified mechanism for LLPS of ALS/FTLD-causing FUS as well as its modulation by ATP and oligonucleic acids. *PLoS Biol.* **17**, e3000327 (2019). 31188823 10.1371/journal.pbio.3000327 PMC6590835

[569] Tian, Z. & Qian, F. Adenosine triphosphate-induced rapid liquid-liquid phase separation of a model IgG1 mAb. *Mol. Pharm.* **18**, 267–274 (2021). 33307701 10.1021/acs.molpharmaceut.0c00905

[570] Zhou, Q. et al. ATP regulates RNA-driven cold inducible RNA binding protein phase separation. *Protein Sci.* **30**, 1438–1453 (2021). 33991007 10.1002/pro.4123 PMC8197425

[571] Patel, A. et al. ATP as a biological hydrotrope. *Science* **356**, 753–756 (2017). 28522535 10.1126/science.aaf6846

[572] Xu, B., Chen, J. & Liu, Y. Curcumin interacts with α-synuclein condensates to inhibit amyloid aggregation under phase separation. *ACS Omega* **7**, 30281–30290 (2022). 36061735 10.1021/acsomega.2c03534 PMC9434619

[573] Maiti, T. K., Ghosh, K. S. & Dasgupta, S. Interaction of (-)-epigallocatechin-3-gallate with human serum albumin: fluorescence, fourier transform infrared, circular dichroism, and docking studies. *Proteins* **64**, 355–362 (2006). 16705651 10.1002/prot.20995

[574] Bieschke, J. et al. EGCG remodels mature alpha-synuclein and amyloid-beta fibrils and reduces cellular toxicity. *Proc. Natl Acad. Sci. USA* **107**, 7710–7715 (2010). 20385841 10.1073/pnas.0910723107 PMC2867908

[575] Ehrnhoefer, D. E. et al. EGCG redirects amyloidogenic polypeptides into unstructured, off-pathway oligomers. *Nat. Struct. Mol. Biol.* **15**, 558–566 (2008). 18511942 10.1038/nsmb.1437

[576] LeVine, H. Thioflavine T interaction with synthetic Alzheimer’s disease beta-amyloid peptides: detection of amyloid aggregation in solution. *Protein Sci.* **2**, 404–410 (1993). 8453378 10.1002/pro.5560020312 PMC2142377

[577] D’Amico, M. et al. Thioflavin T promotes Aβ(1-40) amyloid fibrils formation. *J. Phys. Chem. Lett.* **3**, 1596–1601 (2012). 26285714 10.1021/jz300412v

[578] Kroschwald, S. et al. Promiscuous interactions and protein disaggregases determine the material state of stress-inducible RNP granules. *Elife* **4**, e06807 (2015). 26238190 10.7554/eLife.06807 PMC4522596

[579] Updike, D. L., Hachey, S. J., Kreher, J. & Strome, S. P granules extend the nuclear pore complex environment in the C. elegans germ line. *J. Cell Biol.* **192**, 939–948 (2011). 21402789 10.1083/jcb.201010104 PMC3063144

[580] Strom, A. R. et al. Phase separation drives heterochromatin domain formation. *Nature* **547**, 241–245 (2017). 28636597 10.1038/nature22989 PMC6022742

[581] Ryu, J. K. et al. Bridging-induced phase separation induced by cohesin SMC protein complexes. *Sci. Adv.* **7**, eabe5905 (2021). 33568486 10.1126/sciadv.abe5905 PMC7875533

[582] Hazawa, M. et al. A light-switching pyrene probe to detect phase-separated biomolecules. *iScience* **24**, 102865 (2021). 34386728 10.1016/j.isci.2021.102865 PMC8346672

[583] Patel, A. et al. Principles and functions of condensate modifying drugs. *Front. Mol. Biosci.* **9**, 1007744 (2022). 36483537 10.3389/fmolb.2022.1007744 PMC9725174

[584] Igelmann, S., Lessard, F. & Ferbeyre, G. Liquid-liquid phase separation in cancer signaling, metabolism and anticancer therapy. *Cancers* **14**, 1830 (2022). 35406602 10.3390/cancers14071830 PMC8997759

[585] Cho, W. K. et al. Mediator and RNA polymerase II clusters associate in transcription-dependent condensates. *Science* **361**, 412–415 (2018). 29930094 10.1126/science.aar4199 PMC6543815

[586] Lyons, H. et al. Functional partitioning of transcriptional regulators by patterned charge blocks. *Cell* **186**, 327–345.e328 (2023). 36603581 10.1016/j.cell.2022.12.013 PMC9910284

[587] Zhang, Y. et al. Overcoming tamoxifen resistance of human breast cancer by targeted gene silencing using multifunctional pRNA nanoparticles. *ACS Nano* **11**, 335–346 (2017). 27966906 10.1021/acsnano.6b05910 PMC5488869

[588] Cui, J. et al. Cross-talk between HER2 and MED1 regulates tamoxifen resistance of human breast cancer cells. *Cancer Res* **72**, 5625–5634 (2012). 22964581 10.1158/0008-5472.CAN-12-1305 PMC4141533

[589] Marmolejo, C. O. et al. A phosphorylation code coordinating transcription condensate dynamics with DNA replication. bioRxiv (2024).

[590] Klein, I. A. et al. Partitioning of cancer therapeutics in nuclear condensates. *Science* **368**, 1386–1392 (2020). 32554597 10.1126/science.aaz4427 PMC7735713

[591] Jordan, P. & Carmo-Fonseca, M. Cisplatin inhibits synthesis of ribosomal RNA in vivo. *Nucleic Acids Res* **26**, 2831–2836 (1998). 9611224 10.1093/nar/26.12.2831 PMC147654

[592] Mao, S. et al. Role of transcriptional cofactors in cardiovascular diseases. *Biochem Biophys. Res Commun.* **706**, 149757 (2024). 38490050 10.1016/j.bbrc.2024.149757

[593] Pascual-Reguant, L. et al. Interactions between BRD4S, LOXL2, and MED1 drive cell cycle transcription in triple-negative breast cancer. *EMBO Mol. Med.* **15**, e18459 (2023). 37937685 10.15252/emmm.202318459 PMC10701626

[594] Wang, Y. et al. Synergistic therapy for cervical cancer by codelivery of cisplatin and JQ1 inhibiting Plk1-mutant Trp53 axis. *Nano Lett.* **21**, 2412–2421 (2021). 33705152 10.1021/acs.nanolett.0c04402

[595] Zanellato, I., Colangelo, D. & Osella, D. JQ1, a BET inhibitor, synergizes with cisplatin and induces apoptosis in highly chemoresistant malignant pleural mesothelioma cells. *Curr. Cancer Drug Targets* **18**, 816–828 (2018). 28669341 10.2174/1568009617666170623101722

[596] Obst, J. K., Tien, A. H., Setiawan, J. C., Deneault, L. F. & Sadar, M. D. Inhibitors of the transactivation domain of androgen receptor as a therapy for prostate cancer. *Steroids* **210**, 109482 (2024). 39053630 10.1016/j.steroids.2024.109482 PMC11364166

[597] Zhang, F. et al. Dynamic phase separation of the androgen receptor and its coactivators key to regulate gene expression. *Nucleic Acids Res* **51**, 99–116 (2023). 36535377 10.1093/nar/gkac1158 PMC9841400

[598] Brand, L. J. et al. EPI-001 is a selective peroxisome proliferator-activated receptor-gamma modulator with inhibitory effects on androgen receptor expression and activity in prostate cancer. *Oncotarget* **6**, 3811–3824 (2015). 25669987 10.18632/oncotarget.2924 PMC4414155

[599] Paulsen, F. O. et al. Targeting cyclin-dependent kinase 7-association between CDK7 and pMED1 expression in prostate cancer tissue. *Carcinogenesis* **43**, 779–786 (2022). 35512686 10.1093/carcin/bgac036

[600] Russo, J. W., Nouri, M. & Balk, S. P. Androgen receptor interaction with mediator complex is enhanced in castration-resistant prostate cancer by CDK7 phosphorylation of MED1. *Cancer Discov.* **9**, 1490–1492 (2019). 31676563 10.1158/2159-8290.CD-19-1028 PMC6830511

[601] Rasool, R. U. et al. CDK7 inhibition suppresses castration-resistant prostate cancer through MED1 inactivation. *Cancer Discov.* **9**, 1538–1555 (2019). 31466944 10.1158/2159-8290.CD-19-0189 PMC7202356

[602] Li, L. et al. Common cancer mutations R175H and R273H drive the p53 DNA-binding domain towards aggregation-prone conformations. *Phys. Chem. Chem. Phys.* **22**, 9225–9232 (2020). 32307496 10.1039/c9cp06671c

[603] Herzog, G. et al. The Lys-specific molecular tweezer, CLR01, modulates aggregation of the mutant p53 DNA binding domain and inhibits its toxicity. *Biochemistry* **54**, 3729–3738 (2015). 26030124 10.1021/bi501092p

[604] Nishitsuji, K. et al. Impacts of cytoplasmic p53 aggregates on the prognosis and the transcriptome in lung squamous cell carcinoma. *Cancer Sci.* **115**, 2947–2960 (2024). 39031627 10.1111/cas.16252 PMC11462941

[605] Haq, B. U. et al. Targeting p53 misfolding conundrum by stabilizing agents and their analogs in breast cancer therapy: a comprehensive computational analysis. *Front. Pharm.* **14**, 1333447 (2023). 10.3389/fphar.2023.1333447 PMC10806237 38269278

[606] Lemos, C. et al. Identification of small molecules that modulate mutant p53 condensation. *iScience* **23**, 101517 (2020). 32927263 10.1016/j.isci.2020.101517 PMC7495113

[607] Chan, K. T. & Lung, M. L. Mutant p53 expression enhances drug resistance in a hepatocellular carcinoma cell line. *Cancer Chemother. Pharm.* **53**, 519–526 (2004). 10.1007/s00280-004-0767-4 15004724

[608] Liu, Q., Li, L., Yu, Y. & Wei, G. Elucidating the mechanisms of R248Q mutation-enhanced p53 aggregation and its inhibition by resveratrol. *J. Phys. Chem. B* **127**, 7708–7720 (2023). 37665658 10.1021/acs.jpcb.3c04700

[609] Hu, J. et al. Targeting mutant p53 for cancer therapy: direct and indirect strategies. *J. Hematol. Oncol.* **14**, 157 (2021). 34583722 10.1186/s13045-021-01169-0 PMC8480024

[610] Miller, J. J., Gaiddon, C. & Storr, T. A balancing act: using small molecules for therapeutic intervention of the p53 pathway in cancer. *Chem. Soc. Rev.* **49**, 6995–7014 (2020). 32869798 10.1039/d0cs00163e

[611] Ma, Z., Bolinger, A. A., Chen, H. & Zhou, J. Drug discovery targeting nuclear receptor binding SET. *domain protein 2 (NSD2). J. Med. Chem.* **66**, 10991–11026 (2023). 37578463 10.1021/acs.jmedchem.3c00948 PMC11092389

[612] Xu, W. X. et al. The burgeoning significance of liquid-liquid phase separation in the pathogenesis and therapeutics of cancers. *Int J. Biol. Sci.* **20**, 1652–1668 (2024). 38481812 10.7150/ijbs.92988 PMC10929184

[613] Liu, J. et al. Targeting NSD2-mediated SRC-3 liquid-liquid phase separation sensitizes bortezomib treatment in multiple myeloma. *Nat. Commun.* **12**, 1022 (2021). 33589584 10.1038/s41467-021-21386-y PMC7884723

[614] Zhang, W. et al. Function of steroid receptor coactivators in T cells and cancers: implications for cancer immunotherapy. *Crit. Rev. Immunol.* **44**, 111–126 (2024). 38848298 10.1615/CritRevImmunol.2024051613 PMC11902286

[615] King, M. R., Ruff, K. M. & Pappu, R. V. Emergent microenvironments of nucleoli. *Nucleus* **15**, 2319957 (2024). 38443761 10.1080/19491034.2024.2319957 PMC10936679

[616] Schmidt, H. B. et al. Oxaliplatin disrupts nucleolar function through biophysical disintegration. *Cell Rep.* **41**, 111629 (2022). 36351392 10.1016/j.celrep.2022.111629 PMC9749789

[617] Mari, M. et al. Bridging pyrimidine hemicurcumin and Cisplatin: synthesis, coordination chemistry, and in vitro activity assessment of a novel Pt(II) complex. *J. Inorg. Biochem* **260**, 112702 (2024). 39163714 10.1016/j.jinorgbio.2024.112702

[618] Ratti, A. & Buratti, E. Physiological functions and pathobiology of TDP-43 and FUS/TLS proteins. *J. Neurochem* **138**, 95–111 (2016). 27015757 10.1111/jnc.13625

[619] Lagier-Tourenne, C., Polymenidou, M. & Cleveland, D. W. TDP-43 and FUS/TLS: emerging roles in RNA processing and neurodegeneration. *Hum. Mol. Genet* **19**, R46–R64 (2010). 20400460 10.1093/hmg/ddq137 PMC3167692

[620] Carey, J. L. & Guo, L. Liquid-liquid phase separation of TDP-43 and FUS in physiology and pathology of neurodegenerative diseases. *Front. Mol. Biosci.* **9**, 826719 (2022). 35187086 10.3389/fmolb.2022.826719 PMC8847598

[621] Kreft, D., Wang, Y., Rattay, M., Toensing, K. & Anselmetti, D. Correction to: Binding mechanism of anti-cancer chemotherapeutic drug mitoxantrone to DNA characterized by magnetic tweezers. *J. Nanobiotechnol.* **17**, 28 (2019). 10.1186/s12951-018-0430-6 PMC6367820 30736797

[622] Milicevic, K., Rankovic, B., Andjus, P. R., Bataveljic, D. & Milovanovic, D. Emerging roles for phase separation of RNA-binding proteins in cellular pathology of ALS. *Front Cell Dev. Biol.* **10**, 840256 (2022). 35372329 10.3389/fcell.2022.840256 PMC8965147

[623] Song, J. Molecular mechanisms of phase separation and amyloidosis of ALS/FTD-linked FUS and TDP-43. *Aging Dis.* **15**, 2084–2112 (2024). 38029395 10.14336/AD.2023.1118 PMC11346406

[624] Koehler, L. C. et al. TDP-43 oligomerization and phase separation properties are necessary for autoregulation. *Front Neurosci.* **16**, 818655 (2022). 35495061 10.3389/fnins.2022.818655 PMC9048411

[625] McDonald, K. K. et al. TAR DNA-binding protein 43 (TDP-43) regulates stress granule dynamics via differential regulation of G3BP and TIA-1. *Hum. Mol. Genet* **20**, 1400–1410 (2011). 21257637 10.1093/hmg/ddr021

[626] Fang, M. Y. et al. Small-molecule modulation of TDP-43 recruitment to stress granules prevents persistent TDP-43 accumulation in ALS/FTD. *Neuron* **103**, 802–819.e811 (2019). 31272829 10.1016/j.neuron.2019.05.048 PMC6728177

[627] Ding, X. et al. Exposure to ALS-FTD-CSF generates TDP-43 aggregates in glioblastoma cells through exosomes and TNTs-like structure. *Oncotarget* **6**, 24178–24191 (2015). 26172304 10.18632/oncotarget.4680 PMC4695178

[628] Tsekrekou, M., Giannakou, M., Papanikolopoulou, K. & Skretas, G. Protein aggregation and therapeutic strategies in SOD1- and TDP-43- linked ALS. *Front Mol. Biosci.* **11**, 1383453 (2024). 38855322 10.3389/fmolb.2024.1383453 PMC11157337

[629] Baralle, M., Buratti, E. & Baralle, F. E. The role of TDP-43 in the pathogenesis of ALS and FTLD. *Biochem Soc. Trans.* **41**, 1536–1540 (2013). 24256250 10.1042/BST20130186

[630] Shenouda, M., Zhang, A. B., Weichert, A. & Robertson, J. Mechanisms associated with TDP-43 neurotoxicity in ALS/FTLD. *Adv. Neurobiol.* **20**, 239–263 (2018). 29916022 10.1007/978-3-319-89689-2_9

[631] Xue, J. et al. XPO1 is a new target of homoharringtonine (HHT): Making NPMc(+) AML cells much more sensitive to HHT treatment. *Biochem Biophys. Res Commun.* **675**, 155–161 (2023). 37473530 10.1016/j.bbrc.2023.07.027

[632] Florio, D. et al. Insights into network of hot spots of aggregation in nucleophosmin 1. *Int. J. Mol. Sci.* **23**, 14704 (2022). 36499032 10.3390/ijms232314704 PMC9736328

[633] Florio, D. et al. Small molecules enhancers of amyloid aggregation of C-terminal domain of Nucleophosmin 1 in acute myeloid leukemia. *Bioorg. Chem.* **127**, 106001 (2022). 35803020 10.1016/j.bioorg.2022.106001

[634] Andresen, V. et al. Anti-proliferative activity of the NPM1 interacting natural product avrainvillamide in acute myeloid leukemia. *Cell Death Dis.* **7**, e2497 (2016). 27906185 10.1038/cddis.2016.392 PMC5260983

[635] Ahn, S. G. & Thiele, D. J. Redox regulation of mammalian heat shock factor 1 is essential for Hsp gene activation and protection from stress. *Genes Dev.* **17**, 516–528 (2003). 12600944 10.1101/gad.1044503 PMC195992

[636] Kmiecik, S. W. & Mayer, M. P. Molecular mechanisms of heat shock factor 1 regulation. *Trends Biochem. Sci.* **47**, 218–234 (2022). 34810080 10.1016/j.tibs.2021.10.004

[637] Dea, A. & Pincus, D. The Heat Shock Response as a Condensate Cascade. *J. Mol. Biol.* **436**, 168642 (2024). 38848866 10.1016/j.jmb.2024.168642 PMC11214683

[638] Wei, H. et al. Heat shock protein 90: biological functions, diseases, and therapeutic targets. *MedComm* **5**, e470 (2024). 38283176 10.1002/mco2.470 PMC10811298

[639] Gaglia, G. et al. HSF1 phase transition mediates stress adaptation and cell fate decisions. *Nat. Cell Biol.* **22**, 151–158 (2020). 32015439 10.1038/s41556-019-0458-3 PMC7135912

[640] Zhang, H. et al. Reversible phase separation of HSF1 is required for an acute transcriptional response during heat shock. *Nat. Cell Biol.* **24**, 340–352 (2022). 35256776 10.1038/s41556-022-00846-7

[641] Mitrea, D. M., Mittasch, M., Gomes, B. F., Klein, I. A. & Murcko, M. A. Modulating biomolecular condensates: a novel approach to drug discovery. *Nat. Rev. Drug Discov.* **21**, 841–862 (2022). 35974095 10.1038/s41573-022-00505-4 PMC9380678

[642] Aulas, A. & Vande Velde, C. Alterations in stress granule dynamics driven by TDP-43 and FUS: a link to pathological inclusions in ALS? *Front Cell Neurosci.* **9**, 423 (2015). 26557057 10.3389/fncel.2015.00423 PMC4615823

[643] Słabicki, M. et al. Small-molecule-induced polymerization triggers degradation of BCL6. *Nature* **588**, 164–168 (2020). 33208943 10.1038/s41586-020-2925-1 PMC7816212

[644] Elshatlawy, M., Sampson, J., Clarke, K. & Bayliss, R. EML4-ALK biology and drug resistance in non-small cell lung cancer: a new phase of discoveries. *Mol. Oncol.* **17**, 950–963 (2023). 37149843 10.1002/1878-0261.13446 PMC10257413

[645] Risso-Ballester, J. et al. A condensate-hardening drug blocks RSV replication in vivo. *Nature* **595**, 596–599 (2021). 34234347 10.1038/s41586-021-03703-z

[646] Wang, J. et al. A model for identification of potential phase-separated proteins based on protein sequence, structure and cellular distribution. *Int J. Biol. Macromol.* **243**, 125196 (2023). 37285890 10.1016/j.ijbiomac.2023.125196

[647] Jia, L., Gao, S. & Qiao, Y. Optical control over liquid–liquid phase separation. *Small Methods* **8**, e2301724 (2024). 38530063 10.1002/smtd.202301724

[648] Babinchak, W. M. et al. Small molecules as potent biphasic modulators of protein liquid-liquid phase separation. *Nat. Commun.* **11**, 5574 (2020). 33149109 10.1038/s41467-020-19211-z PMC7643064

[649] Maruri-Lopez, I. & Chodasiewicz, M. Involvement of small molecules and metabolites in regulation of biomolecular condensate properties. *Curr. Opin. Plant Biol.* **74**, 102385 (2023). 37348448 10.1016/j.pbi.2023.102385

[650] Qiao, X. et al. Discovery of novel and potent dual-targeting AXL/HDAC2 inhibitors for colorectal cancer treatment via structure-based pharmacophore modelling, virtual screening, and molecular docking, molecular dynamics simulation studies, and biological evaluation. *J. Enzym. Inhib. Med Chem.* **39**, 2295241 (2024). 10.1080/14756366.2023.2295241 PMC10763849 38134358

[651] Jin, X. et al. Novel dual-targeting inhibitors of NSD2 and HDAC2 for the treatment of liver cancer: structure-based virtual screening, molecular dynamics simulation, and in vitro and in vivo biological activity evaluations. *J. Enzym. Inhib. Med. Chem.* **39**, 2289355 (2024). 10.1080/14756366.2023.2289355 PMC11721945 38059332

[652] Girdhar, A. et al. Computational insights into mechanism of AIM4-mediated inhibition of aggregation of TDP-43 protein implicated in ALS and evidence for in vitro inhibition of liquid-liquid phase separation (LLPS) of TDP-43(2C)-A315T by AIM4. *Int J. Biol. Macromol.* **147**, 117–130 (2020). 31917988 10.1016/j.ijbiomac.2020.01.032

[653] Sarthak, K., Winogradoff, D., Ge, Y., Myong, S. & Aksimentiev, A. Benchmarking molecular dynamics force fields for all-atom simulations of biological condensates. *J. Chem. Theory Comput* **19**, 3721–3740 (2023). 37134270 10.1021/acs.jctc.3c00148 PMC11169342

[654] Kamagata, K. et al. Rational peptide design for regulating liquid-liquid phase separation on the basis of residue-residue contact energy. *Sci. Rep.* **12**, 13718 (2022). 35962177 10.1038/s41598-022-17829-1 PMC9374670

[655] Dhakal, A., McKay, C., Tanner, J. J. & Cheng, J. Artificial intelligence in the prediction of protein-ligand interactions: recent advances and future directions. *Brief. Bioinform.* **23**, bbab476 (2022). 34849575 10.1093/bib/bbab476 PMC8690157

[656] Hu, W. & Ohue, M. SpatialPPI: Three-dimensional space protein-protein interaction prediction with AlphaFold Multimer. *Comput Struct. Biotechnol. J.* **23**, 1214–1225 (2024). 38545599 10.1016/j.csbj.2024.03.009 PMC10966450

[657] Behara, K., Bhero, E. & Agee, J. T. Grid-based structural and dimensional skin cancer classification with self-featured optimized explainable deep convolutional neural networks. *Int. J. Mol. Sci.* **25**, 1546 (2024). 38338828 10.3390/ijms25031546 PMC10855492

[658] Shen, C. et al. Curvature-enhanced graph convolutional network for biomolecular interaction prediction. *Comput. Struct. Biotechnol. J.* **23**, 1016–1025 (2024). 38425487 10.1016/j.csbj.2024.02.006 PMC10904164

[659] Wang, D. D., Wu, W. & Wang, R. Structure-based, deep-learning models for protein-ligand binding affinity prediction. *J. Cheminform.* **16**, 2 (2024). 38173000 10.1186/s13321-023-00795-9 PMC10765576

[660] Wang, H. Prediction of protein-ligand binding affinity via deep learning models. *Brief. Bioinform.* **25**, bbae081 (2024). 38446737 10.1093/bib/bbae081 PMC10939342

